# The effects of suspension-supported training on dynamic balance capacity in stroke patients: a systematic review and meta-analysis enhanced by XGBoost machine learning

**DOI:** 10.3389/fmed.2026.1747067

**Published:** 2026-02-09

**Authors:** Aibin Cao, Xin Jin, Ying Wang, Jianwei Guo

**Affiliations:** 1 School of Physical Education, Shanxi University, Taiyuan, China; 2 School of Athletic Performance, Shanghai University of Sport, Shanghai, China

**Keywords:** dose–response relationship, dynamic balance, meta-analysis, optimal dose, stroke, suspension-supported training

## Abstract

**Objective:**

Systematically evaluate the effectiveness of suspension-supported training (SST) on dynamic balance in stroke patients and determine its optimal training dose to establish an evidence-based foundation for clinical rehabilitation.

**Methods:**

Following the Preferred Reporting Items for Systematic Reviews and Meta-Analyses (PRISMA) guidelines, randomized controlled trials (RCTs) meeting Population, Intervention, Comparator, Outcome, Study design (PICOS) criteria were screened in PubMed, Web of Science, PsycINFO, and the Cochrane Library (search cutoff: 31 October 2025). Twelve studies comprising 584 patients were included. A restricted maximum likelihood (REML) random-effects model was used to pool standardized mean differences (SMDs). Heterogeneity was assessed using the I-squared (I^2^) statistic and Cochran’s Q test. Publication bias and robustness were evaluated by sensitivity analysis, Egger’s regression test, and the trim-and-fill method. Subgroup analyses and meta-regression were conducted to explore potential moderators; additionally, a Bayesian dose–response meta-analysis was performed to characterize the non-linear relationship between training dosage and dynamic balance outcomes. An exploratory extreme gradient boosting (XGBoost) model combined with SHapley Additive exPlanations (SHAP) analysis was further applied to investigate dose–response patterns and feature importance.

**Results:**

SST produced a significant improvement in dynamic balance (SMD = 0.87, 95% CI 0.49–1.26, *p* < 0.0001), with substantial heterogeneity across studies (I^2^ = 76.6%). The overall quality of evidence was rated as moderate using the Grading of Recommendations Assessment, Development and Evaluation (GRADE) approach. Larger effects were observed in interventions lasting 6–8 weeks, sessions of 40–150 min, patients in the subacute phase, combined intervention approaches, and protocols employing approximately 30% body-weight support. Exploratory XGBoost–SHAP analysis identified training duration and intervention mode as the most influential predictors of outcome. Preliminary dose–response modeling suggested that a regimen of ~90–100 min per session, six sessions per week for 8–9 weeks (total dose ≈ 34.6–37.6 h; ~36 h) might be associated with favorable dynamic-balance gains; however, these estimates should be regarded as hypothesis-generating.

**Conclusion:**

SST effectively enhances dynamic balance in stroke patients. Preliminary models indicated that a regimen of 90–100 min per session, 6 times per week for 8–9 weeks (total duration: 34.6–37.6 h, ~36 h) might yield favorable results. However, given the heterogeneity and study-level limitations, these dosage findings were considered hypothesis-generating. Future research should prioritize high-quality, large-sample RCTs with long follow-up to verify clinical and long-term effectiveness.

## Introduction

Stroke is an acute cerebrovascular disease and ranks as the second leading cause of death worldwide. It is a major cause of long-term disability in adults. The incidence rate of stroke continues to rise significantly, creating a substantial social and economic burden (1). China has the highest number of stroke patients worldwide, and the incidence rate has been rising significantly (2). Studies indicate that over 80% of stroke survivors experience gait impairments. Although gait function usually improves within the first two months after a stroke, most patients still have limited ability to walk in the community (3). Studies show that balance problems after a stroke can occur in up to 83% of cases, while fewer than 10% of stroke survivors have walking speed and endurance enough to handle everyday activities (4). Impaired balance not only greatly affects patients’ daily activities, independence, and quality of life but also raises the risk of falls. In turn, falls can cause psychological issues such as the Fear of Falling (FOF), creating a destructive cycle (5).

Dynamic balance is considered the foundation for maintaining ambulation and performing functional activities. It depends on integrated input from the vestibular, proprioceptive, and visual systems, along with precise movement control by the central nervous system (6). Following a stroke, damage to the cerebral cortex or corticospinal tracts often results in symptoms such as muscle weakness, abnormal muscle tone (spasticity), decreased proprioception, loss of coordination, and muscular imbalance. Collectively, these impairments cause a significant decline in dynamic balance control (7). Therefore, enhancing dynamic balance ability has become a crucial goal in stroke rehabilitation.

SST refers to an active rehabilitation approach in which external support mechanisms such as slings or body-weight support devices are used to partially unload body weight while maintaining voluntary postural and locomotor control. The neurophysiological rationale for such partial-body-weight-support training lies in enhancing motor control and balance under reduced gravitational load, thereby facilitating adaptive neuromuscular responses during locomotor tasks (8). Body-weight-supported treadmills and related support systems have been widely used in neurological rehabilitation, including stroke, to enable safe task-specific gait practice and enhance motor input (9). Sling exercise training, as one form of SST, has shown improvements in balance and functional outcomes in stroke patients in randomized controlled studies and meta-analyses (10, 11). Accordingly, these intervention modalities are conceptualized as distinct technical implementations of a unified therapeutic construct—SST—characterized by externally supported, yet actively controlled, motor training. From a systematic review methodology perspective, complex interventions such as SST—composed of multiple interacting components—should be clearly defined to ensure meaningful synthesis (12).

A key benefit of SST is its ability to lower fall risk during rehab, reduce patient anxiety and fear, and enable patients who cannot walk independently to engage in rehabilitative activities and exercises (13, 14). The mechanism involved increased phosphorylation of protein kinase C in the affected brain region, which enhanced neuronal excitability and brain plasticity. This process also improved proprioceptive and dynamic sensory inputs, leading to gains in walking speed, stride length, endurance, and balance in stroke patients (15). Using suspension slings to support different body parts helps stroke patients move from an unstable to a more balanced state. This method not only strengthens trunk muscles and enhances motor function in the affected limbs but also improves the strength of core muscle groups and overall physical movement balance (16).

Despite the growing popularity of SST, a major limitation in current research is the lack of standardization and optimization of training dosage. Training dosage mainly includes intensity (such as the percentage of body weight support), frequency, session length, and overall intervention duration. Significant differences exist in dosage parameters across studies; for example, the body weight support ratio ranges from 30 to 70%, and the training duration varies from 2 to 12 weeks (9, 17). This variability makes it more difficult to determine the optimal dosage that yields the best therapeutic effect on dynamic balance. Incorrect dosing, whether too high or too low, can result in less effective or even harmful outcomes. For instance, supporting excessive body weight might reduce afferent signals from the ground, negatively affecting sensory input and muscle activation patterns (18). Therefore, systematically assessing the effects of SST dosage and finding its optimal range is essential for guiding clinical practice and maximizing rehabilitation benefits.

Since previous meta-analyses have rarely addressed this aspect, the present study aims to thoroughly evaluate the effectiveness of SST on dynamic balance in stroke patients through a systematic review and meta-analysis, with a focus on identifying the optimal training dosage. The study adhered to PRISMA guidelines and conducted a comprehensive search for relevant RCTs. Heterogeneity was evaluated using the I^2^ statistic and the Q-test. Additionally, the methods include publication bias detection, subgroup analysis, meta-regression, and Bayesian dose–response meta-analysis to model potential non-linear relationships between training dosage and dynamic balance outcomes, as well as the introduction of an exploratory extreme gradient boosting – SHAP machine learning approach to complement traditional meta-analytic regression. This comprehensive method allowed for a thorough assessment of SST’s impact on dynamic balance in stroke patients and helped identify the optimal implementation protocol.

## Materials and methods

### Study design

This article provides a systematic review of RCTs following the PRISMA guidelines (19). The protocol was registered with the International Prospective Register of Systematic Reviews (PROSPERO) under registration number CRD420251178724 before screening search results and was conducted in accordance with the PRISMA statement.

### Study inclusion criteria

The inclusion criteria for this study were as follows: ① RCT designs aimed at evaluating the effect of SST on the dynamic balance capacity in stroke patients; ② The intervention group received at least one systematic session of SST, while the control group received either no intervention or conventional training; ③ Study participants were limited to stroke patients, with no additional restrictions based on gender, age, ethnicity, or socioeconomic status; ④ Outcome measures were restricted to validated scales that assess dynamic balance or functional mobility with significant dynamic components. Specifically, the eligible instruments included the Berg Balance Scale (BBS), the Limits of Stability (LOS) test, and the Dynamic Gait Index (DGI). To ensure comparability, data were extracted from time points immediately following the intervention; ⑤ Publications had to be in English and have full text accessible. The exclusion criteria encompassed non-experimental studies, non-clinical research, secondary literature (such as systematic reviews and meta-analyses), non-original publications, and grey literature that had not been peer-reviewed. Although systematic reviews themselves were not eligible for inclusion, their reference lists were reviewed to identify potential primary studies.

### Search strategy

This study systematically searched the PubMed, Web of Science, PsycINFO, and Cochrane Library databases through 31 October 2025, to thoroughly gather RCTs examining the effects of SST on dynamic balance capacity in stroke patients. The search strategy was created using the PICOS framework, with stroke patients as the population (P), SST (at least one session) as the intervention (I), no intervention or conventional training as the comparison (C), changes in standardized balance scale scores as the primary outcome (O), and RCTs as the study design (S). Keywords and subject terms were combined; for example, TS = (sling exercise therapy OR suspension exercise training OR TRX OR sling training OR suspension training) AND TS = (stroke patient OR cerebrovascular accident patient OR brain attack patient OR acute cerebrovascular accident patient). The complete search strategies for each database are detailed in the Supplementary material (Retrieval strategy).

### Study selection process

The literature screening process was conducted thoroughly in accordance with PRISMA guidelines. All retrieved records were imported into Zotero 7.0 to automatically eliminate duplicates. Study screening employed a dual-blind method, where two independent reviewers first examined titles and abstracts to exclude studies that clearly did not meet the inclusion criteria. Full texts of studies that passed the initial screening were downloaded for further evaluation to ensure they aligned with the PICOS framework. Any disagreements between reviewers were resolved through consultation with a third reviewer to reach a consensus, thereby reducing selection bias. During the data extraction phase, a standardized form was used for dual independent entry, performed consecutively, with key items such as sample characteristics and intervention parameters cross-checked. Discrepancies in the data were resolved through discussion among a panel of three reviewers before reaching a final decision.

### Data synthesis

All statistical analyses were conducted in R 4.3.3, mainly using the packages meta, metafor, and ggplot2. For continuous outcome measures, the mean difference (MD) or the SMD was chosen as the effect size measure based on the study’s scales. Hedges’ g correction was applied, and effect sizes were interpreted as follows: g ≥ 0.8 indicates a significant effect, g ≥ 0.5 a medium effect, and g ≥ 0.2 a small effect (20). To ensure consistency and reproducibility, data synthesis was strictly based on end-of-treatment scores measured immediately post-intervention. In cases where a single study reported multiple eligible balance outcomes, the primary outcome as defined by the authors was prioritized. If no primary outcome was specified, the measure most frequently utilized across the included studies, namely the BBS, was selected to maximize homogeneity. Furthermore, the direction of effect sizes for all scales was standardized (refer to Supplementary File). Effect sizes were combined using inverse-variance weighting, and the primary analysis was performed with a REML random-effects model. When heterogeneity was low (I^2^ < 50%), it was considered that significant heterogeneity was not present (21). Adjustments were made using the Knapp-Hartung-Sidik-Jonkman (KHSJ) method, a robust approach chosen for its ability to provide more precise error estimation and confidence interval coverage in small-sample meta-analyses. Moreover, 95% prediction intervals (PIs) were calculated to estimate the range within which future study effect sizes may fall. Publication bias was assessed using funnel plots and Egger’s regression test. When the data appeared asymmetrical, the trim-and-fill method was applied to adjust the results accordingly (22). Outlying studies were identified using standardized residuals (|Z| > 2.5) and Cook’s distance (greater than three times the mean) (23). The sensitivity analysis was carried out using two strategies: (1) a leave-one-out analysis, performed by iteratively removing each study with the metainf function; and (2) a REML-based meta-regression to examine the relationship between moderating variables (such as intervention duration and patient type) and the effect size, with results visualized using bubble charts plots (24); subgroup stratification was performed to explore the sources of heterogeneity. To standardize data from different scales, the Hedges–Olkin formula was used to transform the original data: $\mathrm{SMD}=\frac{M_{\text{Intervention}}-M_{\text{Control}}}{{\mathrm{SD}}_{\text{Pooled}}},{\mathrm{SD}}_{\text{Pooled}}=\sqrt{\frac{(n_{1}-1){\mathrm{SD}}_{1}^{2}+(n_{2}-1){\mathrm{SD}}_{2}^{2}}{n_{1}+n_{2}-2}}\;$ (25). To evaluate the overall strength of the evidence, trial sequential analysis (TSA) was employed to determine the required information size (RIS/DIS). The RIS/DIS, along with the current cumulative information size and cumulative Z-values, was reported to assess the adequacy of the evidence (26). At the same time, a cumulative meta-analysis was performed by publication year. The cumulative SMD and its 95% confidence interval (CI) were repeatedly calculated to determine both when statistical significance was first achieved and the consistency of the effect estimate over time (27). Additionally, to enhance estimation accuracy in cases with small sample sizes or sparse data in specific exposure ranges, a Bayesian hierarchical dose–response model was used with restricted cubic splines. The model employed weakly informative priors and utilized Markov Chain Monte Carlo (MCMC) methods for parameter estimation (28, 29). An XGBoost algorithm was used to create a dose–response prediction model. Input features were preprocessed with one-hot encoding for categorical variables and standardization for continuous variables. The XGBoost–SHAP analysis was conducted as an exploratory, hypothesis-generating approach and was not intended for confirmatory inference or causal interpretation, particularly given the limited number of included studies (30, 31). The incremental value of XGBoost–SHAP lies in its capacity to flexibly explore potential non-linear patterns and interactions among multiple dose-related variables without imposing strong parametric assumptions (30, 32). Such exploratory insights complement conventional meta-regression and are particularly valuable when the functional form of the dose–response relationship is unknown (33). Hyperparameters were optimized using 3-fold cross-validation, with max_depth = 3 and eta = 0.05. The marginal contributions of variables to the SMD were measured with SHAP values: continuous variables were ranked by their mean absolute SHAP values. In contrast, categorical variables were assessed using the combined SHAP values of their dummy variables. The importance of features was ranked and visualized with bar plots, while non-linear relationships were displayed using beeswarm plots (30, 34). Model performance was assessed using the root mean square error and R^2^ metrics derived from cross-validation.

To reduce the risk of overinterpretation and overfitting, we prespecified the analytical framework, distinguishing confirmatory and exploratory inferences. Pooled effect estimates, subgroup analyses, and conventional meta-regression were treated as confirmatory analyses based on predefined hypotheses. In contrast, the XGBoost-SHAP analysis was prespecified as exploratory and was used solely for hypothesis generation and pattern exploration.

### Risk of bias (quality) assessment

The revised Cochrane Risk of Bias tool for Randomized Trials (ROB2) was used to evaluate the risk of bias in the included RCTs. This evaluation covered five key areas: (1) the randomization process; (2) deviations from the planned interventions; (3) missing outcome data; (4) how outcomes are measured; and (5) the selection of reported outcomes results (35). Two researchers independently evaluated the risk of bias using a three-tier classification system: ‘Low Risk’, ‘Some Concerns’, and ‘High Risk’. Disagreements in assessments were resolved through group discussion. When consensus could not be reached, the contested study was referred to a third researcher for arbitration.

## Results

### Study selection

**Figure 1:**
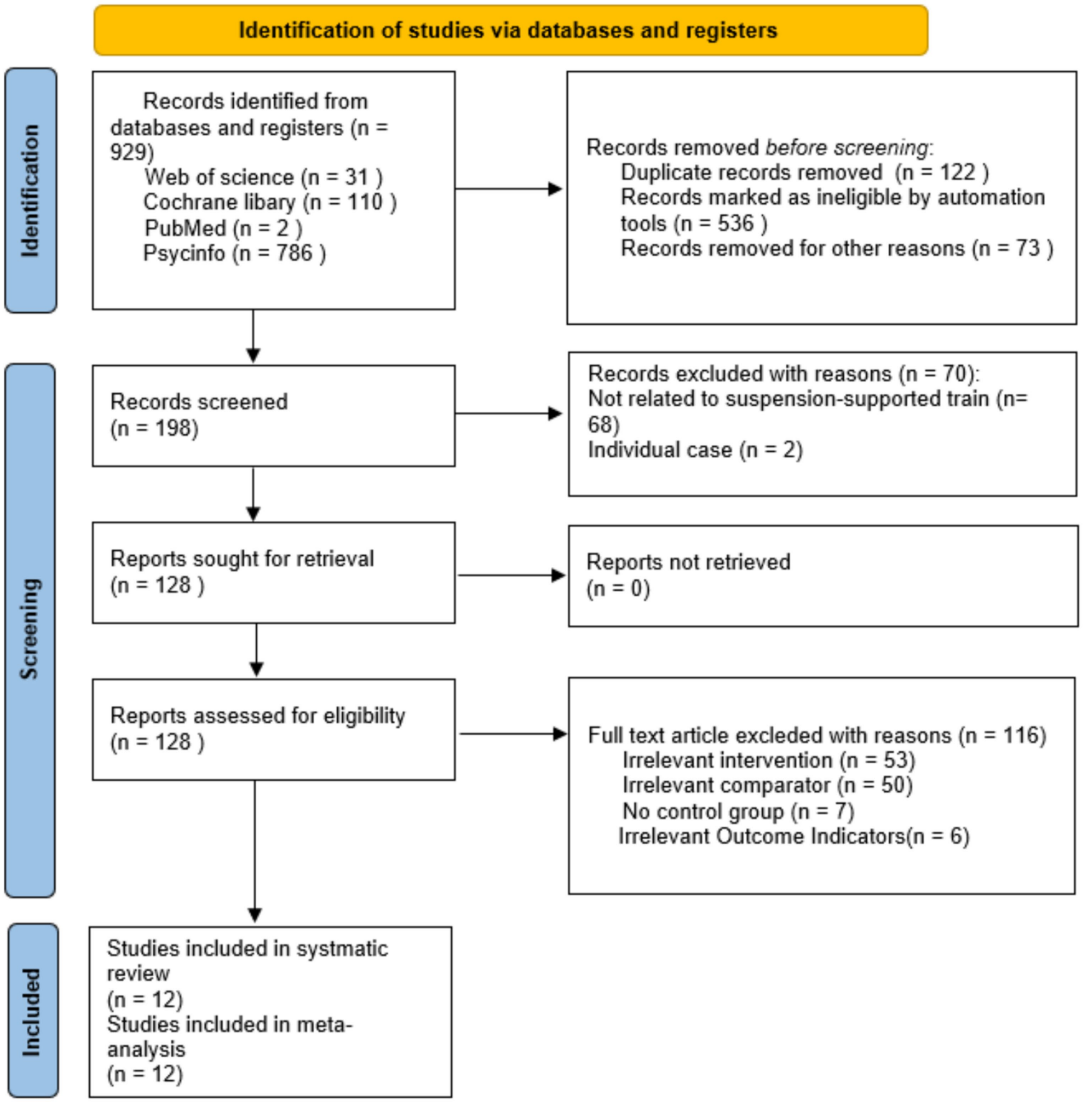
Flow diagram of the selection process.

The initial search across four databases yielded 929 records, of which 122 duplicates were identified and removed. The titles and abstracts of the remaining 807 records were screened, leading to the exclusion of 679 records that did not meet the inclusion criteria. The full texts of the remaining 128 citations were reviewed for eligibility. A total of 116 articles were excluded, with specific reasons detailed in the flow diagram. Ultimately, 12 studies were included in the systematic review, and all were incorporated into the meta-analysis (36–46) (Figure 1).

### Risk of bias of included studies

**Figure 2:**
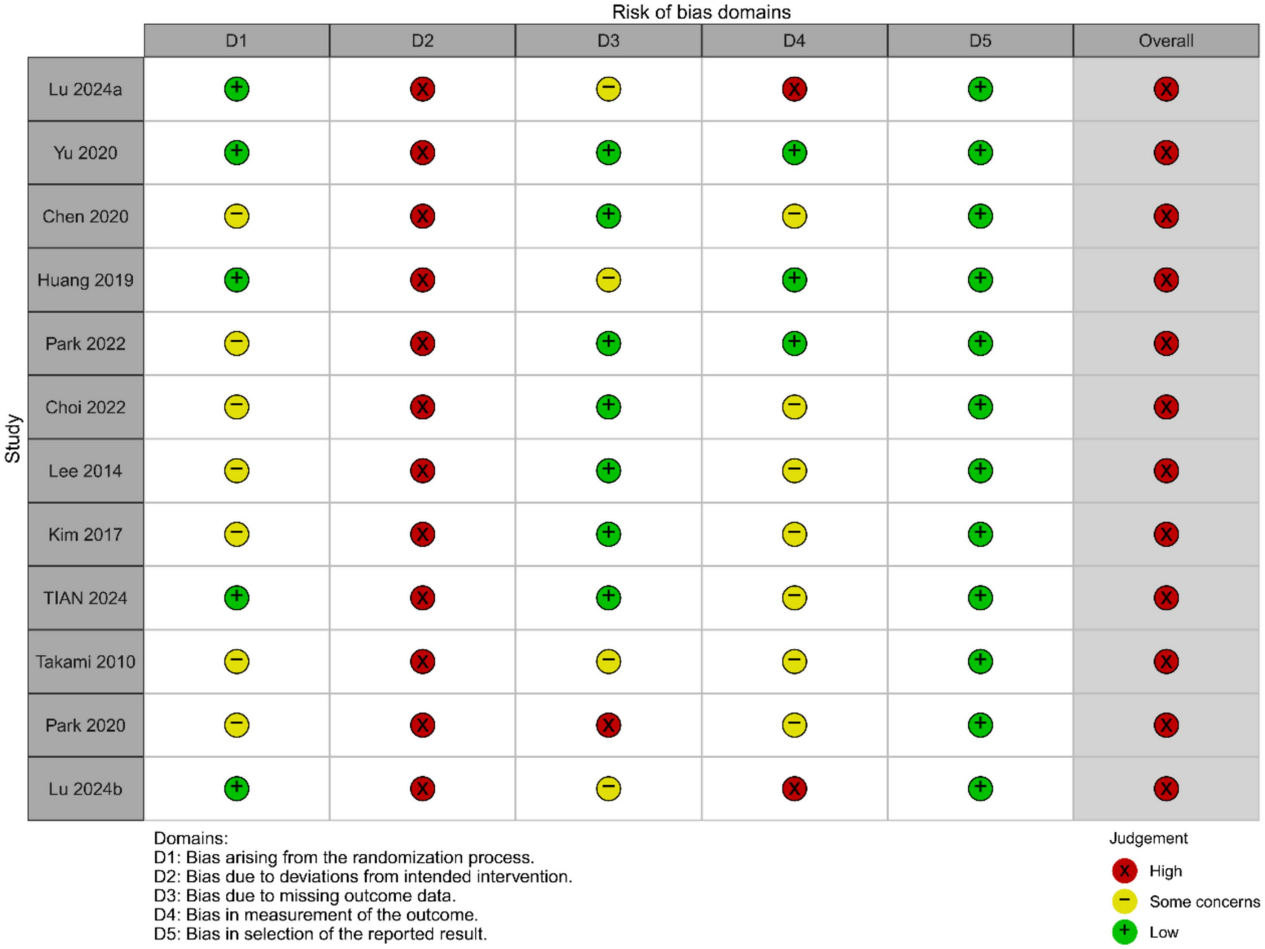
Risk of bias graph.

**Figure 3:**
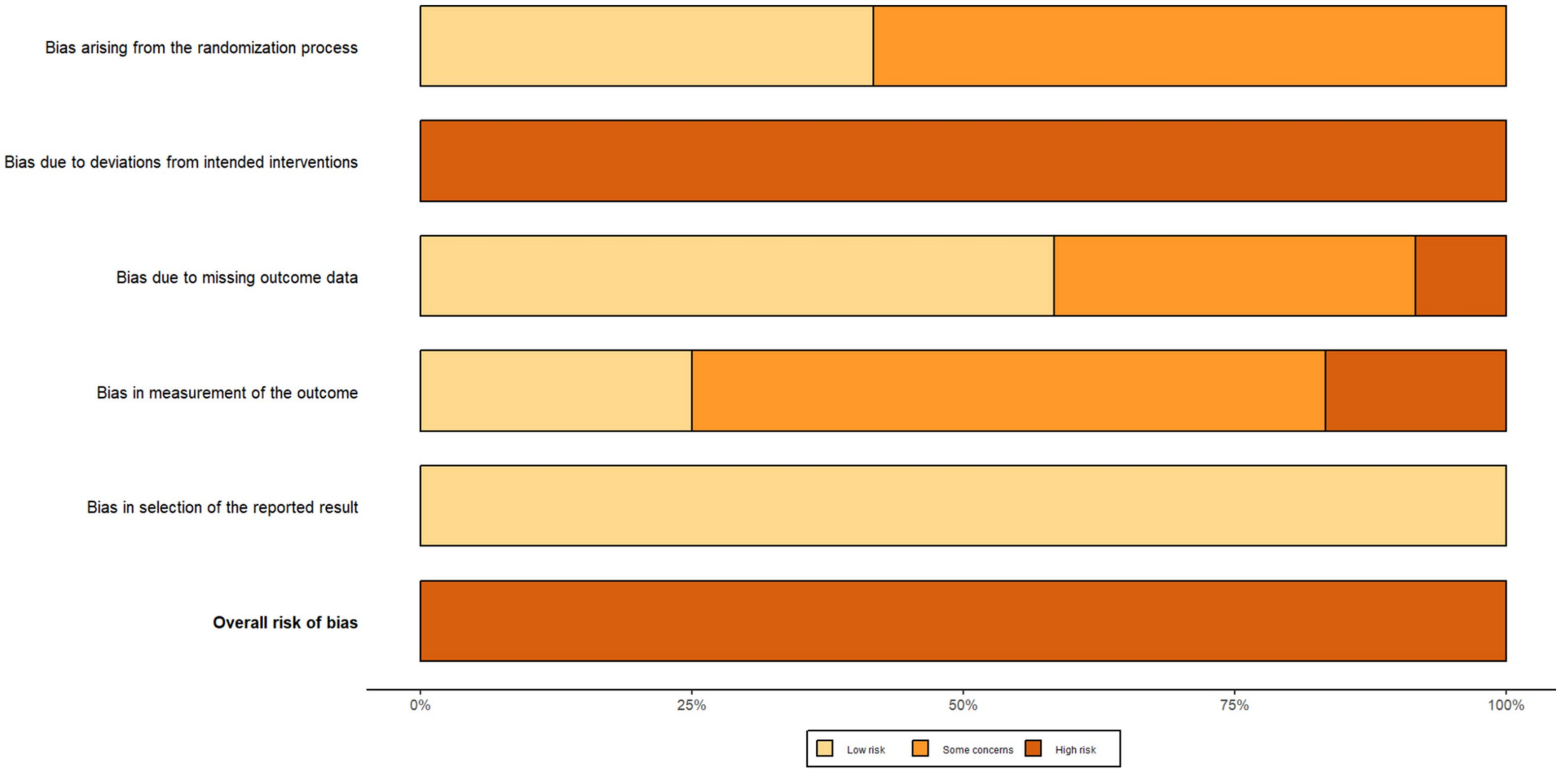
Risk of bias summary.

The results of the risk of bias assessment for the included studies are shown in Figures 2, 3 (red “X” = high risk, yellow “–” = Some concerns, green “+” = low risk). Two reviewers independently rated 12 RCTs based on the five domains of RoB 2.0 (D1 randomization process, D2 intervention deviation, D3 outcome missing data, D4 outcome measures, D5 report selection) on a case-by-case basis, with disputes discussed and decided by a third reviewer if necessary. The interdomain agreement was as follows: D1 (randomization process) simple agreement rate was 58.3% (7/12), Cohen’s *κ* = 0.250, weighted κ = 0.250 (interpreted as “fair” agreement); D2 (intervention deviation) simple agreement rate was 100% (12/12), Cohen’s *κ* = 1.000, weighted κ = 1.000 (interpreted as “perfect/perfect” agreement); D3 (outcome missing data) simple agreement rate 91.7% (11/12), Cohen’s *κ* = 0.840, weighted κ = 0.867 (interpreted as “perfect” agreement); D4 (outcome measure) simple agreement rate 83.3% (10/12), Cohen’s κ = 0.676, weighted κ = 0.707 (interpreted as “good” agreement); D5 (report selection) simple agreement rate 91.7% (11/12), Cohen’s κ = 0.000, weighted κ = 0.000 (“slight/slight” unanimous) (Detailed information can be found in the Supplementary File). Overall, most studies were rated as low risk in selective reporting (D5), suggesting that the transparency of the report was relatively reliable. However, there is a higher risk of “doubt” in the reporting of randomization information (D1) and outcome measures (D4), and several studies in the figure are presented as high risk in D4 and overall evaluation, suggesting that some studies have insufficient reporting details in terms of measurement methods and rater blinding. Missing outcome data (D3) showed a coexistence of low-risk, suspicious and high-risk, reflecting differences in follow-up completeness between studies. Based on the above bias distributions, we carefully interpret the systematic errors that may result from D1 and D4 in the main analysis and subgroup/dose–response analysis, and consider these patterns of bias as important factors in sensitivity analyses and evidence quality assessments (e.g., GRADE).

### Study characteristics

This meta-analysis ultimately included 12 RCTs (36–47), summarized in Table 1. The studies originated in countries including China, South Korea, Japan, and the United States. The study groups consisted of middle-aged to older stroke patients (ages 30–80 years), specifically including subgroups at various stages of the disease, such as chronic, subacute, and acute phases. Most interventions focused on SST, with some studies combining it with other rehabilitation methods such as Tai Chi, core muscle stability training, and gait training. The control groups received either standard therapy or other non-standard active treatments. Most studies involved an intervention frequency of 5 to 6 sessions per week, with a total duration of 2 to 12 weeks. Dynamic balance was assessed using tools such as the Berg Balance Scale, Limits of Stability, and the Dynamic Gait Index.

**Table 1:** Characteristics of the studies in the systematic review and meta–analysis.

Author/Year	Country	Design	Sample (T/C)	Age range	Subject type	Intervention (T/C)	Protocol	Tool
Lu, 2024a (36)	China	RCT	31/30	64.19 ± 6.38	Patients with Subacute stroke	Standard ST vs. Active control	60 min/session, 5×/week, 4 wk	BBS
Yu, 2020 (37)	China	RCT	35/36	30–75	Patients with Chronic stroke	BWS vs. Usual care control	40 min/session, 3×/week, 12 wk	BBS
Chen, 2020 (38)	China	RCT	90/90	59.09 ± 12.76	Patients with Subacute stroke	Standard ST vs. Active control	40 min/session, 6×/week, 8 wk	BBS
Huang, 2019 (39)	China	RCT	14/14	30–75	Patients with Chronic stroke	BWS vs. Usual care control	40 min/session, 3×/week, 12 wk	LOS
Park, 2022 (40)	Korea	RCT	19/19	72.4 ± 6.45	Patients with Subacute stroke	Standard ST vs. Active control	100 min/session, 5×/week, 8wk	BBS
Choi, 2022 (41)	Korea	RCT	6/6	57.05 ± 12.55	Patients with Chronic stroke	RAGT vs. Active control	150 min/session, 5×/week, 6 wk	BBS
Lee, 2014 (42)	Korea	RCT	10/10	62.95 ± 6.71	Patients with Chronic stroke	Standard ST vs. Usual care control	30 min/session, 3×/week, 4 wk	BBS
Kim, 2017 (43)	Korea	RCT	15/15	49.5 ± 14.78	Patients with Chronic stroke	BWS vs. Active control	30 min/session, 5×/week, 4 wk	DGI
TIAN, 2024 (44)	China	RCT	25/21	59 ± 11.5	Patients with Subacute stroke	RAGT vs. Usual care control	60 min/session, 10×/week, 2 wk	BBS
Takami, 2010 (45)	Japan	RCT	10/12	66.5 ± 8.45	Patients with Acute stroke	BWS vs. Usual care control	40 min/session, 6×/week, 3 wk	BBS
Park, 2020 (46)	USA	RCT	7/7	72.8 ± 9.9	Patients with Acute stroke	BWS vs. Active control	30 min/session, 2×/week, 7 wk	BBS
Lu 2024b (47)	China	RCT	31/31	50–80	Patients with Acute stroke	RAGT vs. Usual care control	20 min/session, 10×/week, 4 wk	BBS

T, intervention group; C, control group; Standard ST, traditional suspension training; BWS, body weight supported suspension training; RAGT, robot-assisted gait training; Usual care control, conventional therapy; Active control, other active interventions beyond conventional therapy; BBS, Berg Balance Scale; LOS, limits of stability; DGI, dynamic gait index, Lu 2024a (36) and Lu 2024b (47) represented separate publications authored by the same researcher within the same year.

### Meta–analysis

This study systematically evaluates the effect of SST on dynamic balance in stroke patients through a meta-analysis. A total of 12 eligible studies, involving 584 participants, were included. Due to significant heterogeneity among the studies (I^2^ = 76.6%, *p* < 0.0001), a random-effects model was used to calculate the overall effect sizes. The results demonstrate that SST significantly improves patients’ dynamic balance (SMD = 0.87, 95% CI: 0.49–1.26, *p* < 0.0001), highlighting its beneficial role in rehabilitation.

**Figure 4:**
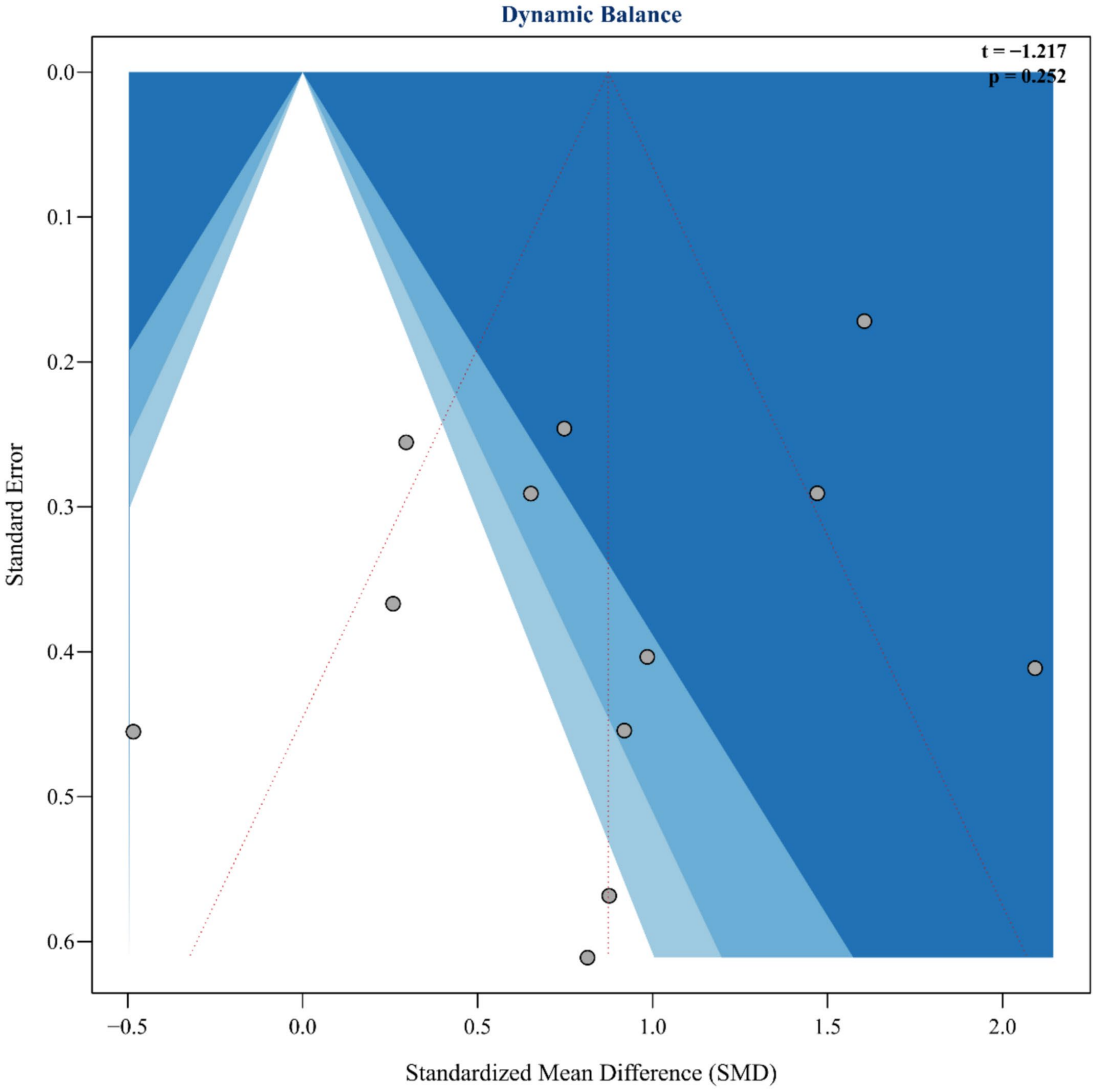
Funnel plot and Egger’s publication bias.

**Figure 5:**
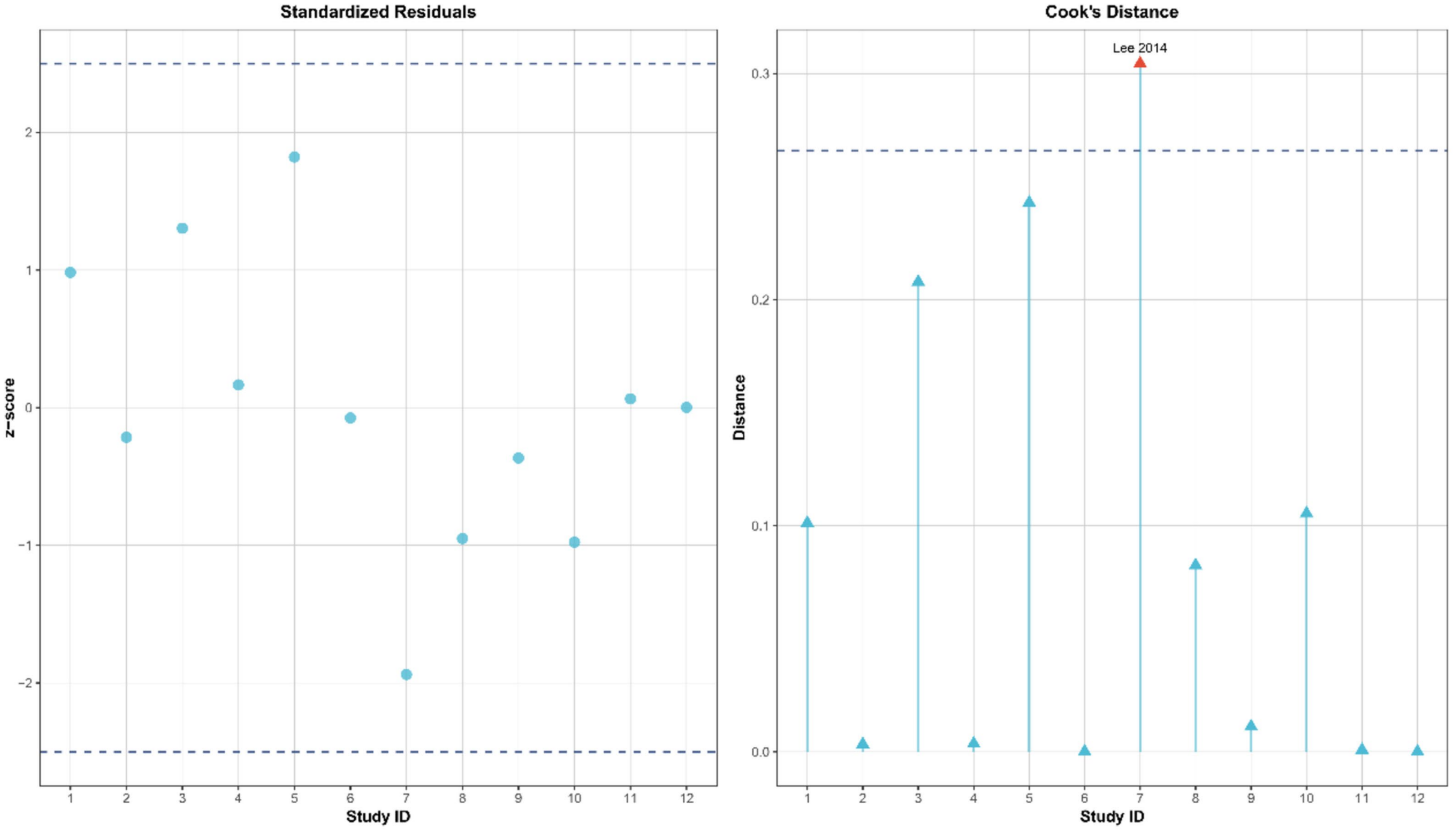
Influence diagnosis of included studies using standardized residuals and Cook’s distance.

To assess the robustness and reliability of the study results, this analysis systematically evaluated publication bias and influential studies following the guidelines of the Cochrane Handbook (v6.3). First, Egger’s regression test was used to identify potential small-study effects, showing no significant publication bias (*t* = −1.217, *p* = 0.252), as illustrated in Figure 4. Additionally, influential studies were identified using standardized residuals (|Z| > 2.5) and Cook’s distance (> 3 times the mean). Among these, the study by Lee et al. (42) exceeded the Cook’s distance threshold but did not qualify as an outlier based on standardized residuals; therefore, it was retained in the analysis (Figure 5). Sensitivity analysis revealed that heterogeneity (I^2^) ranged from 66.4 to 78.7% when studies were sequentially removed, with the overall findings remaining consistent (see Supplementary material for details). The trim-and-fill method suggested that two potentially missing studies were added to correct funnel plot asymmetry. After adjustment, the direction and importance of the original findings remained the same. This analysis indicates that although there may be some minor publication bias, it does not weaken the main conclusion that SST improves dynamic balance in stroke patients, thus confirming the robustness of the findings.

To evaluate the sufficiency of the evidence, a TSA was performed. The parameters were set as follows: a two-sided *α* of 0.05, a *β* of 0.20 (which corresponds to an 80% power), an expected effect size equal to the observed pooled standardized mean difference (SMD = 0.431), and an adjustment based on the observed heterogeneity (I^2^ = 0.7%). The analysis showed that the required information size (RIS) was 42.456. The current cumulative information size reached 143.607, which is 338.3% of the RIS. The cumulative Z value was 4.456, surpassing the typical two-sided critical value of 1.960. The information–time curve crossed the O’Brien–Fleming monitoring boundary (see Supplementary material for details). As a result, the available evidence was considered conclusive, and no further studies were deemed necessary.

Under the robust KHSJ random-effects model, sling training showed significant improvements in dynamic balance compared to the control group (SMD = 0.87, 95% CI: 0.44–1.30, *t* = 4.46, *p* < 0.0001), with a prediction interval from −0.48 to 2.23 (see Supplementary File).

**Figure 6:**
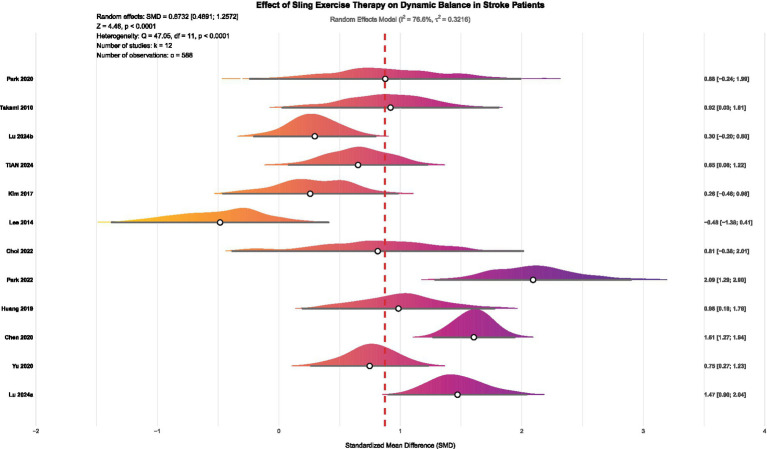
Forest plot of the effect of SST on dynamic balance in stroke patients. Lu 2024a (36) and Lu 2024b (47) represented separate publications authored by the same researcher within the same year.

In summary, this meta-analysis demonstrates that SST significantly enhances dynamic balance in stroke patients (SMD = 0.87, *p* < 0.0001; see Figure 6). Although there was notable heterogeneity among the studies, the main findings remained consistent after cross-verification using various methods, including Egger’s test, influence analysis, sensitivity analysis, and the trim-and-fill method, demonstrating strong reliability. Based on the GRADE assessment, moderate-certainty evidence indicates that suspension support training significantly improves dynamic balance in stroke patients. Although the certainty was downgraded by one level due to concerns regarding the randomization process and outcome measurement in several studies (D1, D4), extensive sensitivity and robustness analyses—including the KHSJ method, influence diagnostics, trim-and-fill procedures, and trial sequential analysis—suggest that heterogeneity was adequately addressed and did not warrant further downgrading; no additional concerns were identified for indirectness, imprecision, or publication bias (see Supplementary material for details).

### Subgroup analysis

This study used subgroup analysis to systematically identify key moderating factors that influence the effectiveness of SST in improving dynamic balance in stroke patients. The analysis examined the following predefined variables: ① patient subgroup characteristics; ② specific types of SST interventions; ③ countries or regions where the studies were conducted; ④ duration of each intervention session; ⑤ frequency of intervention delivery; ⑥ total length of the intervention period; and ⑦ assessment tools used to measure dynamic balance function. The results of all subgroup analyses were evaluated for evidence quality using the GRADE framework (48), thereby assessing the certainty of the findings. A comprehensive study examined the various influence pathways related to age. This research used linear and non-linear regression and the XGBoost machine learning algorithm to identify potential non-linear relationships and interaction effects between age and therapeutic effectiveness.

**Figure 7:**
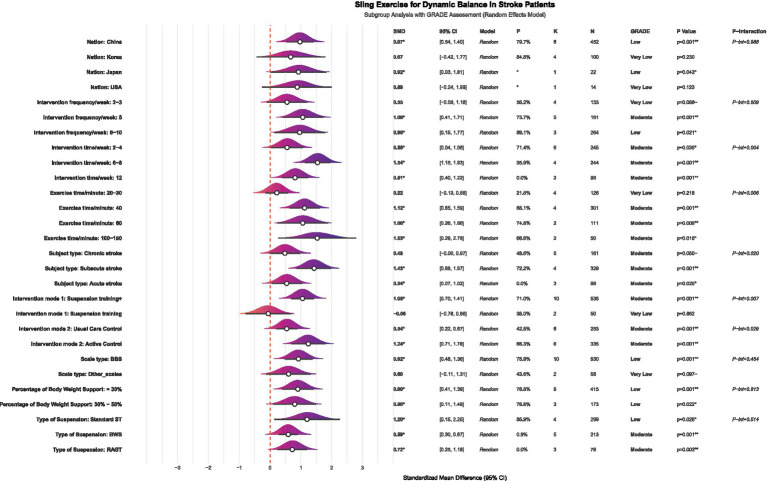
Forest plot of subgroup analysis on the effect of SST on dynamic balance capacity in stroke patients. K, number of studies; N, sample size; P-interaction, interaction tests; Suspension training, suspension-supported training alone; Suspension training +, combined treatment strategies of suspension-supported training with other interventions; Usual Care Control, conventional therapy; Active Control, other active interventions beyond conventional therapy; BBS, Berg Balance Scale; LOS, limits of stability; DGI, dynamic gait index, Standard ST, traditional suspension training; BWS, body weight supported suspension training; RAGT, robot-assisted gait training.

The subgroup analysis showed that several factors, including the duration of the intervention, the length of individual sessions, patient characteristics, and the intervention format, significantly affected the effectiveness of SST on dynamic balance in stroke patients (see Figure 7 for details). Significant differences in effectiveness were seen across different intervention durations (*p* = 0.004). Specifically, intervention periods of 2–4 weeks (*K* = 6, SMD = 0.55, *p* = 0.035, GRADE = Moderate), 6–8 weeks (*K* = 4, SMD = 1.54, *p* = 0.001, GRADE = Moderate), and 12 weeks (*K* = 2, SMD = 0.81, *p* = 0.001, GRADE = Moderate) showed more significant improvements. Regarding the length of individual sessions, durations of 40 min (*K* = 4, SMD = 1.12, *p* = 0.001, GRADE = Moderate), 60 min (*K* = 2, SMD = 1.06, *p* = 0.009, GRADE = Moderate), and 100–150 min (*K* = 2, SMD = 1.53, *p* = 0.016, GRADE = Moderate) all showed significant therapeutic benefits. SST has shown positive effects for both subacute stroke patients (*K* = 4, SMD = 1.43, *p* = 0.001, GRADE = Moderate) and acute stroke patients (*K* = 3, SMD = 0.54, *p* = 0.025, GRADE = Moderate). Regarding intervention formats, combined training (*K* = 10, SMD = 1.05, *p* = 0.001, GRADE = Moderate) particularly improved patients’ dynamic balance. Notably, the benefits of SST were clear regardless of whether the control group received conventional therapy (*K* = 6, SMD = 0.54, *p* = 0.001, GRADE = Moderate) or other active interventions (*K* = 6, SMD = 1.24, *p* = 0.001, GRADE = Moderate). Regarding the body weight support ratio, both 30 and 30–50% SST significantly improved dynamic balance in stroke patients, with the 30% body weight support ratio (*K* = 9, SMD = 0.90, *p* = 0.001, GRADE = Low) showing better effects compared to the 30–50% body weight support ratio (*K* = 3, SMD = 0.80, *p* = 0.022, GRADE = Low). Regarding suspension methods, traditional suspension showed the most effective intervention outcomes (*K* = 4, SMD = 1.20, *p* = 0.025, GRADE = Low), followed by robot-assisted gait training (*K* = 3, SMD = 0.72, *p* = 0.003, GRADE = Moderate). Although the effect of body weight support was smaller than that of the previous two methods, it remained statistically significant (*K* = 5, SMD = 0.59, *p* = 0.001, GRADE = Moderate). Given the limited number of studies, the results of the above subgroup analysis should be regarded as exploratory.

### Linear meta-regression

**Figure 8:**
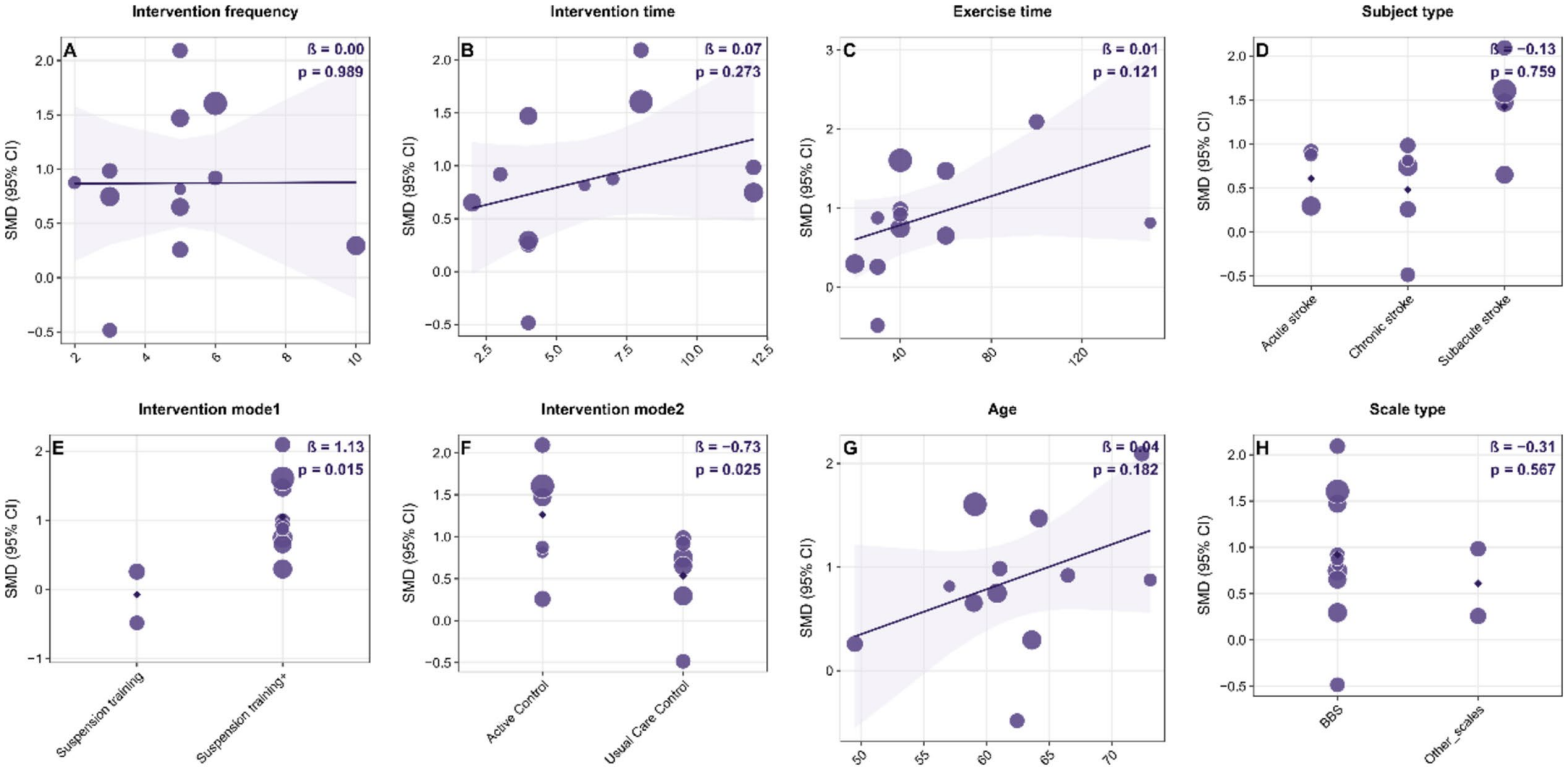
Bubble plots of study-level linear meta-regression exploring potential moderators of the effect of suspension-supported training (SST) on dynamic balance in stroke patients. Panels A–H show separate univariable models for: **(A)** intervention frequency (sessions/week); **(B)** intervention duration (weeks); **(C)** single-session exercise time (minutes); **(D)** subject type (acute, subacute, chronic stroke); **(E)** intervention mode 1 (SST alone vs. SST combined with other interventions); **(F)** intervention mode 2 / control type (usual care control vs. active control); **(G)** mean age (years); and **(H)** outcome scale type (BBS, LOS, DGI). In each panel, the x-axis represents the moderator and the y-axis represents the standardized mean difference (SMD). Bubble size is proportional to study weight. The solid line indicates the fitted meta-regression line. β denotes the regression coefficient for the moderator, and p indicates the significance level. SMD, standardized mean difference; CI, confidence interval; SST, suspension-supported training; BBS, Berg Balance Scale; LOS, Limits of Stability; DGI, Dynamic Gait Index.

Results from the multivariable linear regression analysis at the study level (Figure 8) revealed a significant link between intervention type and improvement in dynamic balance (*β* = 1.13, *p* = 0.015). Furthermore, the type of intervention used in the control group significantly affected the effect size (β = −0.73, *p* = 0.025). However, factors such as intervention frequency, total duration, single-session length, patient stroke type, age, and assessment tools did not have a significant impact (*p* > 0.05). It is important to recognize that this analysis was based on aggregated study-level data, and the direction of the SMD depends on how the variables are coded. Therefore, caution should be exercised when interpreting these moderating effects. Future research ought to validate these findings using individual participant data or larger randomized controlled trials.

### Nonlinear dose–response analysis

**Figure 9:**
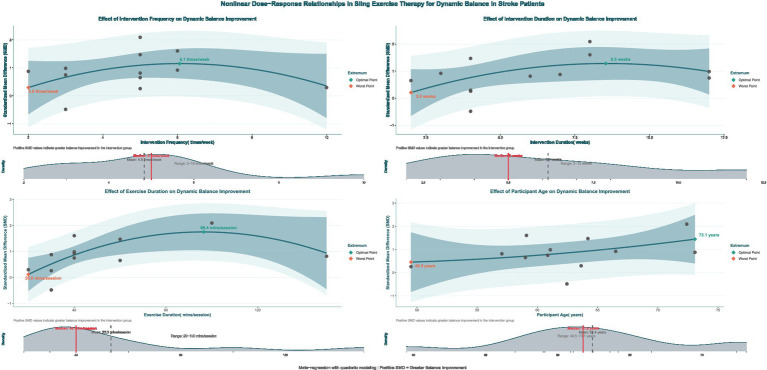
Non-linear regression plot of the effect of SST on dynamic balance capacity in stroke patients. The dark shaded area denotes the 95% confidence interval for the model–fitted curve (solid line). The light shaded area represents the 95% prediction interval, which reflects the expected range of individual observed values.

**Figure 10:**
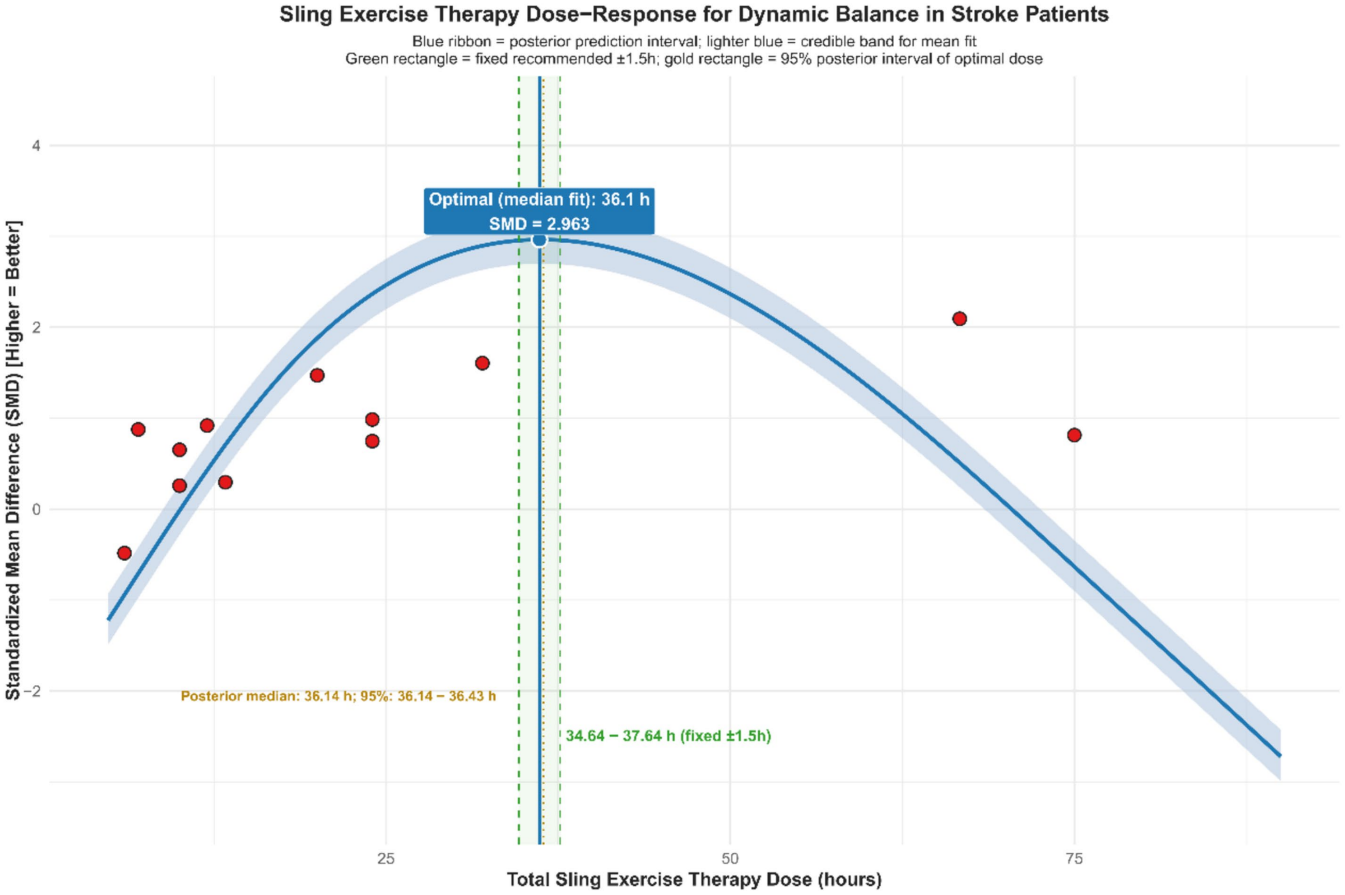
Dose–response relationship of SST on dynamic balance capacity in stroke patients.

Exploratory non-linear dose–response meta-regression analysis showed that improvements in dynamic balance function among stroke patients after SST follow a significant non-linear pattern. This relationship was defined by an optimal range where moderate doses and frequency produced better effects than extremes, with reduced intervention effects outside this range. By applying quadratic model fitting and sample density evaluation (Figure 9), the main findings were as follows: the optimal intervention frequency was estimated at about 6.0 sessions per week, whereas existing studies mostly used 5 sessions per week (median 4.8, roughly 5 on average). This suggests that increasing the frequency from the traditional 5 to approximately 6 sessions per week might yield better outcomes. The optimal intervention duration was estimated at about 8.5 weeks. However, most actual study protocols lasted around 6.2 weeks on average or 5 weeks as the median, suggesting that typical treatment courses might be shorter than the model’s predicted ideal length. The ideal single-session length was estimated at approximately 96.4 min, while the median and average durations reported in actual studies were about 40 min and 53.3 min, respectively. This indicates that typical session durations were shorter than the model’s recommended optimal length. According to the overall Bayesian dose–response curve (Figure 10), the maximum effect was achieved at a total intervention duration of approximately 36.1 h (corresponding to SMD ≈ 2.963), with a recommended range from 34.64 to 37.64 h. Based on these results, an evidence-based intermediate prescription protocol could be proposed: approximately 6 sessions per week, each lasting 90 to 100 min, over 8 to 9 weeks, with a total intervention time of 34.6 to 37.6 h, ideally around 36 h. We also reported the optimal dosing time (in hours) derived from posterior sampling, presented global density overlay plots (comparing the observed values yi with the density distributions of multiple yrep), displayed interval plots showing the posterior predictive intervals for each observation against the measured values, and provided detailed intervention parameters for each original study (see Supplementary material for details).

It is important to note that the recommended protocol was based on study-level aggregate data and non-randomized regression analyses. The results may have been influenced by different coding methods, uneven sample distribution, and variability between studies, with some optimal points located in areas with sparse sampling. Therefore, the protocol should be applied cautiously in clinical practice, taking into account each patient’s specific situation. Furthermore, future validation through larger or randomized controlled trials using individual participant data is expected.

### XGBoost machine learning exploratory analysis

**Figure 11:**
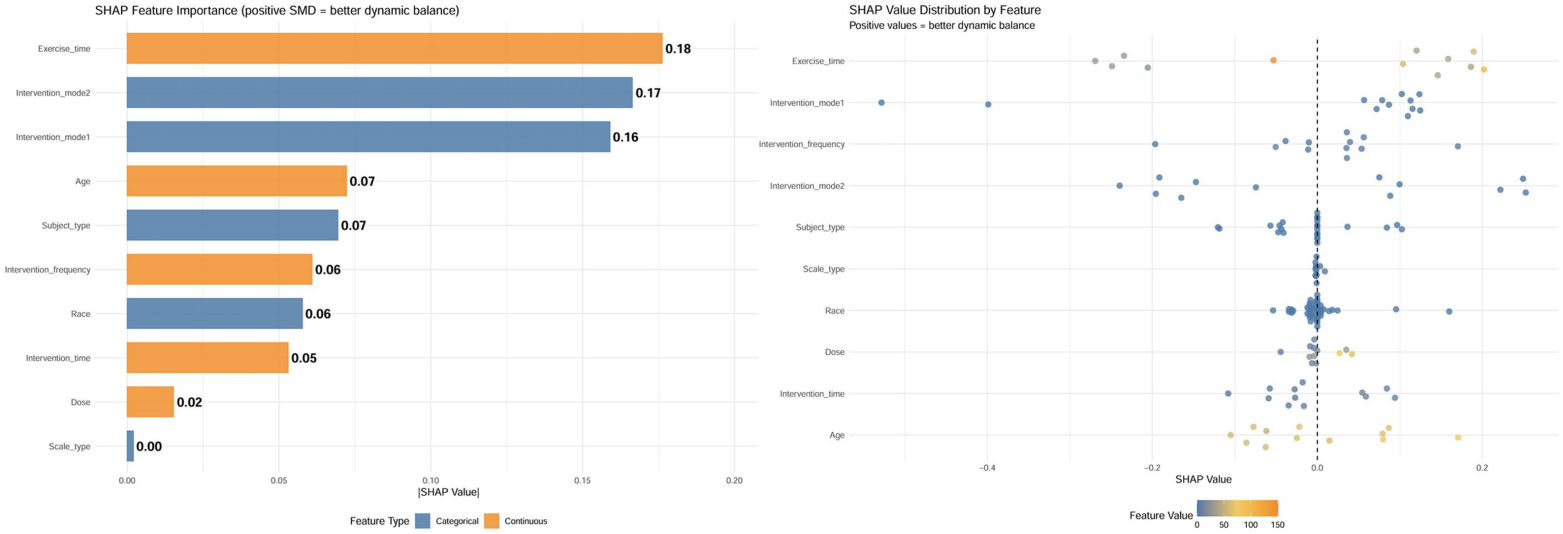
SHAP bar plot and beeswarm plot for the effect of SST on dynamic balance in stroke patients. Left: Mean absolute SHAP values, where higher values indicate greater feature influence on the model output, show that Exercise_time and Intervention_mode2 were the most influential predictors. Right: The SHAP swarm plot displays the distribution of each feature’s contribution to the prediction for every observation. A SHAP value greater than 0 (located to the right of the vertical dashed line) indicates that the feature value for that observation contributes positively to predicting a greater extent of improvement of dynamic balance ability, reflected by a positive standardized mean difference. The plot reveals non–linear and mixed effects across the dataset, with a limited number of outliers producing extreme SHAP values, which are further examined in the main text. Exercise_time, single session duration (minutes); Intervention_frequency, sessions per week; Intervention time, total intervention duration; Subject_type: Chronic stroke, Chronic stroke patients; Subacute stroke, subacute stroke patients; Acute stroke, acute stroke patients; Intervention mode 1: Suspension training+, combined treatment strategies of suspension-supported training with other interventions; Intervention mode 2; Usual care control, conventional therapy; Active control, other active interventions beyond conventional therapy.

Based on the SHAP analysis results shown in Figure 11 (left), exercise time and intervention modes (Intervention_mode1, Intervention_mode2) had the most significant impact on predicting improvements in dynamic balance function in stroke patients after SST, with mean absolute SHAP values ranging from 0.16 to 0.18. The influence of age, participant type, intervention frequency, ethnicity, and total intervention duration was less significant, with |SHAP| values ranging from 0.05 to 0.07. In contrast, the contribution of dosage was relatively minor, with |SHAP| values around 0.02. The assessment scale type had little predictive influence, as indicated by |SHAP| values near 0. The beeswarm plot (Figure 11, right) further demonstrated the directional effect and distribution patterns of each variable on the prediction outcome. The SHAP values for exercise time were generally positively skewed, indicating that longer single-session durations were consistently associated with improved dynamic balance performance. The presence of some lower or negative SHAP values in certain studies suggested potential influences, such as training fatigue or differences in protocol implementation. The distributions for the two intervention modes were fairly concentrated, indicating consistent effect directions. In contrast, the SHAP values for age and intervention frequency showed greater spread, suggesting heterogeneity across studies. Overall, the beeswarm plot results reaffirmed the importance of exercise time and intervention mode in the predictions, while also indicating that individual differences and specific implementation protocols may influence these variables. Relevant learning curves and key SHAP analysis results are provided in the Supplementary material. However, it is crucial to acknowledge that these feature importance rankings are derived from a machine learning model trained on a limited number of studies (*K* = 12). Therefore, these findings reflect statistical associations within the aggregated data rather than definitive causal mechanisms, and should be interpreted as hypothesis-generating insights subject to future verification.

## Summary

This meta-analysis showed that SST significantly improves dynamic balance in stroke patients (SMD = 0.87). Despite considerable heterogeneity across studies, the main conclusion remained solid after various checks, including Egger’s test, identification of high-impact studies, sensitivity analysis, and the trim-and-fill method. Trial sequential analysis also showed that the total information size exceeded the required sample size (information ratio = 12.07), and the cumulative Z-value crossed the standard significance boundary, supporting the study’s findings. Subgroup and dose–response analyses suggested that a higher-frequency intervention—about 6 sessions weekly, each lasting 90–100 min, over 8–9 weeks with a total of 34.6–37.6 h—may yield the most effective results. In the SHAP analysis, exercise duration and intervention mode were the most significant contributors to the prediction model, while negative or low SHAP values in some studies indicated potential confounding factors, such as training fatigue or variations in protocol implementation.

## Discussion

This study pooled 12 RCTs and showed that SST was associated with improvements in dynamic balance in stroke patients. In addition, exploratory subgroup analyses and dose–response modeling suggested that training parameters might have been related to the magnitude of the effect, but this still required further validation. The meta-analysis results of this study demonstrate that SST significantly improves dynamic balance in stroke patients (SMD = 0.87, 95% CI: 0.49–1.26, *p* < 0.0001). This finding emphasizes the positive impact of SST on neurological recovery and motor control reorganization. The effect size shows a large impact, demonstrating that SST is both statistically significant and practically beneficial in clinical settings. These findings support previous research indicating that SST effectively enhances core muscle stability in stroke patients, resulting in improved balance and gait control (38, 49). Furthermore, Huang et al. (39) found that Tai Chi gait training, when combined with suspension support, significantly reduces the risk of falls among patients. SST, by providing sling support and a controllable unstable support environment, allowed patients to perform more task-related balance exercises under relatively safe and controlled loading conditions, thereby facilitating the establishment and consolidation of dynamic postural control strategies. At the same time, SST might have enhanced sustained contraction of the core and pelvic muscles, improved control of the center of mass, and augmented vestibular or proprioceptive input and integration within the axial kinetic chain, thereby improving dynamic balance ability in stroke patients at the clinical level (50, 51).

Although multiple robustness tests indicated that the pooled effect was directionally consistent, heterogeneity across studies remained high (I^2^ = 76.6%). Although multiple verification approaches—including sensitivity analysis, publication bias tests, the trim-and-fill method, and trial sequential analysis—showed that the direction and significance of the results remained consistent, the included trials differed substantially in disease stage, training parameters, and combined interventions, suggesting that the generalizability and certainty of the evidence were still limited. This consistency demonstrates the robustness and reliability of the findings. Therefore, SST was generally likely to help improve dynamic balance function in stroke patients, but the specific magnitude of benefit might still have varied across populations and intervention protocols (37, 38, 49).

Subgroup analysis also revealed that the effectiveness of SST was affected by several factors, including the duration of the intervention, the length of individual sessions, the stage of the patient’s disease, and the format of the intervention. Specifically, intervention protocols lasting 6–8 weeks with session durations of 40–100 min were likely to yield the most favorable outcomes (SMD range: 0.81–1.54). This suggests that moderate-intensity training over moderate periods might be more conducive to the development of neural plasticity and the consolidation of motor control skills (52). Combined intervention models, such as SST integrated with Tai Chi gait exercises or core stability training, have demonstrated more significant effects (SMD = 1.05). This suggests that suspension systems could serve as multimodal rehabilitation platforms, producing synergistic effects when used with conventional exercise therapies (37). Regarding patient subtypes, subacute stroke patients gained the most benefit from SST (SMD = 1.43), followed by acute stroke patients (SMD = 0.54). This suggests that SST can enhance rehabilitation outcomes when used during the recovery-sensitive period (53). Additionally, the body weight support ratio influences therapeutic outcomes, with around 30% support offering the optimal effect. This level likely reduces load effectively while preserving enough muscle activation (54). The interventional effects of SST were found to be highly context-dependent, requiring optimization of parameters based on the patient’s disease stage and individual circumstances capabilities (55). However, it should be noted that the number of studies included within each subgroup was limited, and different disease stages were often accompanied by differences in baseline functional levels, routine rehabilitation content, and treatment adherence. Therefore, these subgroup findings were more appropriate for explaining heterogeneity and generating hypotheses, rather than serving as direct prescriptive conclusions.

This study further analyzed and identified the optimal prescription protocol for SST using regression analysis and an XGBoost model. The non-linear regression results showed threshold effects for intervention frequency, duration, and single-session length: the greatest improvement in dynamic balance (SMD ≈ 2.96) was maybe observed with about six sessions per week, a duration of eight to nine weeks, a single session lasting roughly ninety to one hundred minutes, and a total intervention dose of approximately thirty-six hours (ranging from thirty-four point six to thirty-seven point six hours). A slight decline in effectiveness was observed beyond these thresholds, suggesting that excessive training might cause fatigue or diminished performance adherence (56). Cabanas-Valdés et al. (57), in their study of the effects of core stability training on balance recovery in stroke patients, found that higher training frequencies did not provide additional therapeutic benefits. Instead, they discovered that fatigue and reduced attention hindered gains in neuromuscular function control. The machine learning model XGBoost-SHAP further confirmed this trend, showing that training duration and intervention mode are the primary factors influencing therapeutic outcomes. It should be emphasized that the above optima and thresholds were derived from study-level aggregated data modeling, with sparse data-point distributions in some ranges, and that model outputs (e.g., predicted SMD peaks) should not have been equated with the overall pooled effect. Therefore, these findings were better interpreted as “target parameter ranges” for hypothesis generation rather than as definitive clinical prescriptions. At the same time, dose modeling at the study level might have been subject to ecological fallacy, so between-study associations did not necessarily represent true dose–response relationships at the individual level. Based on the current evidence, it was recommended that, under the premise of patient tolerance and adequate safety monitoring, training parameters should preferentially refer to moderate-dose ranges and be individually adjusted according to disease stage, baseline function, and concomitant rehabilitation content.

The findings of this study have important implications for stroke rehabilitation. On one hand, this research offers an evidence-based foundation for creating SST protocols, which can assist rehabilitation centers in designing personalized balance training programs. On the other hand, the results support including SST as a key component of comprehensive rehabilitation programs, suggesting that combining it with core stability exercises, gait training, or virtual reality technology could further enhance therapeutic outcomes.

## Conclusion

The results of this meta-analysis suggested that SST was likely to improve dynamic balance function in stroke patients overall. Exploratory dose–response and machine learning analyses indicated that a combination of moderately high frequency and moderate duration/cycle might have been associated with larger effects (for example, approximately 6 sessions per week, about 90–100 min per session, over roughly 8–9 weeks). In clinical practice, parameters should be individualized with safety monitoring and patient tolerability in mind, particularly with consideration of disease stage, baseline function, and concomitant rehabilitation content. The XGBoost–SHAP analysis in this study further revealed the influence of factors, such as single-session training duration and intervention mode, on treatment efficacy, providing data-driven inferential insights into outcome heterogeneity and into the development of individualized rehabilitation programs. However, given the considerable heterogeneity between studies, the presence of risk of bias and protocol differences, and the fact that the dose–response and machine learning analyses were based on study-level data modeling, the frequency, duration, and cycle parameters proposed in this study should have been regarded as exploratory, hypothesis-generating “preliminary target ranges,” rather than definitive clinical prescriptions. Future research still needed adequately powered, well-reported RCTs with longer follow-up and more standardized intervention protocols (ideally combined with individual participant data analyses when necessary) to verify the optimal dose and its long-term efficacy and safety.

### Limitations

This study has several limitations. First, there was considerable variability among the included studies regarding participant characteristics, such as disease stage, age, and level of functional impairment, as well as differences in SST protocols, including body weight support ratio, training frequency, and session length. This variability may have affected the generalizability and external validity of the findings. Additionally, the meta-regression and dose–response analyses were conducted using aggregated study-level data, which prevented examining how individual patient characteristics influenced treatment response and limited the ability to control for potential confounding factors at the individual level. Third, some included studies had small sample sizes and insufficient reporting of methodological quality, particularly regarding the implementation of blinding and allocation concealment, which introduces a high risk of bias and could compromise internal validity. Fourth, the follow-up periods in most randomized controlled trials were relatively brief, limiting the ability to assess the long-term effectiveness of SST. Finally, although introducing the XGBoost-SHAP machine learning approach was innovative for analyzing influencing factors and dose–response relationships, its results were constrained by the feature distribution and the sample size of the current data. The model’s predictive ability and generalizability still require validation through future independent studies. These limitations suggest that the current conclusions should be interpreted with caution and emphasize the need for future rigorous, long-term follow-up and high-quality studies based on individual participant data.

## Data Availability

The original contributions presented in the study are included in the article/Supplementary material, further inquiries can be directed to the corresponding author.
