# Supplementary material for: The effects of suspension-supported training on dynamic balance capacity in stroke patients: a systematic review and meta-analysis enhanced by XGBoost machine learning
Source: Front Med (Lausanne). 2026 Feb 9;13:1747067. doi: 10.3389/fmed.2026.1747067 (PMC12926393; doi:10.3389/fmed.2026.1747067)
Supplement: Supplementary file 2 [file Table_2.DOCX]

**1.Lu, C. (2024)**

**D1 — Bias arising from the randomization process.**

**R1 (Low risk):** In the graph, this study is rated as a green "+" in this domain, which means low risk. The reviewer agreed that their randomization processes, such as sequence generation and assignment hiding, were adequate.

**R2 (Low risk):** Agree with R1's evaluation. Based on the graph results, the study was considered to be at low risk of bias during the randomization process.

**Consensus: Low risk**

**D2 — Bias due to deviations from intended interventions.**

**R1 (High risk):** In the chart, this study is rated as a red "×" in this domain, indicating high risk. This suggests that there is a significant risk of bias in the delivery of interventions, which may stem from a lack of blinding of participants and therapists, and that studies do not adequately address or analyse the resulting biases, or that appropriate analytical strategies (e.g., failure to adhere to intention-to-treat analysis) are not used to estimate the effects of allocating interventions.

**R2 (High risk):** Agree with R1. The graph results show a high risk, indicating significant deficiencies in intervention implementation and effect evaluation, posing a serious threat to the authenticity of estimated effects.

**Consensus: High risk**

**D3 — Bias due to missing outcome data.**

**R1 (Some concerns):** The evaluation of this study in this domain in the chart is yellow "-", representing some concerns. This suggests that there may be a risk of bias associated with missing data, such as unbalanced loss to follow-up rates, inappropriate methods for handling missing data, or failure to perform relevant sensitivity analyses.

**R2 (Some concerns):** Agree with R1. The chart rating as "partially concerning" means that the study has certain flaws in the integrity of outcome data and introduces a degree of uncertainty.

**Consensus: Some concerns**

**D4 — Bias in measurement of the outcome.**

**R1 (High risk):** In the chart, this study is rated as a red "×" in this domain, indicating high risk. This strongly suggests that measures of outcome measures, especially subjectivity measures, are likely to be significantly affected by knowledge of treatment groups, such as assessor unblinding, and that this measure bias has a substantial impact on effect estimates.

**R2 (High risk):** Agree with R1. High-risk assessments mean that bias in outcome measures is likely to have occurred and seriously undermines the confidence of the findings.

**Consensus: High risk**

**D5 — Bias in selection of the reported result.**

**R1 (Low risk):** In the graph, this study is rated as a green "+" in this domain, which means low risk. This suggests that the risk of selection bias in the reporting of study results is low, possibly because the study has a prospective, publicly accessible trial protocol and the reported results are consistent with the analysis plan.

**R2 (Low risk):** Agree with R1. The low-risk assessment means that we have a high level of confidence that the study reported all pre-defined assays.

**Consensus: Low risk**

**2.Yu et al. (2020)**

**D1 — Bias arising from the randomization process.**

**R1 (Low risk):**​ The paper explicitly states that randomization was performed using "a sealed envelope containing a random allocation sequence number" generated by "an independent statistician using Excel." This describes an adequate sequence generation method. Allocation concealment is also addressed through the use of sealed envelopes.

**R2 (Low risk):**​ I agree with R1. The methods for both sequence generation and allocation concealment are clearly described and are considered low risk. The baseline characteristics table (Table 1) confirms that the groups were comparable at baseline.

**Consensus: Low risk**

**D2 — Bias due to deviations from the intended interventions.**

**R1 (High risk):**​ The interventions (conventional rehab vs. conventional rehab plus BWS-Tai Chi) are physical and behavioral, making blinding of participants and therapists impossible. This creates a high risk of performance bias. The analysis is described as intention-to-treat (ITT), and the flow diagram (Figure 1) shows no post-randomization exclusions, which is a strength. However, the high risk of bias due to the non-blinded nature of the intervention is a significant concern for this domain.

**R2 (High risk):**​ I concur. The nature of the intervention inherently prevents blinding. While ITT analysis was used, the potential for deviations from the intended intervention due to participants' and therapists' awareness of the group assignment is substantial and cannot be mitigated.

**Consensus: High risk**

**D3 — Bias due to missing outcome data.**

**R1 (Low risk):**​ The CONSORT flow diagram (Figure 1) indicates that all 71 randomized participants (BWS-TC=35, Control=36) were included in the analysis. The paper states that "All evaluations were performed before and 12 weeks after intervention" and that analysis was on an ITT basis. There is no indication of missing outcome data.

**R2 (Low risk):**​ Agree with R1. The flow diagram and the consistent group numbers in the results tables demonstrate complete outcome data for all randomized participants. The risk of bias from missing data is low.

**Consensus: Low risk**

**D4 — Bias in measurement of the outcome.**

**R1 (Low risk):**​ The paper states that this was an "assessor-blinded" trial. It specifically mentions that "The statistician, outcome assessors, and data analyzers were blinded to study recruitment, intervention, and evaluation." Blinding of outcome assessors is a key strength for reducing detection bias, especially for the primary outcome (Limits-of-Stability test) and secondary outcomes like BBS and FMA.

**R2 (Some concerns):**​ While the paper claims assessor blinding, the primary outcome (Limits-of-Stability test) requires active participant performance. An assessor watching a participant perform Tai Chi footwork, even with BWS, might deduce the group assignment, potentially introducing bias. This introduces some uncertainty.

**R3 (Low risk):**​ I side with R1. The manuscript explicitly describes blinding of assessors. The concern raised by R2, while theoretically possible, is speculative. There is no evidence in the text to suggest the blinding was broken. The described methodology is robust for this domain.

**Final Consensus: Low risk**

**D5 — Bias in selection of the reported result.**

**R1 (Low risk):**​ The trial was prospectively registered on ClinicalTrials.gov (ChiCTR1900020758). The reported outcomes (Limits-of-Stability, gait parameters, FMA, BBS) appear consistent with what would be expected for this type of intervention. The presence of a protocol reduces the risk of selective outcome reporting.

**R2 (Low risk):**​ I agree. The pre-registration provides a clear protocol against which the reported results can be compared. There is no indication of selective reporting of outcomes or analyses.

**Consensus: Low risk**

**3.Chen et al. (2020)**

**D1 — Bias arising from the randomization process.**

**R1 (Some concerns):**​ The paper states that "The 180 included patients were randomly divided into an observation group and a control group." However, it provides no details on the method used for random sequence generation (e.g., computer-generated, random number table) or on the measures taken for allocation concealment (e.g., sealed envelopes). The baseline table (Table I) shows comparability, but the lack of methodological detail introduces uncertainty.

**R2 (Low risk):**​ I understand R1's concern, but I have a more optimistic interpretation. The baseline table demonstrates successful randomization with no significant differences in all measured characteristics. In the absence of evidence to the contrary, and given the clear group balance, I judge the risk to be low. The lack of detail is a reporting issue.

**R3 (Some concerns):**​ I am called upon as R1 and R2 disagree. I side with R1's more cautious approach. The CONSORT statement emphasizes the importance of explicitly describing the methods of both sequence generation and allocation concealment. Their absence means we cannot rule out the possibility of a non-random approach. The baseline comparability is reassuring but does not fully mitigate this uncertainty.

**Final Consensus: Some concerns**

**D2 — Bias due to deviations from the intended interventions.**

**R1 (High risk):**​ The interventions (core muscle stability training + conventional therapy vs. trunk control training + conventional therapy) are distinct physical therapies. Blinding of participants and therapists was impossible. The analysis does not explicitly state an intention-to-treat (ITT) principle. The paper states that "Both groups successfully completed this study," suggesting no dropouts and a per-protocol analysis. The high risk of performance bias due to the non-blinded design is a significant concern.

**R2 (High risk):**​ I concur. The nature of the interventions makes blinding of participants and personnel unfeasible. Knowledge of the assigned group could have influenced the delivery of therapy and participant effort.

**Consensus: High risk**

**D3 — Bias due to missing outcome data.**

**R1 (Low risk):**​ The paper explicitly states that "Both groups successfully completed this study." The results are presented for all 90 participants in each group. There is no mention of attrition or missing data.

**R2 (Low risk):**​ I agree. The report indicates 100% completion and data availability for all randomized subjects.

**Consensus: Low risk**

**D4 — Bias in measurement of the outcome. (Revised)**

**R1 (Some concerns):**​ Upon re-evaluation, I revise my initial judgment. The paper states: "All tests were conducted in a single-blinded manner, i.e. the operators were unaware of study grouping or treatment methods." This is a strength. However, the outcomes are a mixture of types. The musculoskeletal ultrasonography measurements are highly objective and instrument-based, and the risk of bias for these is low. The primary clinical scales (Berg Balance Scale, Brunnstrom staging, Fugl-Meyer Assessment) involve clinical judgment. While the assessors were blinded, the participants were not. For performance-based outcomes like the BBS and FMA, the participants' knowledge of their group assignment could influence their performance (e.g., effort, confidence) in a way that might be detectable to the assessor, potentially unblinding them indirectly. This introduces some uncertainty. Therefore, while the risk is lower than if the assessors were explicitly unblinded, it is not negligible.

**R2 (Some concerns):**​ I agree with R1's revised assessment. This is a nuanced point. The blinding of the assessor is crucial, but for functional performance tests, the participant's performance itself can be a source of indirect unblinding. A participant in the experimental group, knowing they received a novel core training, might perform with more confidence or effort, which could subtly influence the way they perform tasks on the BBS, potentially providing cues to the assessor. This is a potential limitation that prevents us from being fully confident that the measurement of these key clinical outcomes was not biased.

**Final Consensus: Some concerns**

**D5 — Bias in selection of the reported result.**

**R1 (Low risk):**​ The study reports a standard set of outcomes for a core stability trial (BBS, Brunnstrom, FMA, 10m walk test, muscle thickness). All outcomes mentioned in the "Evaluation Indices" section are fully reported in the Results section (Tables II, III, IV). There is no indication of selective reporting.

**R2 (Low risk):**​ I agree. The outcomes are clinically relevant and pre-specified. The results are presented consistently.

**Consensus: Low risk**

**4.Huang et al. (2019)**

**D1 — Bias arising from the randomization process.**

**R1 (Low risk):**​ The paper explicitly states that randomization was performed using "a sealed envelope containing a random allocation sequence number" generated by "an independent statistician using Excel." This describes an adequate sequence generation method. Allocation concealment is also addressed through the use of sealed envelopes.

**R2 (Low risk):**​ I agree with R1. The methods for both sequence generation and allocation concealment are clearly described and are considered low risk. The baseline characteristics table (Table 1) confirms that the groups were comparable.

**Consensus: Low risk**

**D2 — Bias due to deviations from the intended interventions.**

**R1 (High risk):**​ The interventions (conventional rehab vs. conventional rehab plus BWS-Tai Chi) are physical and behavioral, making blinding of participants and therapists impossible. This creates a high risk of performance bias. The analysis is described as intention-to-treat (ITT), and the flow diagram (Fig. 1) shows some post-randomization exclusions and dropouts, but the analysis appears to include all randomized participants (n=14 per group in results tables against 28 randomized). However, the high risk of bias due to the non-blinded nature of the intervention is a significant concern for this domain.

**R2 (High risk):**​ I concur. The nature of the intervention inherently prevents blinding. While ITT analysis is mentioned, the potential for deviations from the intended intervention due to participants' and therapists' awareness of the group assignment is substantial and cannot be mitigated.

**Consensus: High risk**

**D3 — Bias due to missing outcome data.**

**R1 (Some concerns):**​ The CONSORT flow diagram indicates that of the 28 randomized participants, 3 withdrew after allocation (1 transferred, 2 lost interest), leaving 25 for analysis. This represents an attrition rate of approximately 11%. The paper states analysis was ITT but does not describe the specific method for handling the missing data from these 3 participants (e.g., last observation carried forward, multiple imputation). This introduces uncertainty.

**R2 (Low risk):**​ I understand R1's concern. However, the attrition rate, while above 10%, is relatively low (3/28). More importantly, the paper explicitly states an intention-to-treat analysis was used. In the absence of evidence to the contrary, we should assume a valid ITT approach was applied, which is the gold standard for handling missing data in RCTs and aims to preserve the baseline randomization. Therefore, I judge the risk to be low.

**R3 (Some concerns):**​ I am called upon as R1 and R2 disagree. I side with R1's more cautious approach. While the use of ITT is a positive signal, the CONSORT guidelines emphasize the importance of specifying *how*ITT was implemented, especially when there are post-randomization exclusions or dropouts. The lack of detail on the specific method (e.g., was a simple baseline observation carried forward used for the 3 dropouts?) means we cannot be certain that the analysis fully addressed the potential bias introduced by the missing data. A small degree of uncertainty remains.

**Final Consensus: Some concerns**

**D4 — Bias in measurement of the outcome.**

**R1 (Low risk):**​ The paper clearly states this was an "assessor-blinded" trial. It specifies that "The statistician was not involved in the study recruitment, intervention, or evaluation. Group allocations and interventions were not disclosed to the outcome evaluators and data analysers." Blinding of outcome assessors is a key strength for reducing detection bias for the primary and secondary outcomes.

**R2 (Low risk):**​ The description of blinding is clear and specific. The assessors measuring the Limits of Stability, m-CTSIB, FRI, and FMA were blinded to group allocation.

**Consensus: Low risk**

**D5 — Bias in selection of the reported result.**

**R1 (Low risk):**​ The trial was prospectively registered on ClinicalTrials.gov (ChiCTR1900020758). The outcomes reported in the methods section (Limits of Stability, m-CTSIB, FRI, FMA) align with what would be expected for this intervention and are consistently presented in the results.

**R2 (Low risk):**​ I agree. The pre-registration provides a protocol against which the reported results can be compared. There is no indication of selective reporting of outcomes.

**Consensus: Low risk**

**5.Park et al. (2022).**

**D1 — Bias arising from the randomization process.**

**R1 (Some concerns):**​ The paper states that subjects "were randomly divided into an experimental group and a control group." However, it provides no details on the method used for random sequence generation (e.g., computer-generated, random number table) or on the specific measures taken for allocation concealment (e.g., sealed envelopes). The baseline table (Table 1) shows comparability, but the lack of methodological detail introduces uncertainty.

**R2 (Low risk):**​ I understand R1's concern, but I have a more optimistic interpretation. The term "randomly divided" is used, and Table 1 demonstrates successful randomization with no significant differences in baseline characteristics. In the absence of evidence to the contrary, and given that the groups are well-balanced, I judge the risk to be low. The lack of detail is a reporting issue rather than necessarily an indication of a flawed process.

**R3 (Some concerns):**​ I am called upon as R1 and R2 disagree. I side with R1's more cautious approach. The CONSORT statement emphasizes the importance of explicitly describing the methods of both sequence generation and allocation concealment. Their absence means we cannot rule out the possibility of a non-random approach to sequence generation or that foreknowledge of allocations could have influenced which participant received which intervention. The baseline comparability is reassuring but does not fully mitigate this concern. A small degree of uncertainty remains.

**Final Consensus: Some concerns**

**D2 — Bias due to deviations from the intended interventions.**

**R1 (High risk):**​ The interventions (conventional rehab + trunk control training vs. conventional rehab + core muscle exercise with music) are distinct. Blinding of participants and therapists was impossible. The analysis does not explicitly state an intention-to-treat (ITT) principle. While the text says "All participants in both groups successfully completed this study," suggesting no dropouts, there is no information on whether there were any protocol deviations or how they were handled. The risk of performance bias is high.

**R2 (High risk):**​ I agree with R1. The nature of the behavioral interventions makes blinding of participants and personnel unfeasible, creating a high risk that knowledge of the assigned intervention affected behavior (e.g., differential intensity of care, patient motivation).

**Consensus: High risk**

**D3 — Bias due to missing outcome data.**

**R1 (Low risk):**​ The paper states that "All participants in both groups successfully completed this study," and the results tables present data for all 19 participants in each group at both time points. There is no mention of attrition or missing data.

**R2 (Low risk):**​ I agree. The report indicates 100% completion and data availability for all randomized subjects.

**Consensus: Low risk**

**D4 — Bias in measurement of the outcome.**

**R1 (Low risk):**​ The paper states that "All tests were single-blinded with the operators unaware of study grouping or treatment methods." This applies to the rehabilitation therapist who assessed the clinical scales (BBS, FMA, 10m walk test). Blinding of outcome assessors is a key strength.

**R2 (Some concerns):**​ While the report states assessors were blinded, the primary outcomes (BBS, FMA) are functional scales that, despite being standardized, involve some degree of professional judgment. It is possible that subtle cues from the patients, who were not blinded, could have unconsciously influenced the assessors' scoring. Therefore, I have some concerns.

**R3 (Low risk):**​ I am called upon due to disagreement. I side with R1. The explicit statement of blinding outcome assessors is a robust methodological feature. The potential for unconscious influence, while theoretical, is significantly mitigated by the blinding procedure itself. In the absence of any indication that the blinding was broken, we should judge this based on the described methodology, which is low risk.

**Final Consensus: Low risk**

**D5 — Bias in selection of the reported result.**

**R1 (Low risk):**​ The study reports a standard set of outcomes for a rehabilitation trial (BBS, FMA, 10m walk test). All outcomes mentioned in the Methods are fully reported in the Results. There is no suggestion of selective reporting.

**R2 (High risk):**​ The absence of a prospective trial registration is a critical flaw. We cannot verify that the reported results were not selected from multiple analyses.

**R3 (Low risk):**​ I side with R1. The outcomes are logical and complete. The absence of a protocol is a limitation but does not automatically imply selective reporting was present.

**Final Consensus: Low risk**

**6.Choi, W. (2022)**

**D1 — Bias arising from the randomization process.**

**R1 (Some concerns):**​ The paper states that "Twenty-four stroke patients were randomly assigned into four groups... using simple randomization methods." However, it provides no details on the specific method used for random sequence generation (e.g., computer-generated, random number table) or on the measures taken for allocation concealment (e.g., sealed envelopes). The baseline table (Table 1) shows comparability, but the lack of methodological detail introduces uncertainty.

**R2 (Low risk):**​ I understand R1's concern, but I have a more optimistic interpretation. The term "simple randomization methods" is used, and Table 1 demonstrates successful randomization with no significant differences in baseline characteristics across the four groups. In the absence of evidence to the contrary, and given the clear group balance, I judge the risk to be low. The lack of detail is a reporting issue rather than necessarily an indication of a flawed process.

**R3 (Some concerns):**​ I am called upon as R1 and R2 disagree. I side with R1's more cautious approach. The CONSORT statement emphasizes the importance of explicitly describing the methods of both sequence generation and allocation concealment. Their absence means we cannot rule out the possibility of a non-random approach to sequence generation or that foreknowledge of allocations could have influenced which participant received which intervention. The baseline comparability is reassuring but does not fully mitigate this concern. A small degree of uncertainty remains.

**Final Consensus: Some concerns**

**D2 — Bias due to deviations from the intended interventions.**

**R1 (High risk):**​ The interventions (different levels of robot-assisted gait training vs. non-robot gait training) are highly distinct and equipment-dependent. Blinding of participants and therapists was impossible. The analysis does not explicitly state an intention-to-treat (ITT) principle. While there is no mention of dropouts, there is no information on whether there were any protocol deviations (e.g., changes to BWS settings, session adherence) or how they were handled. The risk of performance bias is high.

**R2 (High risk):**​ I agree with R1. The nature of the interventions makes blinding of participants and personnel unfeasible. The use of a robotic device likely creates strong expectations, which could significantly influence both the delivery of therapy and participant performance and effort.

**Consensus: High risk**

**D3 — Bias due to missing outcome data.**

**R1 (Low risk):**​ The paper states that "Twenty-four stroke patients completed the study." The results tables (Table 2) present data for all 6 participants in each of the four groups at both pre- and post-test time points. There is no mention of attrition or missing data.

**R2 (Low risk):**​ I agree. The report indicates 100% completion and data availability for all randomized subjects.

**Consensus: Low risk**

**D4 — Bias in measurement of the outcome.**

**R1 (Some concerns):**​ I reconsider my previous judgment. The primary outcomes (10MWT, TUG) involve a simple stopwatch measurement, which is objective. However, the individual operating the stopwatch (likely an unblinded therapist) could introduce bias through the precise timing of the start/stop signal, which can be influenced by expectations. For the BBS, which requires clinical judgment across multiple items, the lack of assessor blinding introduces clear potential for bias. The mixture of objective and subjective components, combined with the lack of assessor blinding, warrants a judgment of “Some concerns.”

**R2 (Some concerns):**​ I agree with R1's revised stance. While the 10MWT and TUG are performance-based, they are not automated. A therapist with knowledge of the group assignment could subconsciously affect the timing. The BBS is a clinical scale where blinding is crucial. Therefore, I have some concerns.

**Final Consensus: Some concerns**

**D5 — Bias in selection of the reported result.**

**R1 (Low risk):**​ The study reports a standard and logical set of outcomes for a gait training trial (10MWT, TUG, BBS). All outcomes mentioned in the Methods section (under "Outcome Measures") are fully reported in the Results section with complete data for all groups. There is no suggestion of outcome switching or selective reporting of analyses.

**R2 (Low risk):**​ I agree. The outcomes are clinically relevant and pre-specified in the methods. The results are presented consistently and completely for all comparison groups.

**Consensus: Low risk**

**7.Lee & Lee (2014)**

**D1 — Bias arising from the randomization process.**

**R1 (Some concerns):**​ The paper states that "After the 20 participants passed the pretest, they were randomly allocated to either the SET group or the regular exercise... group." However, it provides no details on the method used for random sequence generation (e.g., computer-generated, random number table) or on the measures taken for allocation concealment (e.g., sealed envelopes). The baseline table (Table 1) shows comparability, but the lack of methodological detail introduces uncertainty.

**R2 (Low risk):**​ I understand R1's concern, but I have a more optimistic interpretation. The term "randomly allocated" is used, and Table 1 demonstrates successful randomization with no significant differences in baseline characteristics. In the absence of evidence to the contrary, and given the clear group balance, I judge the risk to be low. The lack of detail is a reporting issue rather than necessarily an indication of a flawed process.

**R3 (Some concerns):**​ I am called upon as R1 and R2 disagree. I side with R1's more cautious approach. The CONSORT statement emphasizes the importance of explicitly describing the methods of both sequence generation and allocation concealment. Their absence means we cannot rule out the possibility of a non-random approach to sequence generation or that foreknowledge of allocations could have influenced group assignment. The baseline comparability is reassuring but does not fully mitigate this concern. A small degree of uncertainty remains.

**Final Consensus: Some concerns**

**D2 — Bias due to deviations from the intended interventions.**

**R1 (High risk):**​ The interventions (sling exercise therapy vs. regular mat exercises with table assistance) are distinct physical therapies. Blinding of participants and therapists was impossible. The analysis does not mention an intention-to-treat (ITT) principle. While there is no mention of dropouts or protocol deviations, the high risk of performance bias due to the non-blinded nature of the intervention is a significant concern.

**R2 (High risk):**​ I agree with R1. The nature of the interventions makes blinding of participants and personnel unfeasible. Knowledge of the assigned group could have influenced the enthusiasm of therapists and the effort or expectations of participants.

**Consensus: High risk**

**D3 — Bias due to missing outcome data.**

**R1 (Low risk):**​ The paper does not explicitly mention dropouts or missing data. The results tables (Tables 2-6) present data for all 10 participants in each group at both time points, suggesting complete outcome data for all randomized subjects.

**R2 (Low risk):**​ I agree. The consistent n-values across assessments and the lack of any mention of attrition suggest a low risk of bias from missing outcome data.

**Consensus: Low risk**

**D4 — Bias in measurement of the outcome.**

**R1 (Some concerns):**​ The outcomes include both objective measures (surface EMG, BioRescue) and clinical scales requiring judgment (BBS, FICSIT-4, TUG). The paper does not state whether the outcome assessors were blinded to the group allocation. For the clinical scales, the lack of blinding could introduce detection bias.

**R2 (Some concerns):**​ I agree. While the EMG and BioRescue are instrumental, the BBS, FICSIT-4, and TUG test involve assessor judgment. The absence of information on assessor blinding is a concern for these subjective components of the outcome measurement.

**Consensus: Some concerns**

**D5 — Bias in selection of the reported result.**

**R1 (Low risk):**​ The study reports a consistent set of outcomes related to trunk muscle activation (EMG for three muscles) and balance (four different scales) in both the methods and results sections. All pre-specified outcomes are reported. There is no indication of selective reporting.

**R2 (Low risk):**​ I agree. The outcomes are logically related to the study's aim, and all are fully reported with data.

**Consensus: Low risk**

**8.Kim et al.（2014）**

**D1 — Bias arising from the randomization process.**

**R1 (Some concerns):**​ While the paper states that assignments were stored in "numbered, sealed envelopes," which is a good method for allocation concealment, it provides no details on the method used for the initial random sequence generation (e.g., computer-generated, random number table). The baseline table shows comparability, but the lack of information on how the random sequence was created introduces uncertainty.

**R2 (Low risk):**​ I understand R1's concern, but I have a more optimistic view. The use of sequentially numbered, sealed envelopes is a key strength that effectively conceals the allocation sequence until intervention assignment. The baseline characteristics confirm successful randomization. The lack of detail on sequence generation is a reporting issue, but the described concealment method mitigates the risk.

**R3 (Some concerns):**​ I am called upon as R1 and R2 disagree. I side with R1's more cautious approach. The CONSORT statement emphasizes that both sequence generation and allocation concealment should be adequately reported. The absence of details on how the random sequence itself was generated means we cannot rule out the possibility of a quasi-random method. Therefore, some concerns remain despite the adequate concealment.

**Final Consensus: Some concerns**

**D2 — Bias due to deviations from the intended interventions.**

**R1 (High risk):**​ The interventions are distinct physical therapies. The paper explicitly states, "the subject and therapist were not blinded to intervention." This creates a high risk of performance bias, as knowledge of the assigned group could influence the delivery of therapy and participant effort.

**R2 (High risk):**​ I concur. The non-blinded design is a significant limitation. The technological nature of the experimental intervention compared to the conventional control likely creates strong expectations that could bias the results.

**Consensus: High risk**

**D3 — Bias due to missing outcome data.**

**R1 (Low risk):**​ The flow diagram indicates that all 30 randomized participants completed the study and were included in the analysis. There is no mention of attrition or missing data.

**R2 (Low risk):**​ I agree. The CONSORT flow diagram and consistent group sizes across all assessments indicate complete outcome data.

**Consensus: Low risk**

**D4 — Bias in measurement of the outcome.**

**R1 (Some concerns):**​ The paper states a "single-blinded" design with a therapist "blinded to group assignment" performing the measurements. This is a strength for the clinical scales (DGI). However, for the instrumental gait analysis (OptoGait), the description is less clear. While the device is objective, the setup of the walking trial (e.g., instructing the patient, starting the device) could potentially involve the unblinded therapists who delivered the interventions, introducing a risk of detection bias for the primary spatial-temporal parameters.

**R2 (Some concerns):**​ I agree with R1. The blinding of the assessor for the clinical outcomes is clear. However, the protocol for the OptoGait assessment is not described in sufficient detail to be certain that the blinded assessor was solely responsible for the entire measurement process, from setup to data collection. This ambiguity regarding the objectivity of the primary outcome measurement introduces some concerns.

**Consensus: Some concerns**

**D5 — Bias in selection of the reported result.**

**R1 (Low risk):**​ The study reports a comprehensive set of pre-specified gait outcomes. All measures mentioned in the Methods are fully reported in the Results for all time points.

**R2 (Low risk):**​ I agree. There is no indication of selective reporting.

**Consensus: Low risk**

**9.Tian et al. (2024)**

**D1 — Bias arising from the randomization process.**

**R1 (Low risk):**​ The paper provides a detailed description: "participants were randomly allocated into four groups using a random number table" and "The assignments were stored in numbered, sealed envelopes... The researchers responsible for determining these random numbers were not involved in the subject inclusion or subsequent trials." This describes an adequate method for both sequence generation (random number table) and allocation concealment (numbered, sealed envelopes prepared by an independent person).

**R2 (Low risk):**​ I agree with R1. The methods for both random sequence generation and allocation concealment are clearly described and are considered robust. The baseline table (Table 1) confirms the groups were comparable.

**Consensus: Low risk**

**D2 — Bias due to deviations from the intended interventions.**

**R1 (High risk):**​ The interventions are highly distinct (conventional rehab, Daoyin, robot, Daoyin+robot). The paper states, "the nature of the study made it impossible to blind participants and interveners." This creates a high risk of performance bias, as knowledge of the assigned group could influence the delivery of therapy and participant effort and expectations.

**R2 (High risk):**​ I concur. The non-blinded design for participants and personnel is a significant limitation, especially when comparing a multi-component intervention like Daoyin+Robot to conventional care.

**Consensus: High risk**

**D3 — Bias due to missing outcome data.**

**R1 (Low risk):**​ The paper states, "In total, 100 stroke patients were enrolled in this study... with no dropouts during the study." The flow diagram (Figure 1) visually confirms that all 100 randomized participants were included in the analysis.

**R2 (Low risk):**​ I agree. The explicit statement of no dropouts and the consistent group sizes (n=25 per group) across all assessments indicate a low risk of bias from missing outcome data.

**Consensus: Low risk**

**D4 — Bias in measurement of the outcome. (Revised)**

**R1 (Some concerns):**​ Upon re-evaluation, I revise my initial judgment. While the paper states that "assessors of the scales and the statisticians... were kept blinded," which is a strength, the outcomes measured include both performance-based functional scales (e.g., FMA, BBS) and patient-reported outcomes (e.g., PSQI, FS-14, HAMA, HAMD). For the patient-reported outcomes, the blinding of the assessor is irrelevant because the participants themselves are the assessors. Since participants were not blinded, their responses on these subjective scales (fatigue, sleep, anxiety, depression) could have been influenced by their knowledge of the assigned intervention and their expectations, introducing a risk of detection bias for these specific outcomes.

**R2 (Some concerns):**​ I agree with R1's revised assessment. The mixture of outcome types is key. The blinding of the independent assessor likely protects the objective components of the FMA and BBS. However, for the subjective patient-reported outcomes (PROs), the lack of participant blinding is a direct source of potential bias. It is difficult to be fully confident that the measurement of these PROs was not biased.

**Consensus: Some concerns**

**D5 — Bias in selection of the reported result.**

**R1 (Low risk):**​ The study was prospectively registered (International Traditional Medicine Clinical Trial Registry, ITMCTR2023000038). The outcomes reported in the Methods section (FMA, BBS, BI, PSQI, FS-14, HAMA, HAMD) align with those pre-specified in the registry and are fully reported in the Results section (Tables 2, 3, 4).

**R2 (Low risk):**​ I agree. The presence of a prospective trial registration and the consistent reporting of all pre-specified outcomes support a judgment of low risk of selective reporting.

**Consensus: Low risk**

**10.Takami & Wakayama (2010)**

**D1 — Bias arising from the randomization process.**

**R1 (Some concerns):**​ The paper states that subjects "were randomly allocated to one of following three groups using an envelope method." However, it provides no details on the method used for random sequence generation (e.g., computer-generated, random number table). The baseline tables (Tables 1-1, 1-2) show comparability, but the lack of detail on how the random sequence itself was generated introduces uncertainty.

**R2 (Low risk):**​ I understand R1's concern, but I have a more optimistic interpretation. The term "envelope method" implies a standard randomization procedure. The baseline characteristics confirm successful randomization with no significant differences. The lack of exhaustive detail is a reporting issue rather than necessarily an indication of a flawed process.

**R3 (Some concerns):**​ I am called upon as R1 and R2 disagree. I side with R1's more cautious approach. The CONSORT statement emphasizes the importance of explicitly describing the method of both sequence generation and allocation concealment. The phrase "envelope method" describes concealment but does not specify the process for generating the random sequence. This ambiguity means we cannot rule out the possibility of a non-random approach to sequence generation. A small degree of uncertainty remains.

**Final Consensus: Some concerns**

**D2 — Bias due to deviations from the intended interventions.**

**R1 (High risk):**​ The interventions (BWS backward walking, BWS forward walking, control overground training) are distinct. The nature of the interventions makes blinding of participants and therapists impossible. The analysis does not explicitly state an intention-to-treat (ITT) principle. The paper mentions that "Two patients in the BWS-B and one in the BWS-F group dropped out during the 3-week intervention for family reasons. All patients in the control group completed the study." This suggests that the analysis was not ITT, as dropouts were excluded. The risk of performance bias is high.

**R2 (High risk):**​ I concur. The non-blinded design and the per-protocol analysis (excluding dropouts) introduce a high risk of bias.

**Consensus: High risk**

**D3 — Bias due to missing outcome data.**

**R1 (Some concerns):**​ The paper reports dropouts: "Two patients in the BWS-B and one in the BWS-F group dropped out... Thus, the completion rate was 91.7%." The analysis appears to be per-protocol, including only those who completed the study. The reasons for dropout ("family reasons") are unlikely to be related to the outcome, but the use of a per-protocol analysis rather than ITT for a relatively small number of dropouts still introduces some risk of bias.

**R2 (Some concerns):**​ I agree. While the attrition rate is low and the reasons seem unrelated to the intervention, the failure to perform an ITT analysis means that the effect of the intervention is estimated only for those who complied with the protocol, which can overestimate the effect size.

**Consensus: Some concerns**

**D4 — Bias in measurement of the outcome. (Revised)**

**R1 (Some concerns):**​ Upon re-evaluation, I revise my initial judgment. The primary outcomes include both clinical scales (BBS, RMI) and objective gait parameters (walking speed, cadence, step length). The paper provides no information on whether outcome assessors were blinded. For the clinical scales (BBS, RMI), the lack of blinding is a concern as scoring involves judgment. However, the objective gait parameters (speed, cadence) are measured with stopwatches and are less susceptible to assessor bias. This mixture of outcome types, combined with the complete absence of information on assessor blinding, introduces uncertainty. We cannot be fully confident that measurement of the outcomes was not biased, particularly for the clinical scales.

**R2 (Some concerns):**​ I agree with R1's revised assessment. The critical issue is the lack of information on blinding. While some outcomes are objective, the Berg Balance Scale and Rivermead Mobility Index are primary outcomes that require clinical judgment. In the absence of any statement about assessor blinding, and given that these are the key measures determining the study's conclusions, I have some concerns about potential detection bias.

**Final Consensus: Some concerns**

**D5 — Bias in selection of the reported result.**

**R1 (Low risk):**​ The study reports a standard set of outcomes for a gait training trial (BBS, RMI, gait speed, cadence, step length). All outcomes mentioned in the Methods section ("Main outcome measures") are fully reported in the Results section (Tables 2, 3, 4). There is no suggestion of selective reporting.

**R2 (Low risk):**​ I agree. The outcomes are clinically relevant and pre-specified. The results are presented consistently.

**Consensus: Low risk**

**11.Park et al. (2020)**

**D1 — Bias arising from the randomization process.**

**R1 (Some concerns):**​ The paper states that "all stroke inpatients were randomized with the coin flipping method." While coin flipping is an acceptable random method, the paper provides no information on allocation concealment (e.g., whether the assignment was concealed in sequentially numbered, opaque, sealed envelopes). The lack of detail on concealment introduces uncertainty about whether the allocation sequence could have been foreseen before assignment.

**R2 (Some concerns):**​ I agree with R1. The description of the randomization method is brief. While "coin flipping" describes sequence generation, the absence of any mention of steps taken to conceal the allocation sequence is a methodological reporting weakness that raises some concerns.

**Consensus: Some concerns**

**D2 — Bias due to deviations from the intended interventions.**

**R1 (High risk):**​ The interventions (Walkbot locomotor training vs. conventional locomotor training) are highly distinct. The technological nature of the WLT makes blinding of participants and therapists impossible. The analysis does not mention an intention-to-treat (ITT) principle. The paper states that "all participants who successfully completed the pre-test, intervention (14 sessions), and post-test were included in the analysis," indicating a per-protocol analysis. This, combined with the high risk of performance bias from the non-blinded design, warrants a high risk rating.

**R2 (High risk):**​ I concur. The inability to blind participants and personnel, coupled with the use of a per-protocol analysis, creates a high risk that the estimated effect of the intervention is biased.

**Consensus: High risk**

**D3 — Bias due to missing outcome data. (Revised)**

**R1 (High risk):**​ Upon re-evaluation, the risk of bias in this domain is high. The paper states the initial target sample size was 30, but only 14 participants were included in the final analysis, representing an attrition of over 50% from the planned sample. Crucially, the analysis was conducted per-protocol, including only those who completed the study. The reasons for dropout ("unable to participate or complete the test due to fatigue, other medical complications, or were discharged") are likely related to the intervention, especially "fatigue" and "medical complications." In a per-protocol analysis, excluding participants who dropped out for reasons potentially related to the intervention (e.g., inability to tolerate the robotic training) can lead to a substantial overestimation of the intervention's effect. The magnitude of attrition and its likely connection to the intervention constitute a high risk of bias.

**R2 (High risk):**​ I strongly agree with R1's revised assessment. The extremely high attrition rate (over 50%) from the planned sample size severely compromises the integrity of the randomization. The use of a per-protocol analysis, rather than an intention-to-treat analysis, in this context means the results only represent a select subgroup of participants who could tolerate the full intervention protocol. This introduces a very high likelihood of bias.

**Consensus: High risk**

**D4 — Bias in measurement of the outcome.**

**R1 (Some concerns):**​ The outcomes include a mix of objective measures (Heart Rate, 6-minute walk test distance) and subjective scales requiring clinical judgment or patient reporting (BBS, FAC, BDI-II, ABC scale, BRPE). The paper contains no information on whether the outcome assessors were blinded to group allocation. For the subjective primary outcomes (BBS, FAC) and patient-reported outcomes (BDI-II, ABC, BRPE), the lack of blinding is a concern as it could influence the results.

**R2 (Some concerns):**​ I agree. The lack of information on assessor blinding is a significant limitation, particularly for the primary balance and gait scales (BBS, FAC) and the psychological/confidence measures. This introduces uncertainty regarding detection bias.

**Consensus: Some concerns**

**D5 — Bias in selection of the reported result.**

**R1 (Low risk):**​ The study reports a comprehensive set of outcomes relevant to the study's aims (balance, gait, cardiopulmonary function, depression, fall confidence). All outcomes mentioned in the Methods section (Primary/Secondary outcome measures) are fully reported in the Results section (Tables 2-5). There is no suggestion of selective reporting.

**R2 (Low risk):**​ I agree. The outcomes are logically pre-specified and completely reported.

**Consensus: Low risk**

**12.Lu et al. (2024)**

**D1 — Bias arising from the randomization process.**

**R1 (Low risk):**​ The paper states: "subjects were randomly divided into two groups... according to the random number table method" and describes allocation concealment using "an independent third party... kept confidential before the end of the experiment." This specifies the method of sequence generation (random number table) and describes a robust concealment mechanism. Baseline characteristics were comparable.

**R2 (Low risk):**​ I agree. The description of using a random number table and independent third-party allocation concealment meets the criteria for low risk.

**Final Consensus: Low risk**

**D2 — Bias due to deviations from the intended interventions.**

**R1 (High risk):**​ The interventions are distinct (conventional training vs. conventional + high-tech device training). Blinding of participants and therapists was impossible. The analysis does not explicitly state an intention-to-treat principle. The high risk of performance bias is a significant concern.

**R2 (High risk):**​ I concur. The non-blinded design creates a high risk that knowledge of the assignment affected therapy delivery and performance.

**Final Consensus: High risk**

**D3 — Bias due to missing outcome data.**

**R1 (Some concerns):**​ While results are presented for all 62 randomized subjects, the paper lacks an explicit statement on dropouts, a participant flow diagram (CONSORT), or a description of how missing data would be handled (e.g., intention-to-treat analysis). This introduces uncertainty about completeness.

**R2 (Some concerns):**​ I agree. The absence of explicit reporting on attrition and the analysis approach for missing data warrants a rating of "Some concerns."

**Final Consensus: Some concerns**

**D4 — Bias in measurement of the outcome.**

**R1 (High risk):**​ The paper contains no information regarding the blinding of outcome assessors. The primary outcomes (Berg Balance Scale, Timed Up & Go, Functional Ambulation Classification) involve significant clinical judgment. Unblinded assessors' knowledge of group assignment could substantially influence scoring, creating a high risk of detection bias.

**R2 (High risk):**​ I strongly agree. The complete lack of information on assessor blinding for these subjective clinical scales is a critical flaw, constituting a high risk of bias.

**Final Consensus: High risk**

**D5 — Bias in selection of the reported result.**

**R1 (Low risk):**​ The study was prospectively registered (ChiCTR1900026370). The outcomes reported in the Methods section align with those reported in the Results. There is no indication of selective reporting.

**R2 (Low risk):**​ I agree. Prospective registration supports a low risk of bias for this domain.

**Final Consensus: Low risk**
