# Supplementary material for: The effects of suspension-supported training on dynamic balance capacity in stroke patients: a systematic review and meta-analysis enhanced by XGBoost machine learning
Source: Front Med (Lausanne). 2026 Feb 9;13:1747067. doi: 10.3389/fmed.2026.1747067 (PMC12926393; doi:10.3389/fmed.2026.1747067)
Supplement: Supplementary file 3 [file Table_3.DOCX]

**Consistency of Intervention Methods in Included RCTs with the Definition of "Suspension-Supported Training"**

**I. Operational Definition (Used for Consistency Determination)**

- This explanation provides the following operational definition of "Suspension Support Training":
- The use of suspension devices such as slings, suspension ropes, or body weight support/weight-loss devices during training to provide support or suspension for the torso or limbs.
- These devices enable partial weight-bearing/unloading (e.g., weight reduction by a percentage) or localized suspension, allowing participants to perform training under safer, controlled loads.
- The purpose of suspension support is to serve task-related training (such as walking, standing, balance, core stability, etc.), rather than simply passive fixation.

**II. Item-by-Item Verification Results (All Conforming to the Above Definition)**

Note: The following content is refined and determined based on the descriptions of the intervention protocols/devices in the full texts of the 12 RCTs you provided.

| Study Information (Author/Title) | Suspension-Supported Intervention Approach (Refined) | Conformity Determination and Reasons (Corresponding Definition Elements) |
| --- | --- | --- |
| Lu C. (2024). Observation and study on the balance function and control ability of stroke patients treated with Bobath technique combined with suspension weight loss training. | "suspension weight loss training" is added to the conventional rehabilitation/Bobath; Use suspension belts/suspension belts to suspension support and weight loss support for limbs or trunk-related parts for balance and trunk control training. | - Presence of a well-defined suspension/weight reduction training device (suspension belt/suspension belt, etc.). - Support/weight reduction is achieved through suspension, reducing lower limb load and fall risk. - Complete task training such as balance and trunk control under support conditions. |
| Yu Q. et al. (2020). Effects of Body Weight Support–Tai Chi Footwork Training on Balance Control and Walking Function in Stroke Survivors with Hemiplegia: A Pilot RCT. | BWS-TC foot training: Subjects wear a safety sling and attach to the top suspension system; Tai Chi foot training (center of gravity shift/gait pace) was performed under body weight support (BWS) conditions, and the support ratio was gradually reduced according to the protocol. | - Body weight support with top suspension system + safety sling. - The amount of support is adjustable (gradually reducing BWS), which is a training under controlled load. - For walking/balance-related task training (Tai Chi footwork, gait function). |
| Chen X. et al. (2020). Effects of rehabilitation training of core muscle stability on stroke patients with hemiplegia (Pakistan Journal of Medical Sciences). | Core stability training adopts the "multi-point multi-axis suspension training system": the knees, ankles and other parts are suspended through inelastic suspension straps, and elastic traction is applied at the waist to raise the pelvis/maintain a level position; Complete core training such as bridge pose and swing under suspension support. | - The use of multi-point multi-axis suspension system and suspension belt is clearly used. - Suspension/traction provides support to the trunk and lower extremities, relieves weight and creates controlled instability. - Task training for core stabilization and postural control. |
| Huang W. et al. (2019). Body weight support–Tai Chi footwork training for balance of stroke survivors with fear of falling: A pilot RCT (Complementary Therapies in Clinical Practice). | BWS-TC foot training: Partial weight loss is provided through a body weight support system during the training process (e.g., about 40% support at the beginning and gradually decreased), allowing subjects to practice footwork and gait-related tasks under a safe and controlled load. | - The body weight support system (BWS) is the core device. - Controllable load is achieved through proportional weight reduction/support. - The training consists of footwork/gait and balance tasks. |
| Park J-S. et al. The Effects of Core Stability Exercise with Music on Balance and Functional Activity in Stroke. | Core stability training is carried out under the support condition of "elastic suspension/hanging rope": the knees, ankles, waist and other parts are suspended and supported through the suspension rope (or elastic suspension belt), and the pelvic lift, bridge pose, swing and other movements are completed, and the rhythm of music is matched. | - Suspension support devices such as suspension ropes/elastic suspension straps are present. - Provides support and load adjustment to the lower extremities/pelvis through local suspension. - It is used for core stability, balance, and functional activity-related training. |
| Choi W. (2022). Effects of Robot-Assisted Gait Training with Body Weight Support on Gait and Balance in Stroke Patients (Int. J. Environ. Res. Public Health). | Robotic walking training (Lokomat, etc.) combined with body weight support: Subjects wear a sling and connect a suspension device to perform treadmill walking training under a set proportion of body weight support conditions (the amount of support can be set/adjusted according to the protocol). | - Weight support is provided with a sling/suspension device. - The support level is adjustable and belongs to controlled load training. - Task-oriented training for walking and balance ability. |
| Lee J.S. & Lee H.G. (2013). Effects of Sling Exercise Therapy on Trunk Muscle Activation and Balance in Chronic Hemiplegic Patients (J. Phys. Ther. Sci.). | Suspension (sling) exercise therapy: hanging a rope from the ceiling and attaching a suspension strap/sling; During training, the ankle and other joints are placed in the sling, and auxiliary elastic ropes can be used to provide support/assistance to complete trunk stability and balance training in a suspension environment. | - The core unit is the sling/sling system, which is typical of suspension training. - Support/assistance and load adjustment can be realized by elastic ropes, etc. - The purpose of the training is to train tasks such as trunk activation, balance and posture control. |
| Kim K.H. et al. Effects of progressive backward body weight-supported treadmill training on gait ability in chronic stroke patients: A randomized controlled trial. | Progressive backward BWSTT: Weight support training with the help of a "suspension device" on the treadmill; The proportion of weight support decreases weekly (e.g., 40% in the first week→ 30% in the second week→ 20% in the third week→ 10% in the fourth week), with increasing walking speed and therapist assistance. | - Clearly use suspension devices to provide body weight support during treadmill training. - The amount of support decreases week by week, reflecting adjustable and controllable weight reduction support. - The training consists of walking tasks (backward treadmill training) and gait ability improvement. |
| Tian H. et al. (2024). Efficacy of Daoyin combined with lower limb robot as a comprehensive rehabilitation intervention for stroke patients: a randomized controlled trial. | The lower limb robotic walking training system includes lumbar support and suspension/suspension systems to stabilize the torso and pelvis, provide support and reduce the requirement for active balance; Robot-assisted walking training (and combined with guidance) was completed in this supported condition. | - The training platform contains a clear "suspension system". - Controllable load/posture control is achieved through support and immobilization of the trunk/pelvis. - It is used for task-oriented rehabilitation such as walking training. |
| Lu W. (2024). Effects of visual feedback balance system combined with weight loss training system on balance ability in stroke: a randomized controlled exploratory study. | Visual feedback balance training combined with a "weight loss system": Attach a suspension rope to a safety sling/piggyback device, and achieve weight loss by fixing pulleys, etc., to perform standing and balance training under weight loss support conditions. | - There are weight reduction (weight support) devices such as slings + suspension ropes + pulleys. - Provides weight unloading/weight loss support to reduce training risk and control load. - Task training for balance and posture control. |
| Takami A. & Wakayama S. (2010). Effects of Partial Body Weight Support while Training Acute Stroke Patients to Walk Backwards on a Treadmill: A Controlled Clinical Trial Using Randomized Allocation (J. Phys. Ther. Sci.). | Partial Body Weight Support (PBWS) Treadmill Training: Subjects perform backward treadmill walking under the condition of the body weight support system; Weight support starts at about 30% and gradually decreases to 0% to promote safe walking practice and gait learning. | - PBWS system as the core (partial body weight support). - The amount of support decreases according to the scheme, which is a controllable load training. - The training content is a treadmill walking task (backward walking). |
| Park C. et al. Effects of innovative hip-knee-ankle interlimb coordinated robot training on ambulation, cardiopulmonary function, depression, and fall confidence in acute hemiplegia (NeuroRehabilitation, proof). | Robotic Walking Training with Bodyweight Support: Use a suspension vest and connect to the Walkbot sling/counterweight system with elastic straps to provide body weight support (e.g., initial 40–60% support and gradual reduction). | - It is clear that there are suspension support devices such as suspension vest + suspender + counterweight system. - It can provide and adjust the weight support ratio, which is a controlled weight loss support. - It is used for robot-assisted walking/walking ability-related training. |

**III. Overall Conclusion**

The intervention protocols in the 12 RCTs mentioned above all included "suspension/sling support devices" (such as body weight support systems, unloading systems, slings, harnesses, suspension ropes, or suspension and counterweight structures associated with robotic walking systems) and employed partial weight unloading/local suspension to facilitate task-related training (walking, balance, or core stability) under safe, controlled loading conditions. Therefore, all of these can be classified as "suspension-supported training" under the operational definition used in this study.
