# Supplementary material for: The effects of suspension-supported training on dynamic balance capacity in stroke patients: a systematic review and meta-analysis enhanced by XGBoost machine learning
Source: Front Med (Lausanne). 2026 Feb 9;13:1747067. doi: 10.3389/fmed.2026.1747067 (PMC12926393; doi:10.3389/fmed.2026.1747067)
Supplement: Supplementary file 4 [file Table_4.DOCX]

**1. PubMed​​**

("suspension-supported train*"[Title/Abstract] OR "suspension therap*"[Title/Abstract] OR "sling exercise*"[Title/Abstract] OR "sling therapy"[Title/Abstract] OR "redcord"[Title/Abstract] OR "TRX"[Title/Abstract]) AND ("Stroke"[MeSH Terms] OR "stroke*"[Title/Abstract] OR "poststroke"[Title/Abstract] OR "post-stroke"[Title/Abstract] OR "cerebrovascular accident"[Title/Abstract] OR "CVA"[Title/Abstract]) AND ("randomized controlled trial"[Publication Type] OR "controlled clinical trial"[Publication Type])) AND (randomizedcontrolledtrial[Filter]

Filters: English, Publication date to 2025/10/31

**2. Cochrane Library ​​**

(suspension-supported train* OR suspension therap* OR sling exercise* OR "sling therapy" OR redcord OR TRX) AND (stroke* OR poststroke OR post-stroke OR "cerebrovascular accident" OR CVA)

Publication Date to 31 October 2025, Language: English

**3. Web of Science**

TS=(sling exercise therapy OR suspension exercise training OR TRX OR sling training OR suspension-supported training) AND TS=(stroke patient* OR cerebrovascular accident patient* OR brain attack patient* OR acute cerebrovascular accident patient*)

Timespan: All years to 2025-10-31

**4. PsycINFO**

( " suspension-supported train*" OR "suspension therap*" OR "sling exercise*" OR "sling therapy" OR "redcord" OR "TRX" ) AND ("stroke*" OR "cerebrovascular accident" OR "CVA") AND ( "randomized controlled trial" )
