# Supplementary material for: The effects of suspension-supported training on dynamic balance capacity in stroke patients: a systematic review and meta-analysis enhanced by XGBoost machine learning
Source: Front Med (Lausanne). 2026 Feb 9;13:1747067. doi: 10.3389/fmed.2026.1747067 (PMC12926393; doi:10.3389/fmed.2026.1747067)
Supplement: Supplementary file 5 [file Table_5.DOCX]

Table of Contents

[Intervention Endpoint Sensitivity Analysis Plot 1](#_Toc27390)

[Intervention Endpoint Trim-and-Fill Plot 1](#_Toc20511)

[Posterior Distribution of Optimal Sling Exercise Dose 3](#_Toc7291)

[PPC: Density Overlay (Sling Exercise for Dynamic Balance) 4](#_Toc10718)

[PPC: Observed vs Predicted SMD by Dose 4](#_Toc14655)

[PPC: ECDF Overlay (Sling Exercise for Dynamic Balance) 5](#_Toc30636)

[Model Calibration Plot LOSO Validation (R² = 0.062) 7](#_Toc32041)

[Learning Curve 7](#_Toc22294)

[SHAP Dependence Plot: Exercise_time 8](#_Toc17422)

[SHAP Dependence Plot: Intervention_mode2 9](#_Toc7963)

[SHAP Dependence Plot: Intervention_mode1 9](#_Toc12680)

[SHAP Dependence Plot: Age 10](#_Toc13108)

[SHAP Dependence Plot: Subject_type 11](#_Toc22606)

[SHAP Dependence Plot: Intervention_frequency 12](#_Toc11219)

[SHAP Dependence Plot: Race 13](#_Toc25912)

[SHAP Dependence Plot: Intervention_time 14](#_Toc26592)

[SHAP Dependence Plot: Dose 15](#_Toc13137)

[SHAP Dependence Plot: Scale_type 16](#_Toc30447)

[Table 2. Subgroup Analysis of Suspension Training in Improving Dynamic Balance in Stroke Patients 16](#_Toc6579)

[Table 3. Detailed Data of Intervention Parameters 21](#_Toc2625)

[Table 4. Trial Sequential Analysis (TSA) Parameter Settings 21](#_Toc2297)

[Table 5. Trial Sequential Analysis (TSA) Summary of Results 22](#_Toc19161)

[Forest plot of the KHSJ random effects model analysis 23](#_Toc32141)

[Direction of SMD in different scales 23](#_Toc9512)

[Trial Sequential Analysis (TSA) 24](#_Toc13598)

[Inter-Rater Agreement Results for Each ROB2 Domain 24](#_Toc24346)

[GRADE Summary 25](#_Toc18683)


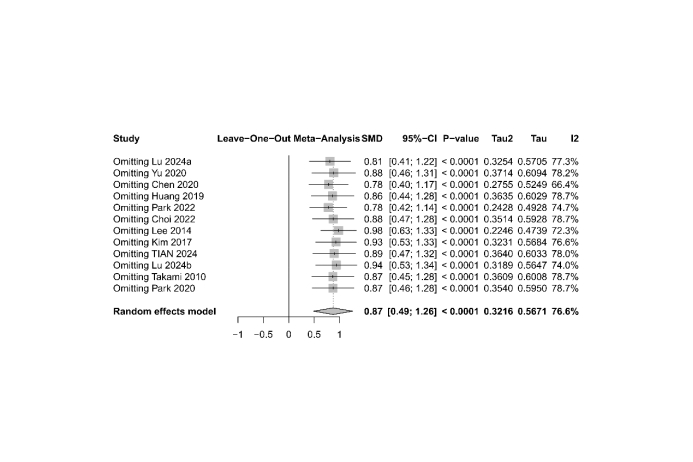


# Intervention Endpoint Sensitivity Analysis Plot


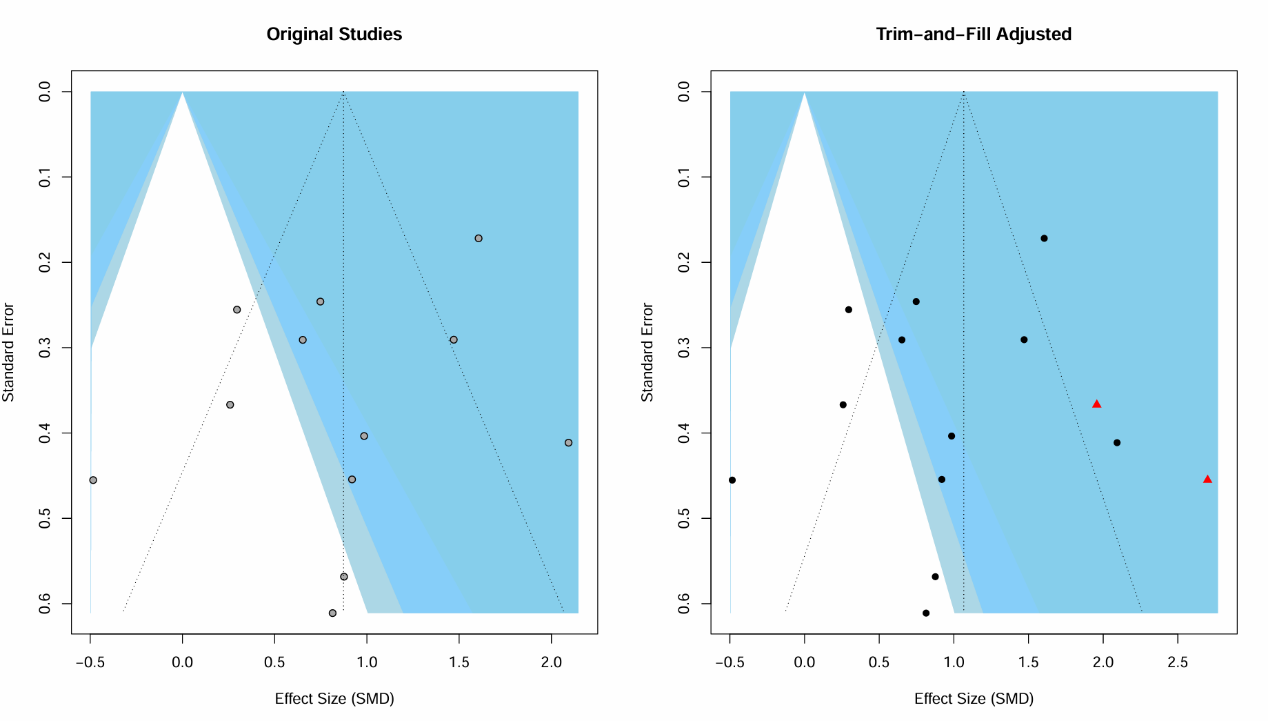


# Intervention Endpoint Trim-and-Fill Plot


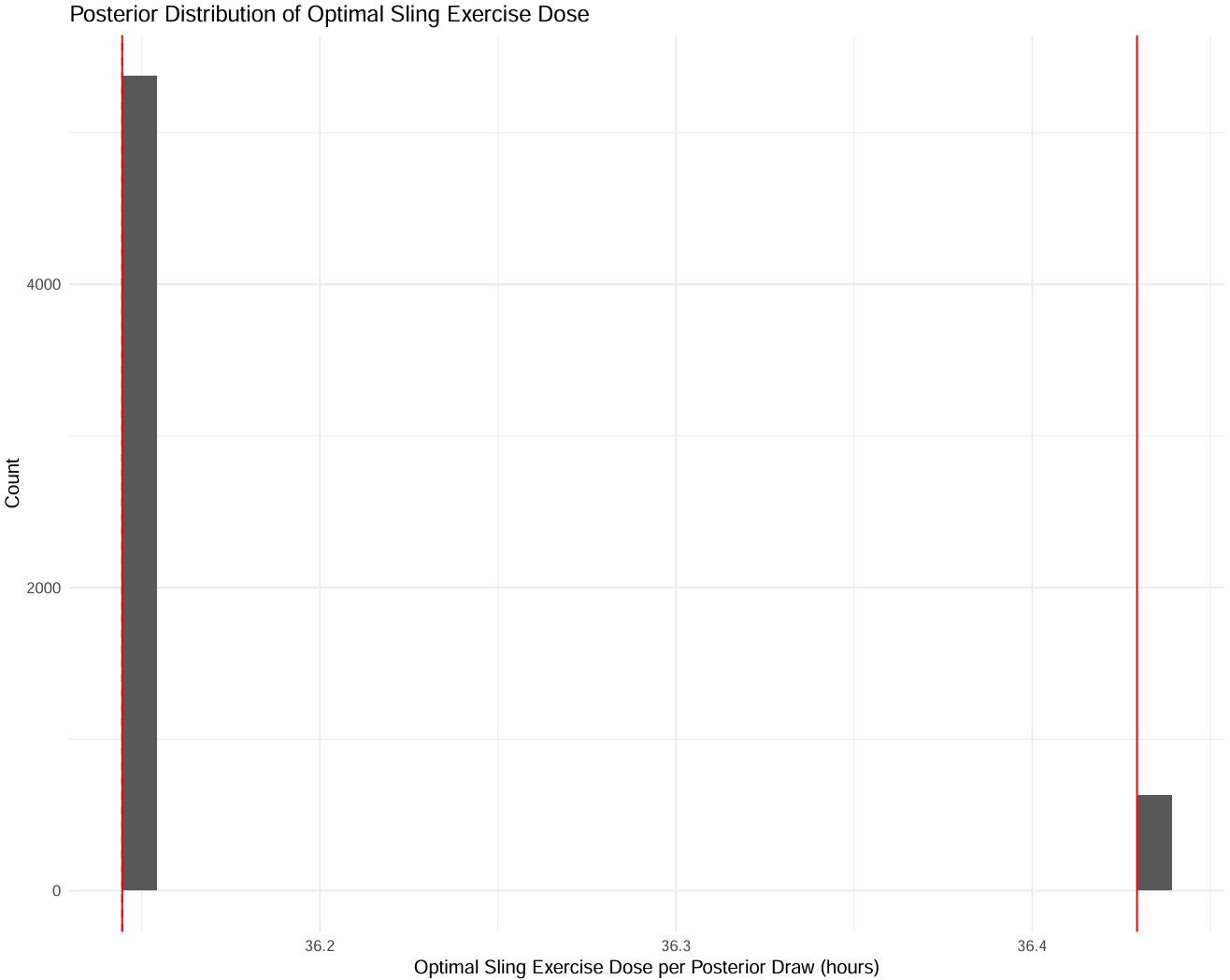


# Posterior Distribution of Optimal Sling Exercise Dose


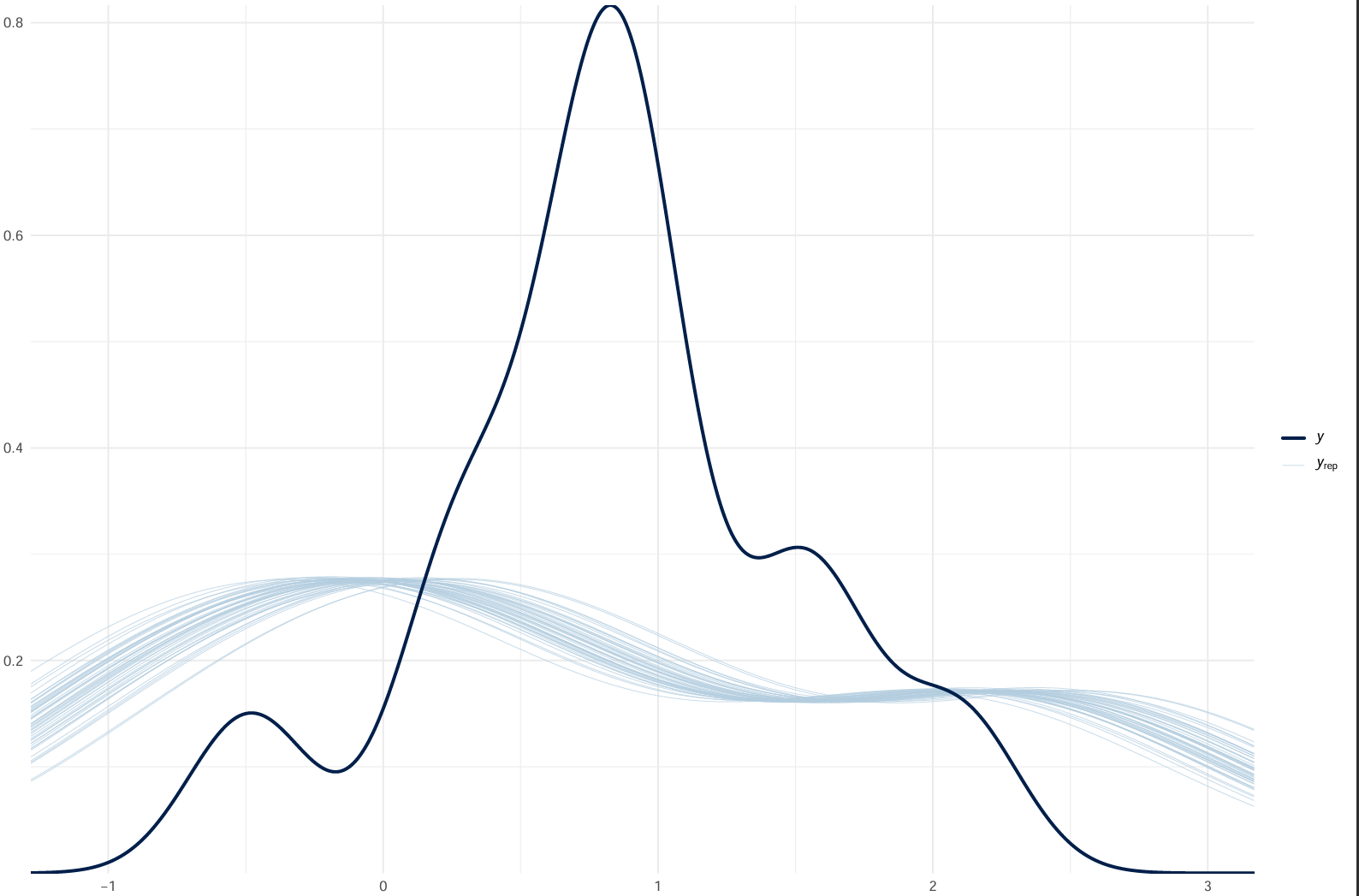


#
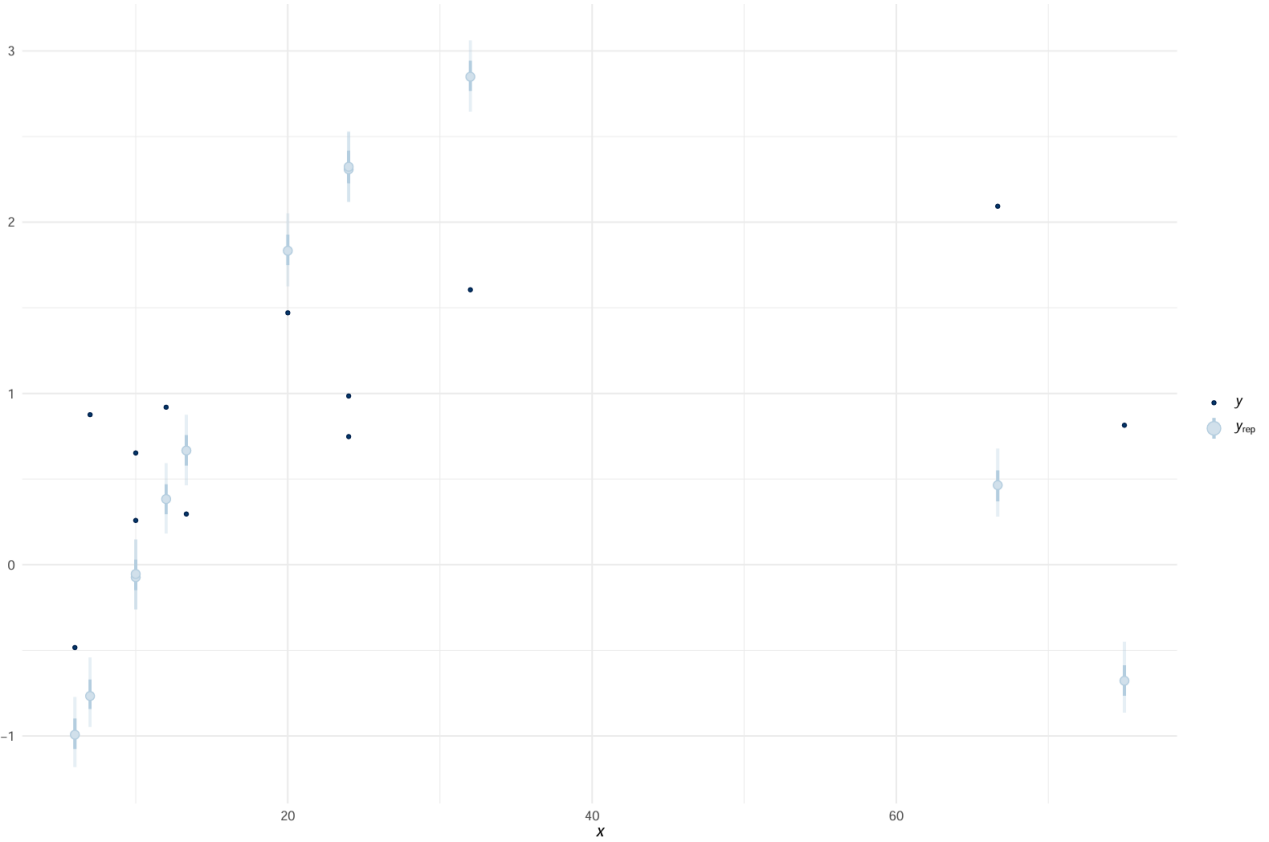
PPC: Density Overlay (Sling Exercise for Dynamic Balance)

# PPC: Observed vs Predicted SMD by Dose


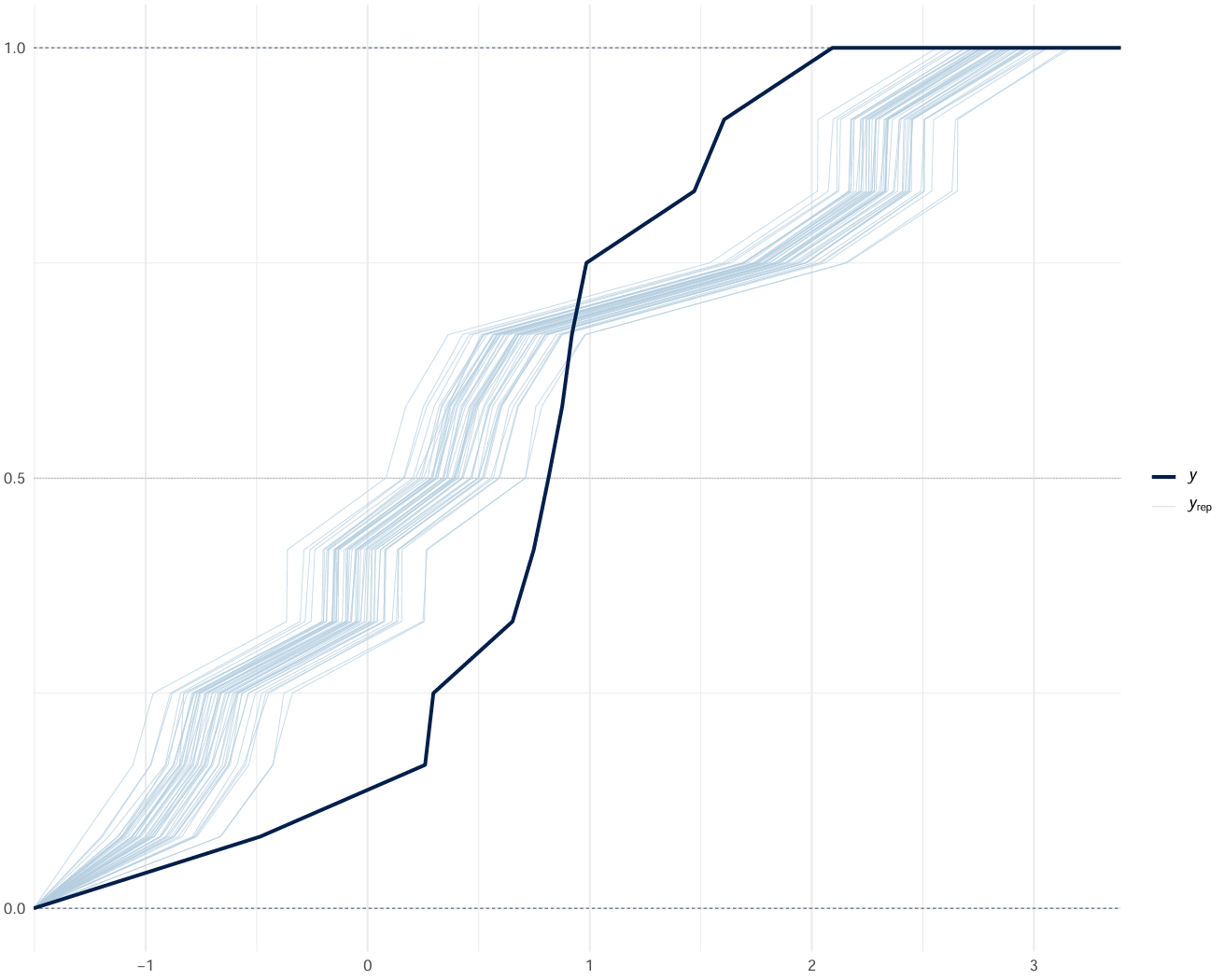


# PPC: ECDF Overlay (Sling Exercise for Dynamic Balance)


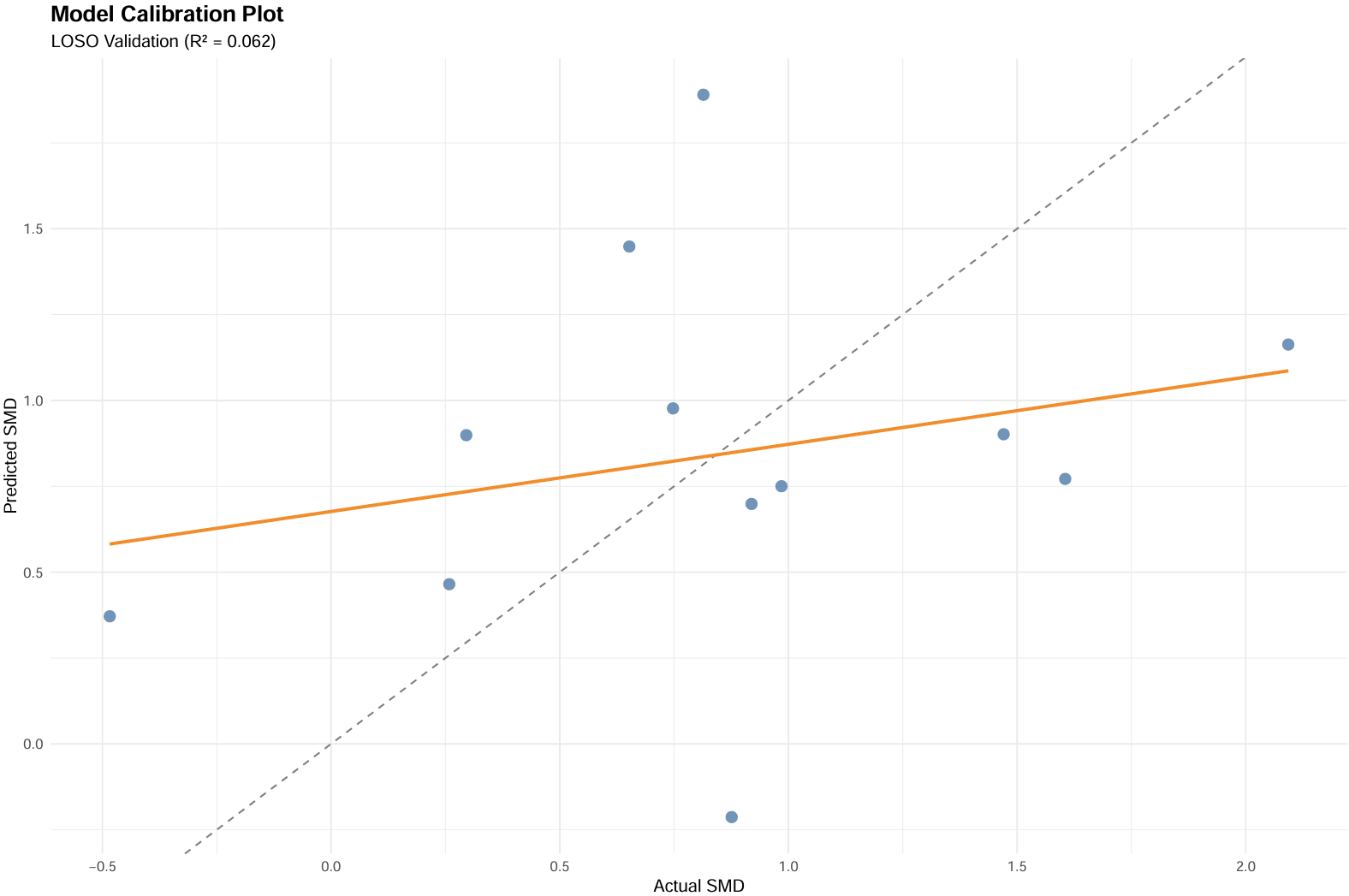


# Model Calibration Plot LOSO Validation (R² = 0.062)

#
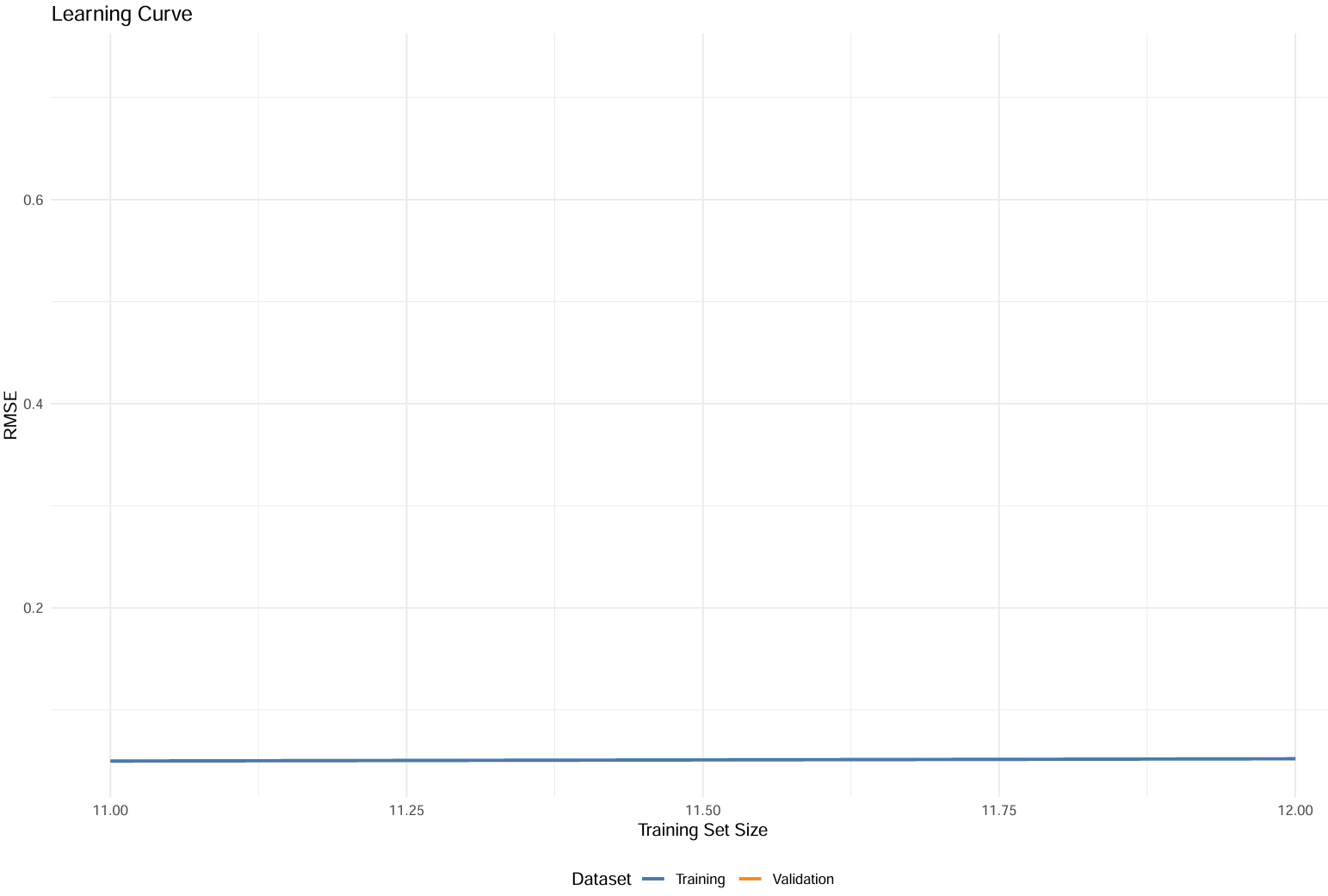
Learning Curve


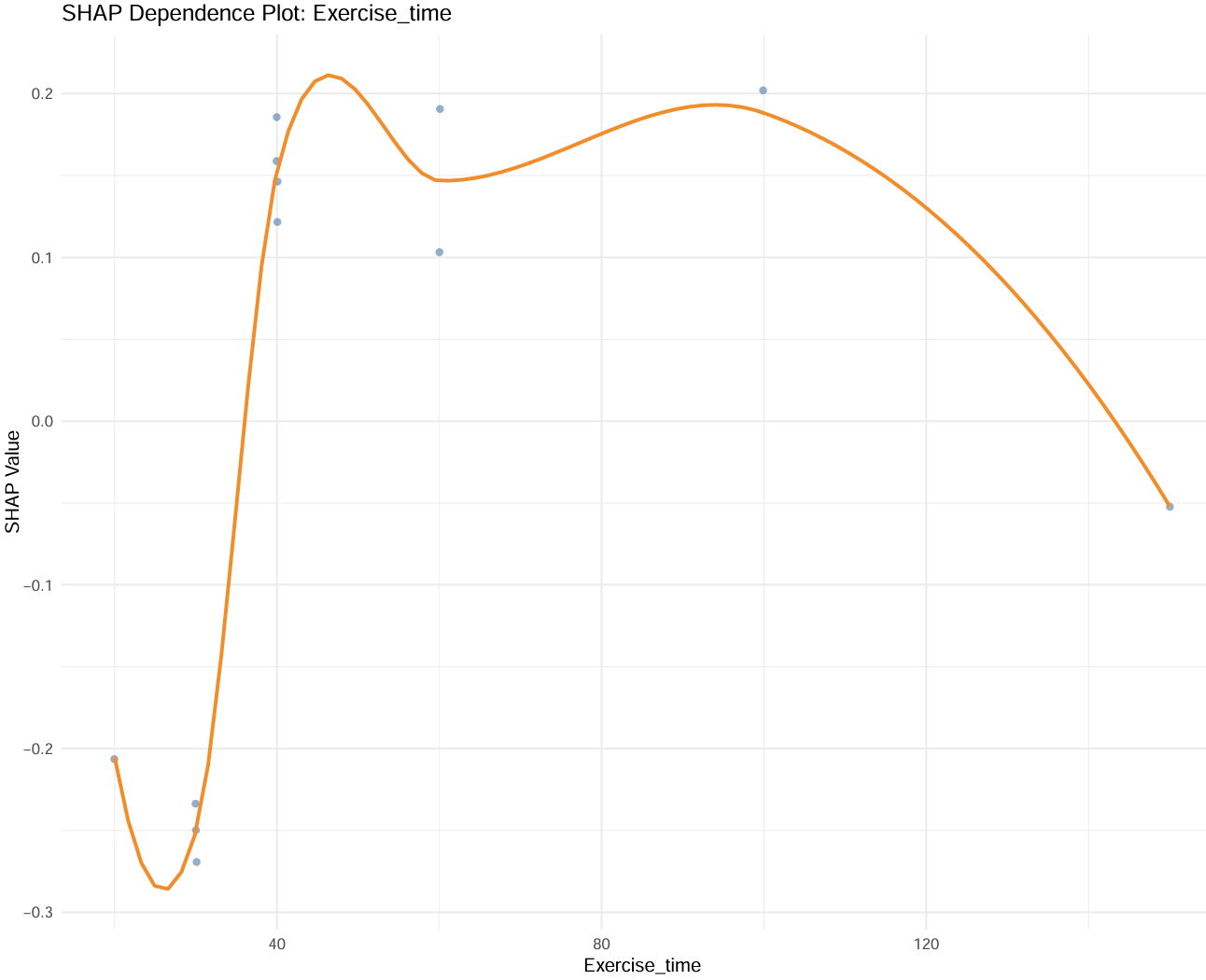


# SHAP Dependence Plot: Exercise_time


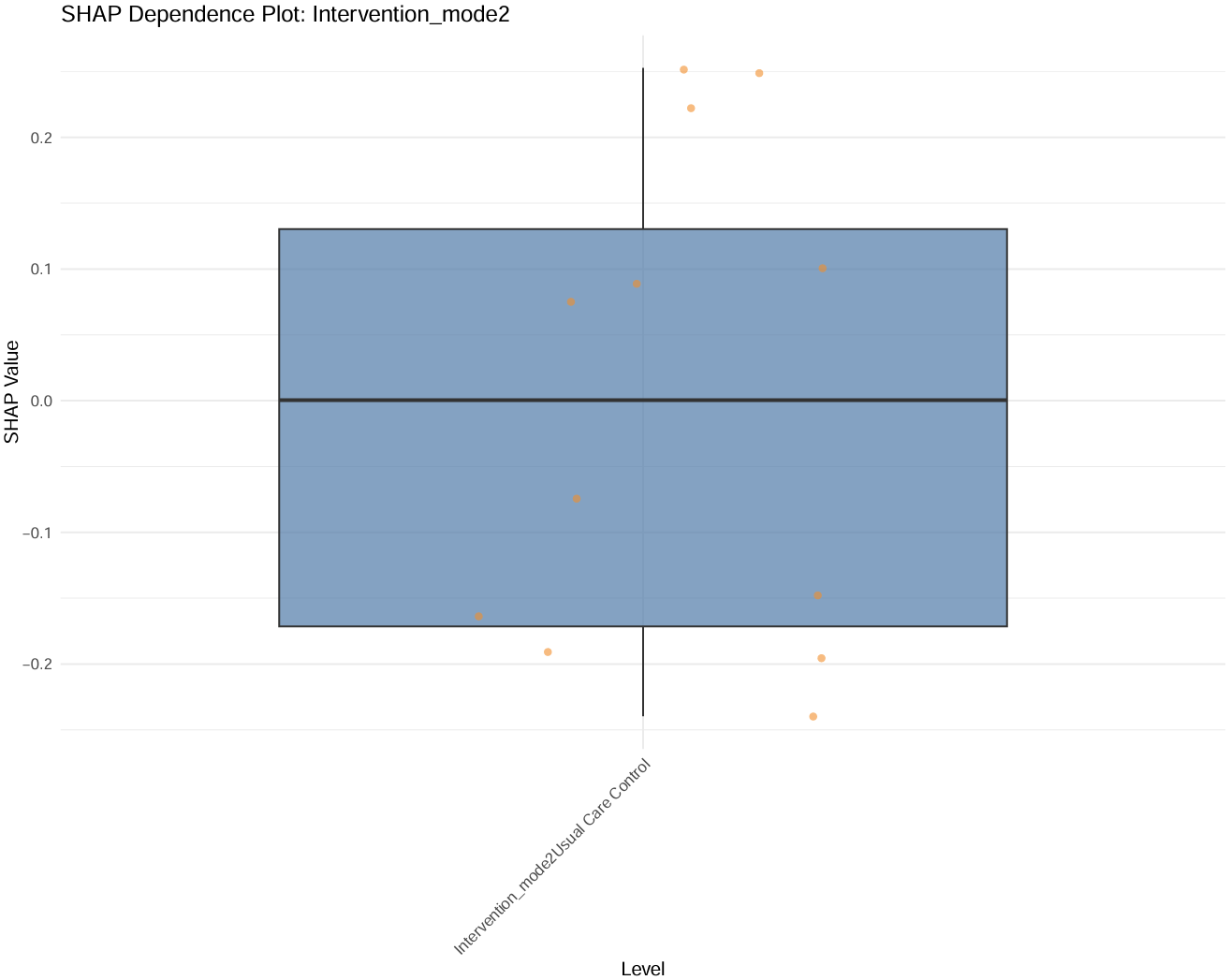


# SHAP Dependence Plot: Intervention_mode2

SHAP Dependence Plot: Intervention_mode1
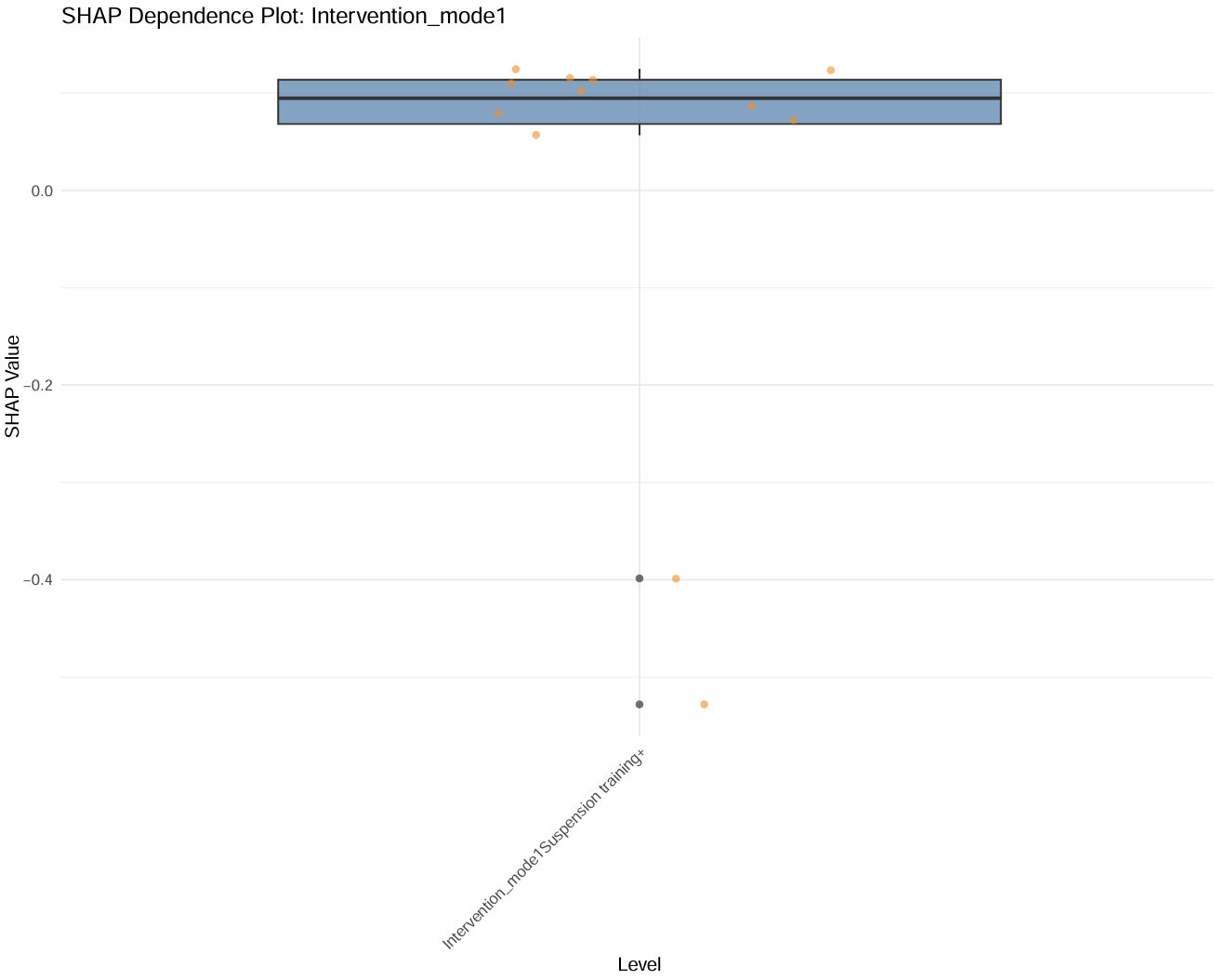


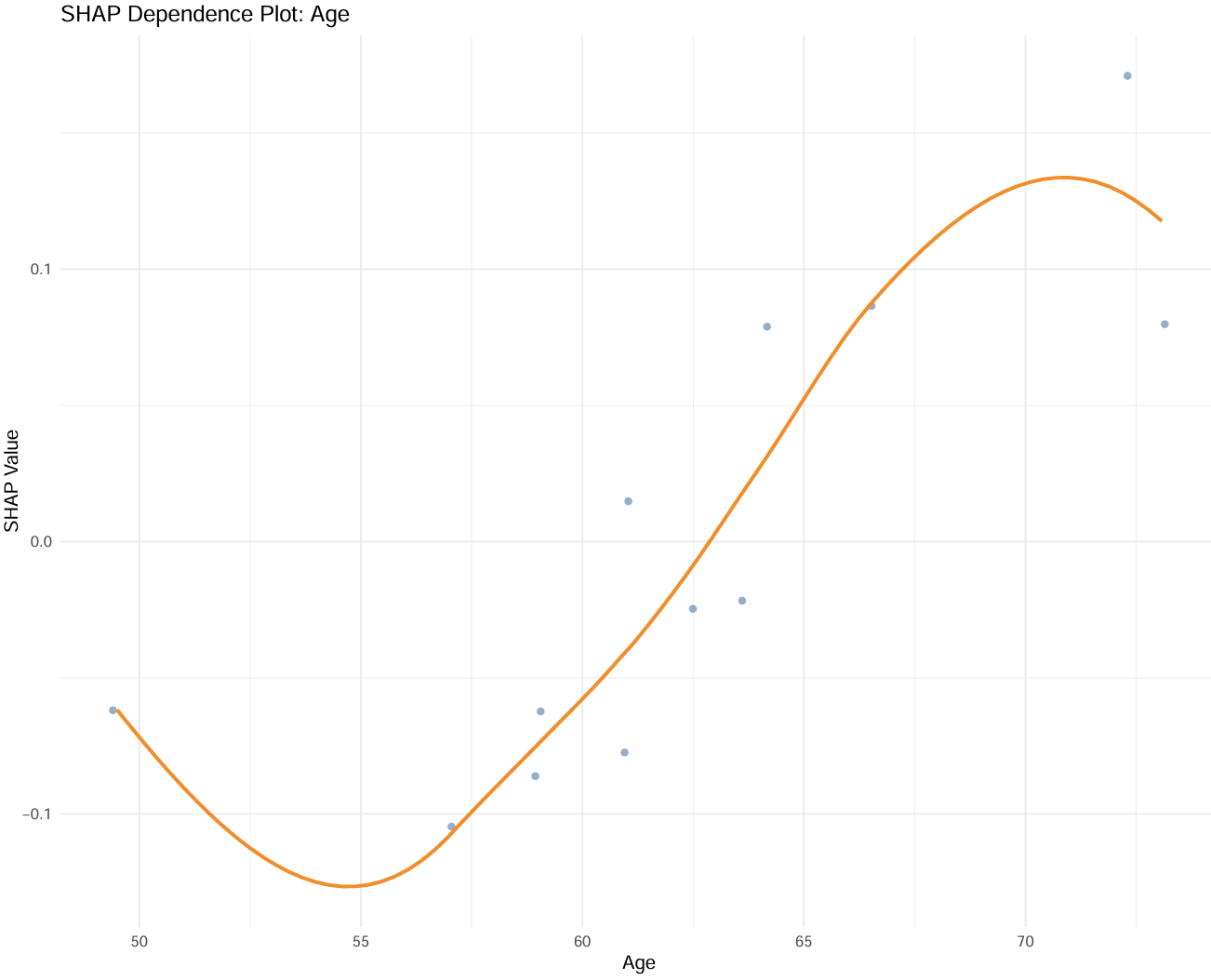


SHAP Dependence Plot: Age


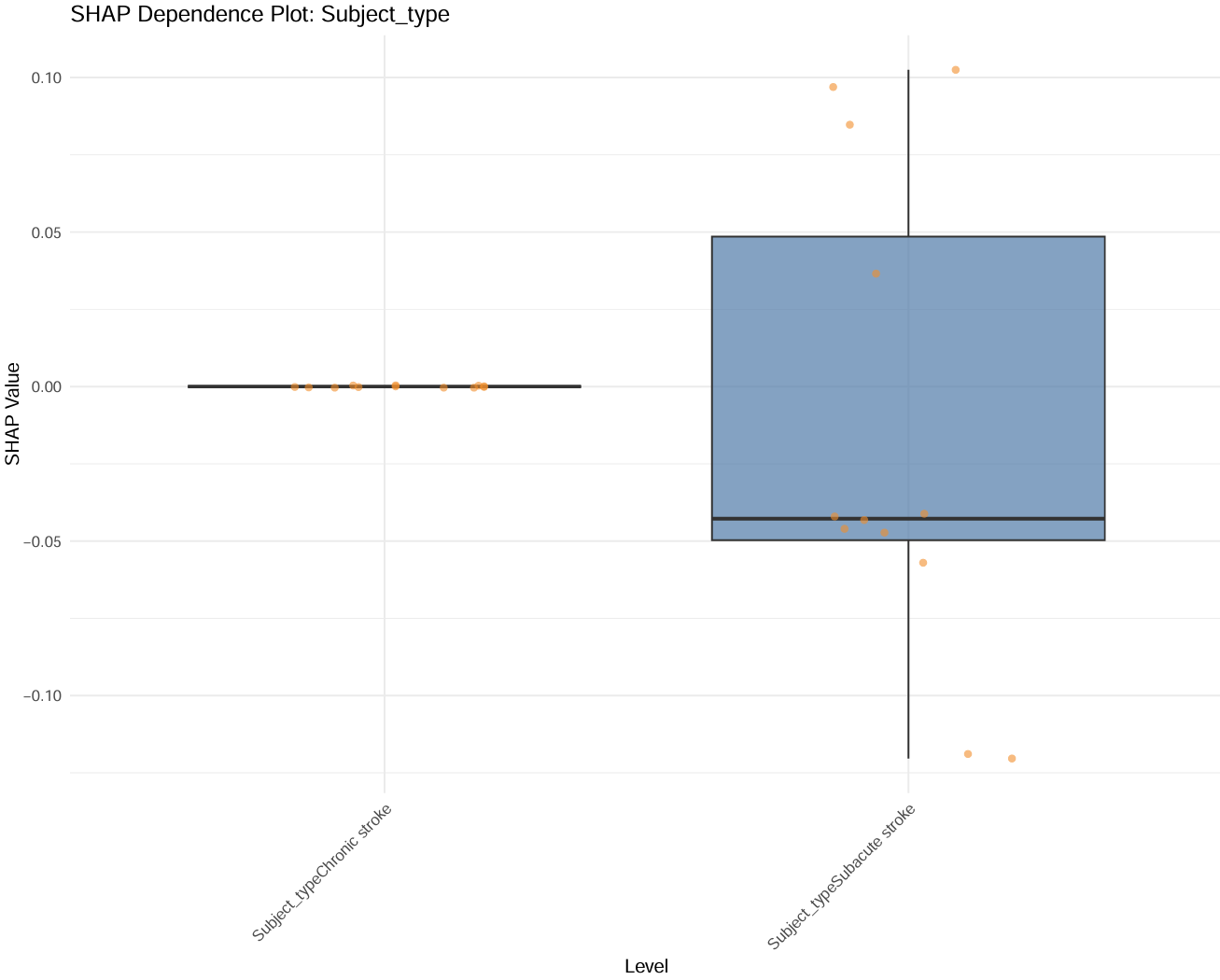


# SHAP Dependence Plot: Subject_type


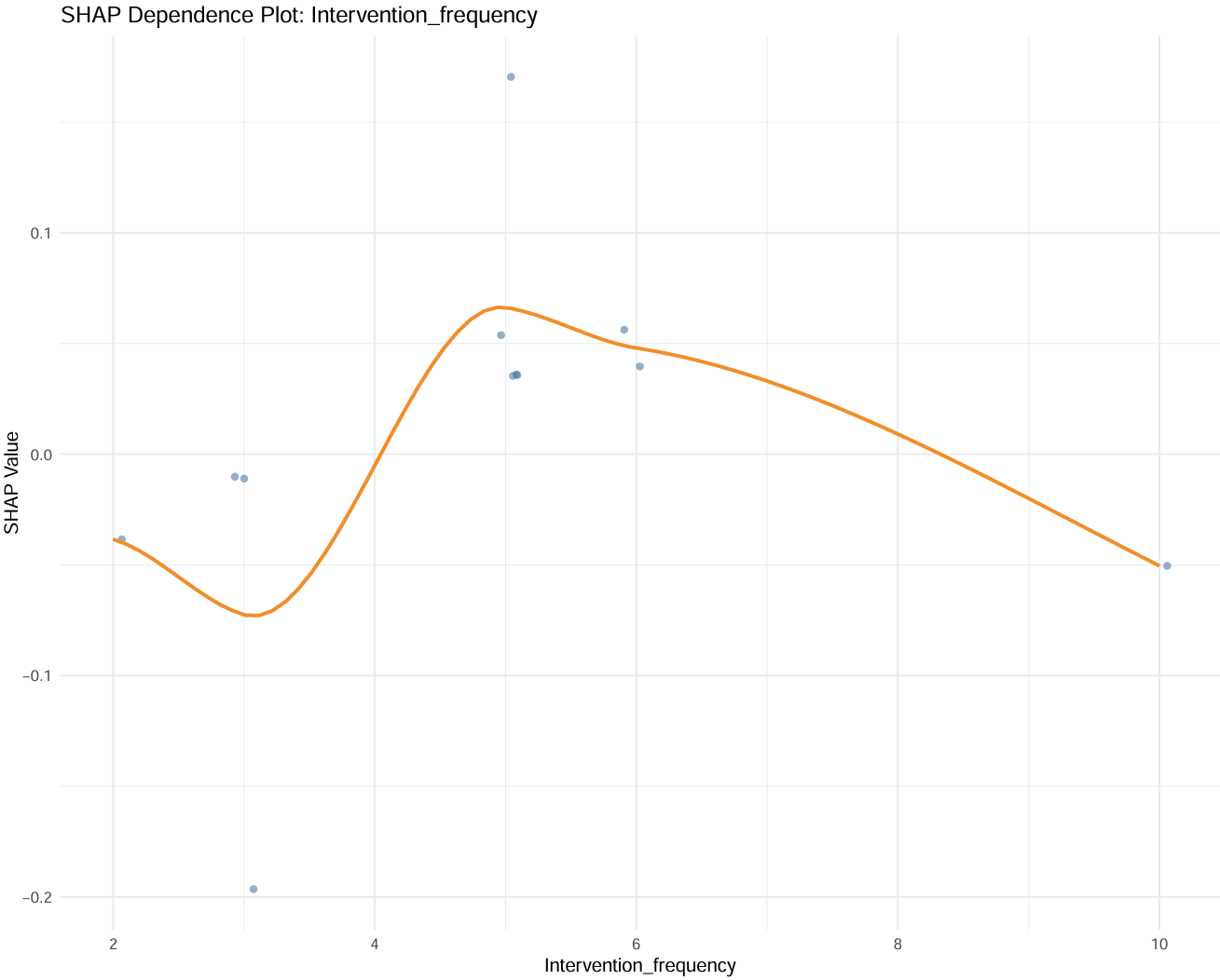


# SHAP Dependence Plot: Intervention_frequency


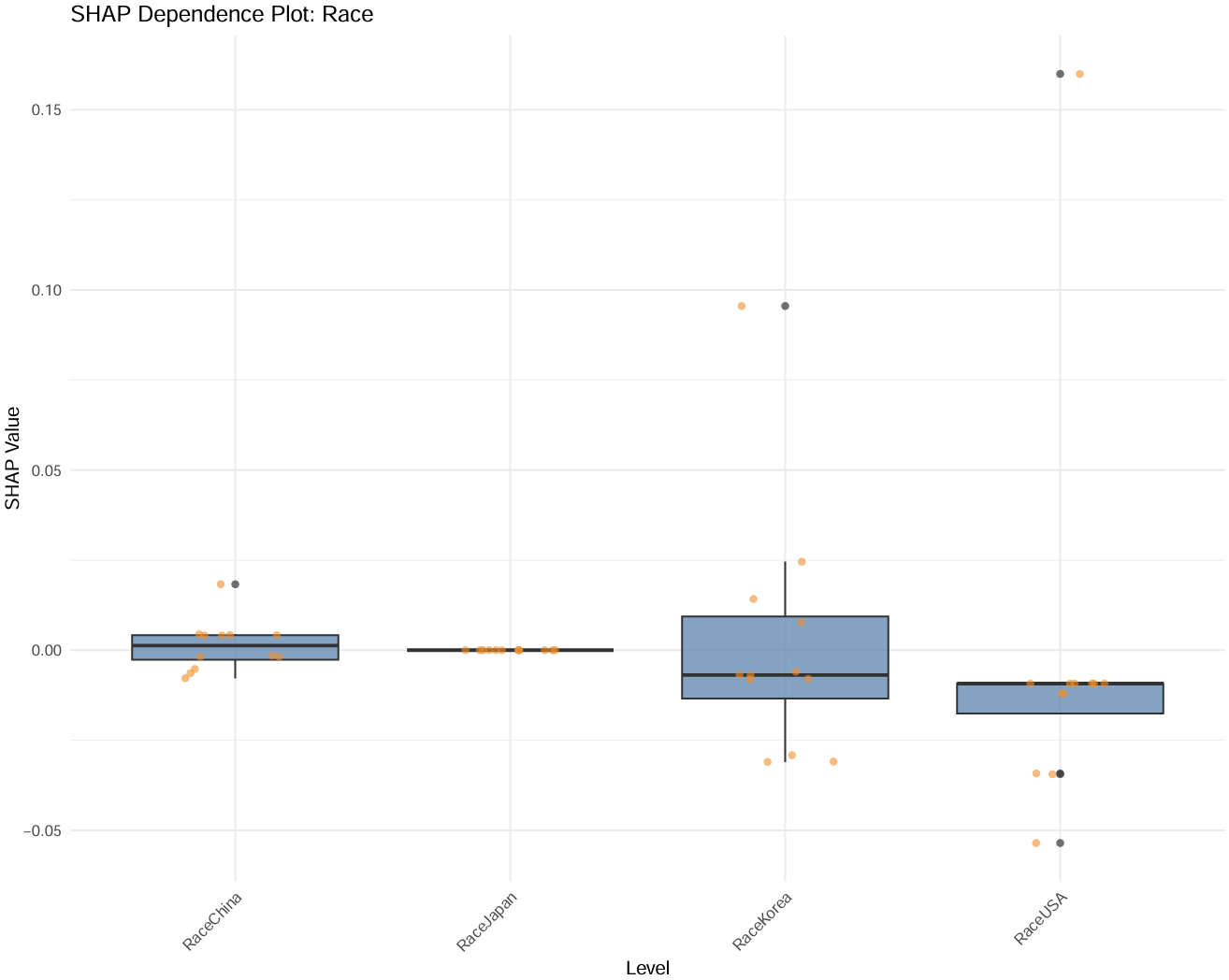


# SHAP Dependence Plot: Race


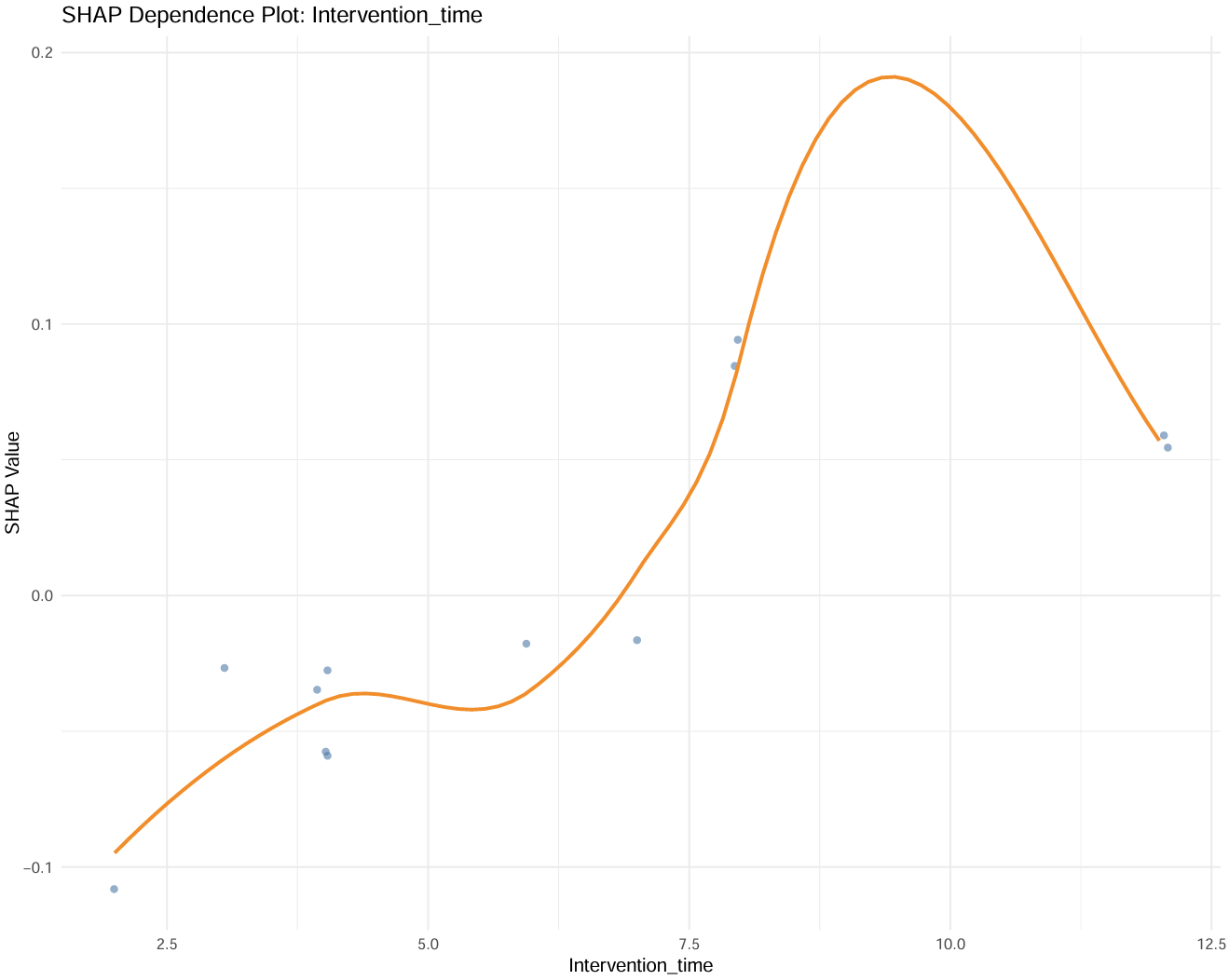


# SHAP Dependence Plot: Intervention_time


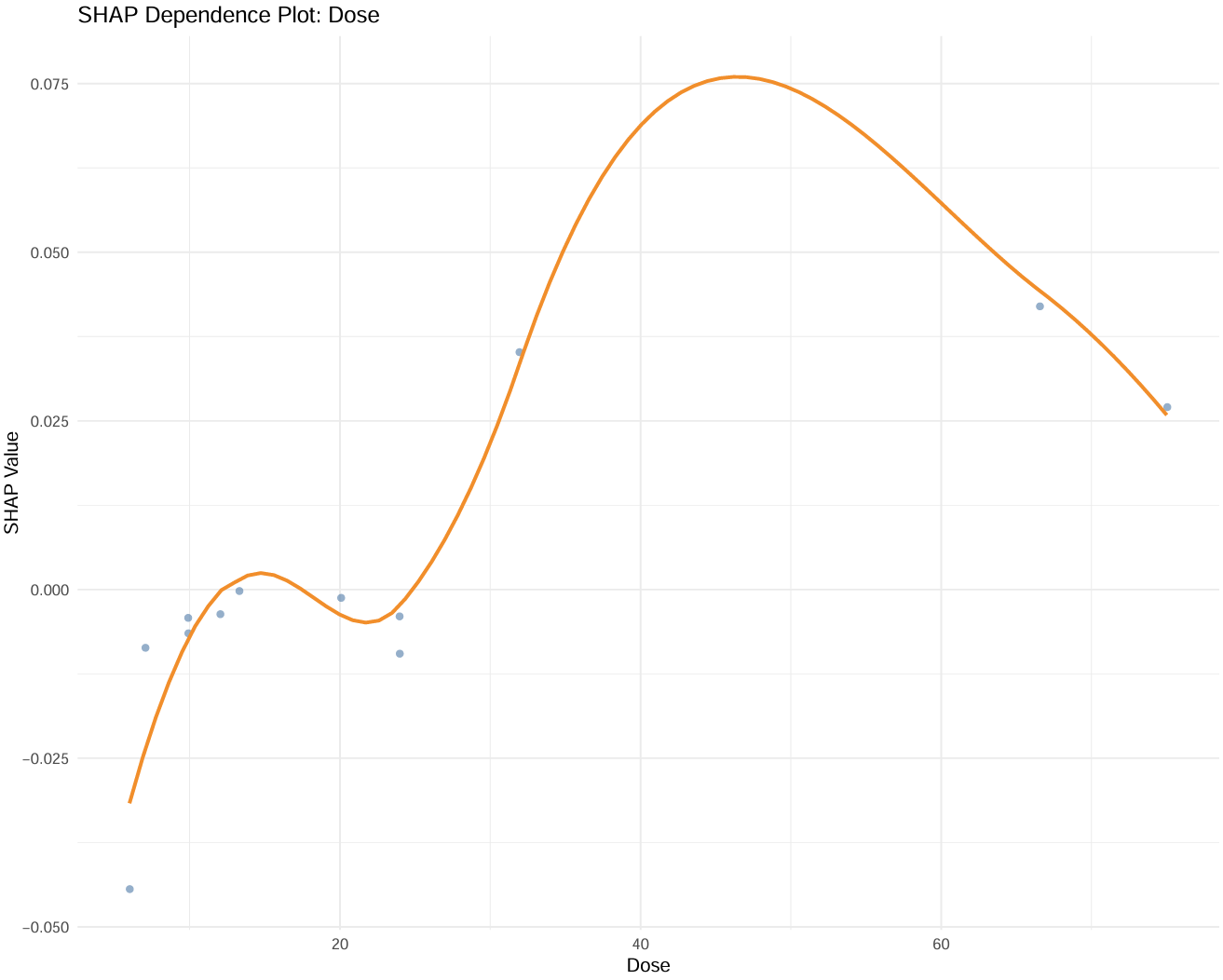


# SHAP Dependence Plot: Dose


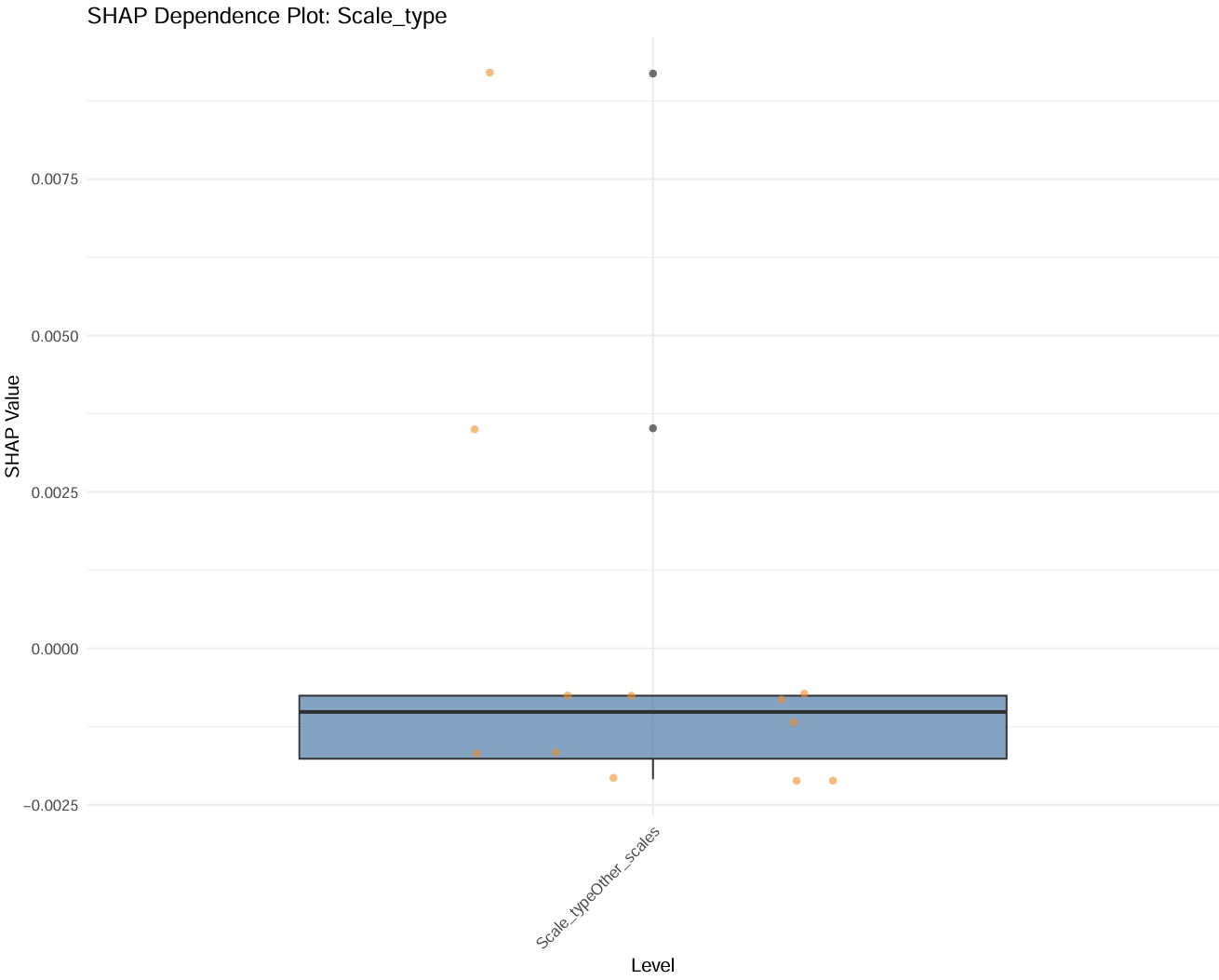


# SHAP Dependence Plot: Scale_type

# Table 2. Subgroup Analysis of Suspension Training in Improving Dynamic Balance in Stroke Patients

| Dimensionality | sort | K | N | I^2^ | Effect model | SMD and 95%CI | GRADE | P | P-interaction​​​ |
| --- | --- | --- | --- | --- | --- | --- | --- | --- | --- |
| Nation |  |  |  |  |  |  |  |  | 0.968 |
|  | China | 6 | 452 | 79.7% | Random | 0.97(0.54, 1.40) | Low | 0.001* |  |
|  | Korea | 4 | 100 | 84.8% | Random | 0.67(-0.42, 1.77) | Very Low | 0.230 |  |
|  | Japan | 1 | 22 | * | Random | 0.92(0.03, 1.81) | Low | 0.043* |  |
|  | USA | 1 | 14 | * | Random | 0.88(-0.24, 1.99) | Very Low | 0.123 |  |
| Intervention frequency/week |  |  |  |  |  |  |  |  | 0.509 |
|  | 2-3 | 4 | 133 | 58.2% | Random | 0.55(-0.08, 1.18) | Very Low | 0.086 |  |
|  | 5 | 5 | 191 | 73.7% | Random | 1.06(0.41, 1.71) | Moderate | 0.001* |  |
|  | 6-10 | 3 | 264 | 89.1% | Random | 0.96(0.15, 1.77) | Low | 0.021* |  |
| Intervention time/week |  |  |  |  |  |  |  |  | 0.004* |
|  | 2-4 | 6 | 245 | 71.4% | Random | 0.55(0.04, 1.06) | Moderate | 0.035* |  |
|  | 6-8 | 4 | 244 | 35.9% | Random | 1.54(1.15, 1.93) | Moderate | 0.001* |  |
|  | 12 | 2 | 99 | 0.0% | Random | 0.81(0.40, 1.22) | Moderate | 0.001* |  |
| Exercise time/minute |  |  |  |  |  |  |  |  | 0.006* |
|  | 20-30 | 4 | 126 | 21.5% | Random | 0.22(-0.13, 0.58) | Very Low | 0.218 |  |
|  | 40 | 4 | 301 | 68.1% | Random | 1.12(0.65, 1.59) | Moderate | 0.001* |  |
|  | 60 | 2 | 111 | 74.8% | Random | 1.06(0.26, 1.86) | Moderate | 0.009* |  |
|  | 100-150 | 2 | 50 | 66.8% | Random | 1.53(0.29, 2.78) | Moderate | 0.016* |  |
| Subject type |  |  |  |  |  |  |  |  | 0.020* |
|  | Chronic stroke | 5 | 161 | 48.6% | Random | 0.48(-0.00, 0.97) | Moderate | 0.050 |  |
|  | Subacute stroke | 4 | 329 | 72.2% | Random | 1.43(0.88, 1.97) | Moderate | 0.001* |  |
|  | Acute stroke | 3 | 98 | 0.0% | Random | 0.54(0.07, 1.02) | Moderate | 0.025* |  |
| Intervention mode 1 |  |  |  |  |  |  |  |  | 0.007* |
|  | Suspension training+ | 10 | 538 | 71.0% | Random | 1.05(0.70, 1.41) | Moderate | 0.001* |  |
|  | Suspension training | 2 | 50 | 38.0% | Random | -0.06(-0.78, 0.66) | Very Low | 0.862 |  |
| Intervention mode 2 |  |  |  |  |  |  |  |  | 0.029* |
|  | Usual Care Control | 6 | 253 | 42.8% | Random | 0.54(0.22, 0.87) | Moderate | 0.001* |  |
|  | Active Control | 6 | 335 | 68.3% | Random | 1.24(0.71, 1.78) | Moderate | 0.001* |  |
| Scale type |  |  |  |  |  |  |  |  | 0.454 |
|  | BBS | 10 | 530 | 78.9% | Random | 0.92(0.48, 1.36) | Low | 0.001* |  |
|  | Other_scales | 2 | 58 | 43.6% | Random | 0.60(-0.11, 1.31) | Very Low | 0.097 |  |
| Percentage of Body Weight Support |  |  |  |  |  |  |  |  | 0.813 |
|  | = 30% | 9 | 415 | 76.8% | Random | 0.90(0.41, 1.39) | Low | 0.001* |  |
|  | 30% - 50% | 3 | 173 | 78.8% | Random | 0.80(0.11, 1.48) | Low | 0.022* |  |
| Type of Suspension |  |  |  |  |  |  |  |  | 0.514 |
|  | Standard ST | 4 | 299 | 85.9% | Random | 1.20(0.15, 2.25) | Low | 0.025 |  |
|  | BWS | 5 | 213 | 0.9% | Random | 0.59(0.30, 0.87) | Moderate | 0.001 |  |
|  | RAGT | 3 | 76 | 0.0% | Random | 0.72(0.25, 1.18) | Moderate | 0.003 |  |

**Notes:** K: Number of studies; N: sample size; P-interaction​​​: interaction tests; Suspension training: Suspension Training alone; Suspension training +: Combined Treatment Strategies of Suspension Training with Other Interventions; Usual Care Control: Conventional Therapy; Active Control: Other Active Interventions Beyond Conventional Therapy; BBS: Berg Balance Scale; LOS: Limits of Stability; DGI: Dynamic gait index, ST: Traditional Suspension Training; BWS: Body Weight Supported Suspension Training; RAGT: Robot-Assisted Gait Training.

# Table 3. Detailed Data of Intervention Parameters

| author/years | Motion frequency | Intervention time | Exercise time | Total Intervention | Percentage of Body Weight Support | Intervention mode1 | Intervention mode2 | Subject type |
| --- | --- | --- | --- | --- | --- | --- | --- | --- |
| Lu, 2024a | 5 | 4 | 60 | 20 | 30%-50% | Suspension training+ | Active Control | Subacute stroke |
| Yu, 2020 | 3 | 12 | 40 | 24 | ≤ 30% | Suspension training+ | Usual Care Control | Chronic stroke |
| Chen, 2020 | 6 | 8 | 40 | 32 | ≤ 30% | Suspension training+ | Active Control | Subacute stroke |
| Huang, 2019 | 3 | 12 | 40 | 24 | ≤ 30% | Suspension training+ | Usual Care Control | Chronic stroke |
| Park, 2022 | 5 | 8 | 100 | 66.67 | ≤ 30% | Suspension training+ | Active Control | Subacute stroke |
| Choi, 2022 | 5 | 6 | 150 | 75 | ≤ 30% | Suspension training+ | Active Control | Chronic stroke |
| Lee, 2014 | 3 | 4 | 30 | 6 | ≤ 30% | Suspension training | Usual Care Control | Chronic stroke |
| Kim, 2017 | 5 | 4 | 30 | 10 | ≤ 30% | Suspension training | Active Control | Chronic stroke |
| TIAN, 2024 | 5 | 2 | 60 | 10 | 30%-50% | Suspension training+ | Usual Care Control | Subacute stroke |
| Lu, 2024b | 10 | 4 | 20 | 13.33 | ≤ 30% | Suspension training+ | Usual Care Control | Acute stroke |
| Takami, 2010 | 6 | 3 | 40 | 12 | ≤ 30% | Suspension training+ | Usual Care Control | Acute stroke |
| Park, 2020 | 2 | 7 | 30 | 7 | 30%-50% | Suspension training+ | Active Control | Acute stroke |

**Note:** Suspension training: Suspension Training alone; Suspension training+: Combined Treatment Strategies of Suspension Training with Other Interventions; Usual Care Control: Conventional Therapy; Active Control: Other Active Interventions Beyond Conventional Therapy; The calculation formula for the Total Intervention is: $Total Intervention=\frac{Motion frequency \times Intervention time \times Exercise time}{60}$; Acute stroke: Less than 1 month from the onset of the disease; Subacute stroke: 1 to 6 months from the onset of the disease; Chronic stroke: More than 6 months from the onset of the disease.

# Table 4. Trial Sequential Analysis (TSA) Parameter Settings

| Parameter | Setting | Description |
| --- | --- | --- |
| Test Type | Two-sided test | Standard significance testing |
| Alpha Level (α) | 0.05 | Probability of Type I error |
| Beta Level (β) | 0.02 | Probability of Type II error |
| Power (1-β) | 80% | Statistical power to detect the effect |
| Anticipated Effect Size | SMD = 0.431 | Based on current meta-analysis results (Conservative estimate) |
| Heterogeneity Level (I^2^) | 0.7% | Observed heterogeneity in TSA model |
| Heterogeneity Adjustment | 1.007 | Factor calculated based on observed I^2^ |
| Model Type | Random-effects Model | DerSimonian-Laird (DL) estimator |
| Monitoring Boundary | O'Brien-Fleming | Significance boundaries for sequential testing |
| Information Unit | Inverse Variance (1/SE^2^) | Calculated using variance-based method |

# Table 5. Trial Sequential Analysis (TSA) Summary of Results

| Indicator | Value | Note |
| --- | --- | --- |
| Required Information Size (RIS) | 42.456 | Minimum information units needed to detect effect |
| Current Cumulative Information | 143.607 | Total information provided by included studies |
| Information Fraction | 338.3% | Ratio of Current Information to RIS |
| Cumulative Z-score | 4.456 | Standard normal statistic of the pooled effect |
| Efficacy Boundaries | ± 1.960 | Critical value for two-sided α = 0.05 |
| RIS Reached? | Yes | Current Information > RIS |
| Boundary Crossed? | Yes | Z-curve crossed the efficacy boundary |
| Evidence Status | Conclusive Evidence | Both RIS reached and boundary crossed |
| Recommendation | No further studies needed | Evidence is sufficient and robust |

#
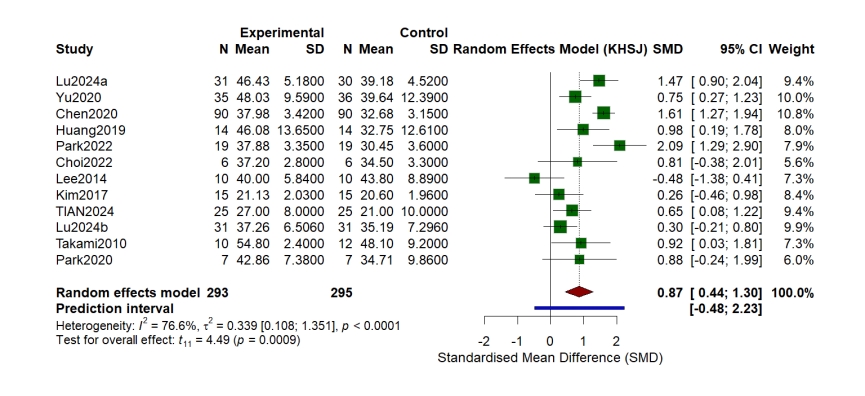
Forest plot of the KHSJ random effects model analysis


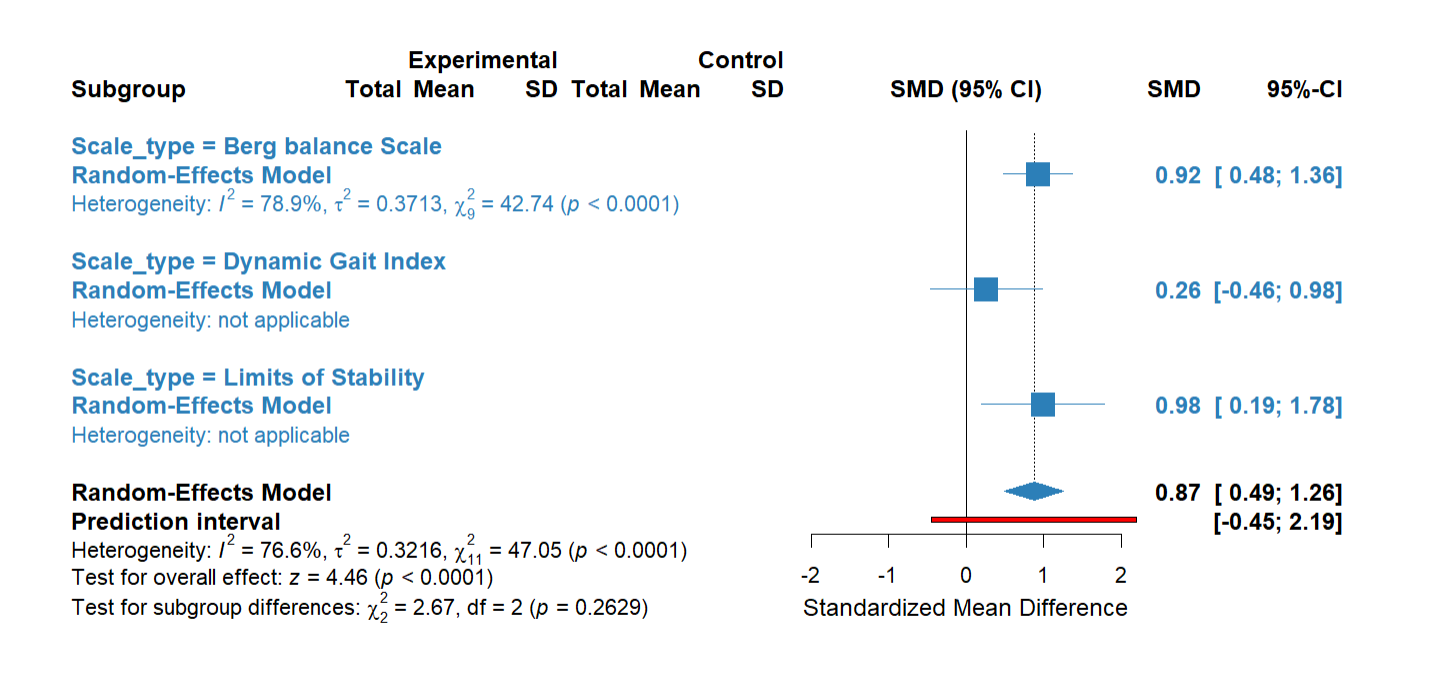
Direction of SMD in different scales

#
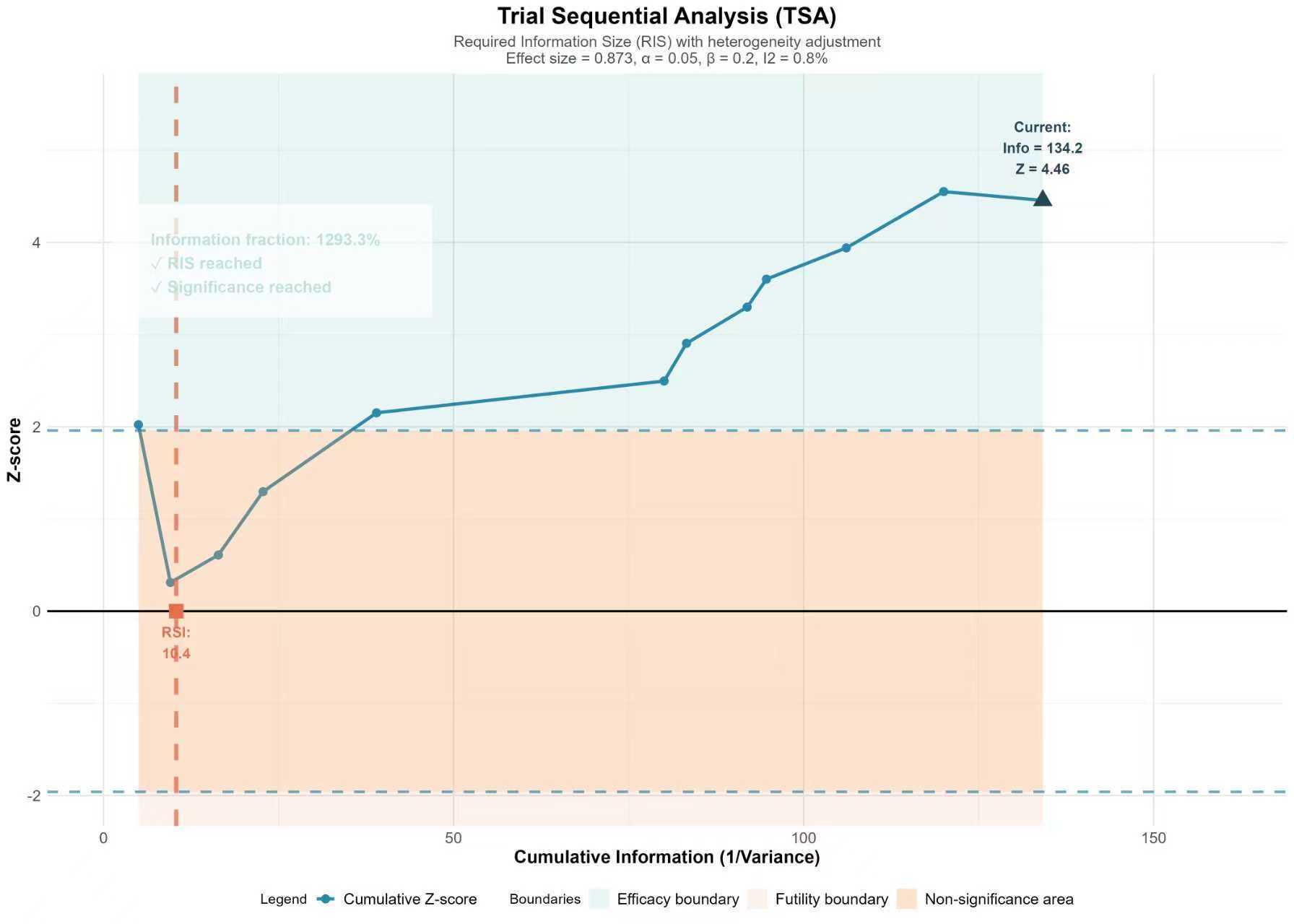
Trial Sequential Analysis (TSA)

# Inter-Rater Agreement Results for Each ROB2 Domain

The table below displays the simple agreement rate, Cohen's Kappa, and Weighted Kappa calculated separately for each domain (D1-D5).

| Domain | Simple Agreement Rate | Cohen's Kappa | Weighted Kappa | Strength of Agreement (Cohen's Kappa) |
| --- | --- | --- | --- | --- |
| D1 (Randomization process) | 58.3% (7/12) | 0.250 | 0.250 | Fair |
| D2 (Deviations from intended interventions) | 100% (12/12) | 1.000 | 1.000 | Perfect |
| D3 (Missing outcome data) | 91.7% (11/12) | 0.840 | 0.867 | Perfect |
| D4 (Measurement of the outcome) | 83.3% (10/12) | 0.676 | 0.707 | Good |
| D5 (Selection of the reported result) | 91.7% (11/12) | 0.000 | 0.000 | Slight |

**Note:** The inter-rater agreement between the two reviewers for each ROB2 domain was assessed. The Simple Agreement Rate​ was calculated as the percentage of studies for which both reviewers assigned identical judgments (Low, Some concerns, or High). Cohen's Kappa (κ)​ statistic, which accounts for chance agreement, was calculated from 3x3 contingency tables. Weighted Kappa​ was also computed using linear weights (0, 0.5, 1) to grant partial credit for adjacent rating disagreements (e.g., Low vs. Some concerns). The strength of agreement based on Cohen's Kappa was interpreted as follows: 0.81–1.00 = "Perfect/Almost Perfect", 0.61–0.80 = "Substantial/Good", 0.41–0.60 = "Moderate", 0.21–0.40 = "Fair", 0.00–0.20 = "Slight", and <0.00 = "Poor".

# **GRADE Summary**

| **GRADE domain** | **Judgment** | **Rationale** |
| --- | --- | --- |
| Risk of bias | Serious (downgraded 1 level) | Using RoB 2.0, concerns were mainly concentrated in D1 (randomization process) and D4 (outcome measurement). Several trials provided insufficient details on random sequence generation/allocation concealment and on measurement procedures or assessor blinding, which could plausibly bias effect estimates. Selective reporting was largely low risk (D5). We therefore downgraded one level for risk of bias. |
| Inconsistency | Not downgraded | Between-study heterogeneity was substantial (I² = 76.6%), but the direction and statistical significance of the pooled effect were consistent. Robustness checks (leave-one-out sensitivity analysis; influence diagnostics; trim-and-fill) did not materially change the main conclusion, and trial sequential analysis suggested the accumulated information size exceeded the required information size. Therefore, we did not downgrade for inconsistency. |
| Indirectness | Not downgraded | The population (stroke patients), intervention (suspension training alone or combined), comparators (usual care or other active rehabilitation), and outcomes (validated dynamic balance scales) directly matched the review question; no serious indirectness was identified. |
| Imprecision | Not downgraded | The pooled estimate was clinically interpretable and excluded the null (SMD = 0.87, 95% CI 0.49 to 1.26). Trial sequential analysis indicated adequate information (RIS = 10.3; cumulative information size = 124.3; information ratio = 12.07; cumulative Z = 11.1 crossing conventional boundaries), supporting sufficient precision. |
| Publication bias | Not downgraded | Egger’s regression did not indicate small-study effects (t = −1.217, P = 0.252). The trim-and-fill procedure suggested two potentially missing studies, but the adjusted estimate remained directionally consistent and statistically significant, and sensitivity analyses supported stability. Therefore, we did not downgrade for publication bias. |
| Overall quality of evidence | Moderate | All included studies were randomized controlled trials (starting level: high). We downgraded one level for risk of bias (concerns concentrated in D1 and D4). We did not downgrade for inconsistency, indirectness, imprecision, or publication bias based on the available robustness checks and trial sequential analysis. Consequently, the overall GRADE rating was Moderate. |
